# Supplementary material for: Interventions and strategies involving primary healthcare professionals to manage emergency department overcrowding: a scoping review
Source: BMJ Open. 2021 May 10;11(5):e048613. doi: 10.1136/bmjopen-2021-048613 (PMC8112422; doi:10.1136/bmjopen-2021-048613)
Supplement: Author's manuscript [file bmjopen-2021-048613.draft_revisions.pdf]

BMJ Open is committed to open peer review. As part of this commitment we make the peer review history of every article we publish publicly available.

When an article is published we post the peer reviewers' comments and the authors' responses online. We also post the versions of the paper that were used during peer review. These are the versions that the peer review comments apply to.

The versions of the paper that follow are the versions that were submitted during the peer review process. They are not the versions of record or the final published versions. They should not be cited or distributed as the published version of this manuscript.

BMJ Open is an open access journal and the full, final, typeset and author-corrected version of record of the manuscript is available on our site with no access controls, subscription charges or pay-per-view fees (http://bmjopen.bmj.com).

If you have any questions on BMJ Open's open peer review process please email info.bmjopen@bmj.com

BMJ Open

Interventions and strategies involving primary healthcare professionals to manage emergency department overcrowding: a scoping review

Journal:	*BMJ Open*
Manuscript ID	bmjopen-2021-048613
Article Type:	Original research
Date Submitted by the Author:	03-Jan-2021

Complete List of Authors:	Jeyaraman, Maya; University of Manitoba, George and Fay Yee Center for Healthcare Innovation<br>Copstein, Leslie; University of Manitoba, George & Fay Yee Center for Healthcare Innovation<br>Al-Yousif, Nameer; University of Manitoba, George & Fay Yee Center for Healthcare Innovation<br>Alder, Rachel; University of Manitoba, Max Rady College of Medicine, Rady Faculty of Health Sciences,<br>Kirkland, Scott ; University of Alberta, Department of Emergency Medicine, Faculty of Medicine & Dentistry,<br>Al-Yousif, Yahya; University of Manitoba, George & Fay Yee Center for Healthcare Innovation<br>Suss, Roger; University of Manitoba, Department of Family Medicine, Rady Faculty of Health Sciences,<br>Zarychanski, Ryan ; CancerCare Manitoba, Department of Medical Oncology and Hematology<br>Doupe, Malcolm; University of Manitoba, Department of Community Health Sciences, Rady Faculty of Health Sciences,<br>Berthelot, Simon; Centre de recherche du CHU de Québec-Université Laval, Axe Santé des populations et Pratiques optimales en santé,<br>Mireault, Jean; Université de Montréal, HEC Pôle santé,<br>Tardif, Patrick; Department of Emergency Medicine, Cité de la santé de Laval,<br>Askin, Nicole; University of Manitoba, WRHA Virtual Library,<br>Buchel, Tamara; Manitoba College of Family Physicians<br>Rabbani, Rasheda; University of Manitoba, George and Fay Yee Center for Healthcare Innovation,<br>Beaudry, Thomas; George & Fay Yee Center for Healthcare Innovation, Patient and Public Engagement Collaborative Partnership,<br>Hartwell, Melissa; The Alberta Primary and Integrated Health care Innovation Network<br>Shimmin, Carolyn; University of Manitoba, George and Fay Yee Center for Healthcare Innovation,<br>Edwards, Jeanette; Shared Health Manitoba, Department of Community Health Quality and Learning,<br>Halas , Gayle; Manitoba Primary and Integrated Health care Innovation Network<br>Sevcik, William; University of Alberta, Department of Emergency Medicine, Faculty of Medicine & Dentistry,

Tricco, Andrea; St Michael's Hospital Li Ka Shing Knowledge Institute, Knowledge Translation Program, Unity Health Toronto,
Chochinov, Alecs; University of Manitoba, Department of Emergency Medicine, Faculty of Medicine,
Rowe, Brian; University of Alberta, Department of Emergency Medicine, Faculty of Medicine & Dentistry and School of Public Health,
Abou-Setta, Ahmed; University of Manitoba, George and Fay Yee Center for Healthcare Innovation

Keywords: ACCIDENT & EMERGENCY MEDICINE, PRIMARY CARE, PUBLIC HEALTH

I, the Submitting Author has the right to grant and does grant on behalf of all authors of the Work (as defined in the below author licence), an exclusive licence and/or a non-exclusive licence for contributions from authors who are: i) UK Crown employees; ii) where BMJ has agreed a CC-BY licence shall apply, and/or iii) in accordance with the terms applicable for US Federal Government officers or employees acting as part of their official duties; on a worldwide, perpetual, irrevocable, royalty-free basis to BMJ Publishing Group Ltd ("BMJ") its licensees and where the relevant Journal is co-owned by BMJ to the co-owners of the Journal, to publish the Work in this journal and any other BMJ products and to exploit all rights, as set out in our licence.

The Submitting Author accepts and understands that any supply made under these terms is made by BMJ to the Submitting Author unless you are acting as an employee on behalf of your employer or a postgraduate student of an affiliated institution which is paying any applicable article publishing charge ("APC") for Open Access articles. Where the Submitting Author wishes to make the Work available on an Open Access basis (and intends to pay the relevant APC), the terms of reuse of such Open Access shall be governed by a Creative Commons licence – details of these licences and which Creative Commons licence will apply to this Work are set out in our licence referred to above.

Other than as permitted in any relevant BMJ Author's Self Archiving Policies, I confirm this Work has not been accepted for publication elsewhere, is not being considered for publication elsewhere and does not duplicate material already published. I confirm all authors consent to publication of this Work and authorise the granting of this licence.

For peer review only

Interventions and strategies involving primary healthcare professionals to manage emergency department overcrowding: a scoping review

Review Authors: Maya M. Jeyaraman[1], Leslie Copstein[1], Nameer Al-Yousif[1], Rachel N. Alder[2] Scott W. Kirkland[3], Yahya Al-Yousif[1], Roger Suss[4], Ryan Zarychanski[5], Malcolm Doupe[6], Simon Berthelot[7], Jean Mireault[8], Patrick Tardif[9], Nicole Askin[10], Tamara Buchel[11], Rasheda Rabbani[1], Thomas Beaudry[12], Melissa Hartwell[13], Carolyn Shimmin[1], Jeanette Edwards[14], Gayle Halas[15], William Sevcik[16], Andrea C. Tricco[17], Alecs Chochinov[18], Brian H. Rowe[19], Ahmed M. Abou-Setta[1]

Affiliations:

[1]George & Fay Yee Center for Healthcare Innovation, University of Manitoba, Winnipeg, Manitoba, Canada

[2] Max Rady College of Medicine, Rady Faculty of Health Sciences, University of Manitoba, Winnipeg, Manitoba, Canada

[3] Department of Emergency Medicine, Faculty of Medicine & Dentistry, University of Alberta, Edmonton, Alberta, Canada

[4] Department of Family Medicine, Rady Faculty of Health Sciences, University of Manitoba, Winnipeg, Manitoba, Canada

[5] Department of Medical Oncology and Hematology, Cancer Care Manitoba, Winnipeg, Manitoba, Canada

[6] Department of Community Health Sciences, University of Manitoba, Winnipeg, Manitoba, Canada

[7] Centre de recherche du CHU de Québec-Université Laval, Axe Santé des populations et Pratiques optimales en santé, Laval, Québec, Canada.

[8] HEC Pôle santé, Université de Montréal, Montreal, Quebec, Canada

[9] Department of Emergency Medicine, Cité de la santé de Laval, Laval, Quebec, Canada

[10] WRHA Virtual Library, University of Manitoba, Winnipeg, Manitoba, Canada

[11] Manitoba College of Family Physicians, Winnipeg, Manitoba. Canada

[12] Patient and Public Engagement Collaborative Partnership, George & Fay Yee Center for Healthcare Innovation, Winnipeg, Manitoba, Canada

For peer review only - http://bmjopen.bmj.com/site/about/guidelines.xhtml

[13] The Alberta Primary and Integrated Health care Innovation Network, Edmonton, Alberta, Canada

[14] Department of Community Health Quality and Learning, Shared Health Manitoba, Winnipeg, Manitoba, Canada

[15] Manitoba Primary and Integrated Health care Innovation Network, Winnipeg, Manitoba, Canada

[16] Department of Emergency Medicine, Faculty of Medicine & Dentistry, University of Alberta, Edmonton, Alberta, Canada.

[17] Knowledge Translation Program, St. Michael's Hospital Li Ka Shing Knowledge Institute, Unity Health Toronto, Toronto, Ontario, Canada

[18] Department of Emergency Medicine, Faculty of Medicine, University of Manitoba, Winnipeg, Manitoba, Canada

[19] Department of Emergency Medicine, Faculty of Medicine & Dentistry and School of Public Health, University of Alberta, Edmonton, Alberta, Canada.

Corresponding author: Maya Jeyaraman, MD PhD
George & Fay Yee Center for Healthcare Innovation
753 Mc Dermot Avenue, Winnipeg, MB, R3E 0T6, Canada
Email: maya.jeyaraman@umanitoba.ca
Phone: (204) 594-5362; Fax: (204) 594-5394.

Running Title: Interventions involving primary healthcare professionals to manage emergency department overcrowding

Keywords: emergency department overcrowding, primary healthcare professionals, patient engagement

Total word count: 3670 words; **Abstract**: 281 words**; Figures**: 2; **Total tables**: 6 (Main tables: 2; Appendix tables: 4)

For peer review only - http://bmjopen.bmj.com/site/about/guidelines.xhtml

Abstract

Objectives: To conduct a scoping review to identify and summarize the existing literature on interventions involving primary healthcare professionals to manage emergency department overcrowding

Design: Scoping review

Data sources: A comprehensive database search of Medline (Ovid), EMBASE (Ovid), Cochrane Library (Wiley), and CINAHL (EBSCO)) databases was conducted (inception until January 2020) using peer-reviewed search strategies, complemented by a search of grey literature sources.

Eligibility criteria: Interventions and strategies involving primary healthcare professionals (PHCPs: general practitioners [GP], nurse practitioners [NP], or nurses with expanded role) to manage emergency department (ED) overcrowding.

Methods: We engaged and collaborated, with 13 patient partners during the design and conduct stages of this review. We conducted this review using the Joanna Briggs Institute guidelines. Two reviewers independently selected studies and extracted data. We conducted descriptive analysis of the included studies (frequencies and percentages).

Results: From 23,947 records identified, we included 268 studies published between 1981 to 2020. The majority (58%) of studies were conducted in North America and were predominantly cohort studies (42%). The reported interventions were either "within ED" (48%) interventions (e.g., PHCP-led ED triage or fast track), or "outside ED" (52%) interventions (e.g., after-hours GP clinic, GP cooperatives). PHCPs involved in the interventions were: GP (32%), NP (26%), nurses with expanded role (16%) and combinations of the PHCPs (42%). The "within ED" and "outside ED" interventions reported outcomes on patient flow and ED utilization, respectively.

For peer review only - http://bmjopen.bmj.com/site/about/guidelines.xhtml

Conclusions: We identified many interventions involving PHCPs that predominantly reported a positive impact on ED utilization/ patient flow metrics. Future research needs to focus on conducting well-designed RCTs and systematic reviews to evaluate the effectiveness of specific interventions involving PHCPs to critically appraise and summarize evidence on this topic.

For peer review only

For peer review only - http://bmjopen.bmj.com/site/about/guidelines.xhtml

Article Summary

Strengths and limitations of this study

- A major strength of our study is that we collaborated with twelve patient partners during the design, conduct and dissemination stages of this scoping review to refine the review question, identify patient-important review outcomes, develop search terms, grey literature search and knowledge dissemination.
- This comprehensive scoping review was conducted using JBI guidelines and an *a priori* registered protocol without any restrictions on publication dates, population, or study design
- A limitation of this scoping review is that we only included English language publications

For peer review only

For peer review only - http://bmjopen.bmj.com/site/about/guidelines.xhtml

INTRODUCTION

Emergency department (ED) overcrowding is an increasing global crisis,[1] leading to challenges in the delivery of timely healthcare to patients, which can result in adverse patient outcomes.[2,3] Overcrowding occurs when the demand for services exceeds the ability of the ED to provide quality care within acceptable time frames.[4] Studies have shown how ED crowding compromises patient safety and contributes to poor quality of care, such as delays in antibiotic treatment,[5] poor pain management for patients with severe pain,[6] increased patient mortality,[7] and staff frustration.[8,9]

The problem of ED overcrowding is complex and challenging, and models have been proposed to characterize it using input (e.g., health lines, patient volume, diversion strategies), throughput (e.g., provider availability, diagnostic testing, response to therapy), output (e.g., consultation times, ED boarding and lack of hospital beds), and system-wide (e.g., remuneration, pay-for performance, public reporting, accountability frameworks) factors.[10] One such input factor impacting ED overcrowding is the large volume of low acuity patient visits to the ED, which can stress the available resources in the ED, leading to decreased patient and staff satisfaction.[11,12] A lack of access to a primary care provider (PCP),[12-14] as well as an inability to see their PCP after-hours,[15] or to get an appointment within an appropriate time frame[3,15,16] can often force patients with low acuity conditions to turn to ED as their last resort.[17] Some have suggested that many of these presentations represent primary care sentinel conditions (e.g., asthma, hypertension, low back pain) which could be better managed through greater continuity and ongoing follow-up from primary care providers.[18] In many rural communities, EDs are often the only source of primary care.[4]

For peer review only - http://bmjopen.bmj.com/site/about/guidelines.xhtml

As such, it is crucial to identify effective interventions and strategies to mitigate the impact of lower acuity presentations on the ED and to improve patient outcomes. The main objective of this scoping review was to identify and summarize existing literature on the interventions and strategies involving Primary Healthcare Professionals (PHCPs: family physicians/general practitioners (GP), nurse practitioners (NP), or nurses with expanded role) to manage ED overcrowding.

For peer review only

For peer review only - http://bmjopen.bmj.com/site/about/guidelines.xhtml

METHODS

An *a priori* protocol was developed and posted on the Open Science Framework platform.[19] This scoping review was conducted using the methodologically rigorous Joanna Briggs Institute (JBI) Guideline for scoping reviews,[20] and the six-stage methodological framework outlined by Levac et al[21]. This scoping review adheres to the reporting guidelines of the Preferred Reporting Items for Systematic reviews and Meta-Analyses extension for scoping reviews (PRISMA-ScR).[22,23]

Population, Concept and Context (PCC)

Studies were included in the review if they investigated an intervention or strategy to manage ED overcrowding and ED patient flow and involved a primary healthcare professional (PHCP) including family physician/general practitioner (GP), nurse practitioner (NP) or nurse with increased authority. Editorials, commentaries, reviews, and historical articles were excluded from the review. For feasibility, only English language publications were included. Additional details regarding study eligibility criteria (Population, Concept and Context) are available in ***Appendix Table 1***.

Search methods for identifying relevant citations

A health librarian (TR) with experience conducting systematic literature searches designed a comprehensive search strategy for Medline (Ovid) to identify literature relevant to the objectives (***Appendix Table 2***). After feedback from the principal investigators and the patient partners, the Medline search was then peer reviewed by an independent librarian (JJ) using the Peer Review of Electronic Search Strategies (PRESS) checklist.[24] Once finalized, it was adapted for use in the following three additional electronic databases: EMBASE (Ovid), Cochrane Library (Wiley), and

For peer review only - http://bmjopen.bmj.com/site/about/guidelines.xhtml

CINAHL (EBSCO)). The databases were searched from their inception to January 2020 by an experienced librarian (NA). A search of several grey literature sources (***Appendix Table 3)*** was conducted to identify additional publications. Reference lists of all the included publications were searched for additional relevant studies. References were imported and managed in EndNote™ (Version X8, Thomson Reuters, New York, NY, USA).

Selection of sources of evidence

Two reviewers (NAY & (LC, YAY or RA)) independently reviewed and screened the titles and abstracts of all citations identified by our search strategy using standardized pilot-tested (n=10) screening forms. Citations identified as potentially relevant, or which the reviewers were unclear of its inclusion were eligible for full-text screening. Two reviewers (NAY & (LC or YAY)) independently reviewed full texts of all included studies and discrepancies were resolved via consensus or a third-party adjudicator (MJ).

Charting information, risk of bias assessment and synthesis of results

A standardized data extraction form was developed, and pilot tested (n=10) independently by reviewers in the team. As a part of data extraction, two reviewers (LC & (NAY, YAY or RA)) independently extracted data from the included studies. When required, the form was modified, using an iterative consensus-based process[25]. All disagreements among reviewers were resolved by consensus and checked for accuracy by a third reviewer (SK and/or MJ). Extracted data included: study identification, author(s), publication year, publication type, study design, country of origin, type of ED (urban versus rural; adult, pediatric, or mixed), type of intervention or strategy (within ED or outside ED), type of PHCP (GP, NP or nurse with increased authority), nature of

For peer review only - http://bmjopen.bmj.com/site/about/guidelines.xhtml

intervention or strategy (added a PHCP, or increased responsibility to a PHCP), and the nature of impact of interventions on ED related outcomes as reported by the study authors in the included studies (positive impact, negative impact, or no impact).

Consistent with established scoping review methodology,[25,26] we did not appraise the risk of bias of the included studies, nor did we summarize the data quantitatively (meta-analyses). Instead, we synthesized the findings using descriptive statistical analyses (e.g., frequencies and percentages) of the extracted variables, and reported using graphs and tables.

Patient and public involvement (Collaboration with stakeholders)

We collaborated with a diverse group of 13 patient partners (self-identified as Indigenous, Immigrant, White and/or living with disability) identified with the help of Strategy for Patient-Oriented Research (SPOR) Support for People and Patient-Oriented Research and Trials units (Manitoba, Alberta, and Quebec), during the design phase, grant application phase and in the conduct of this review. The patient engagement process has been reported according to the GRIPP2 checklist (short form).[27] During the design phase, 12 patient partners collaborated with the principle investigators (12 one-on-one interviews followed by discussion group to arrive at a consensus) to refine the review question to focus on interventions involving PHCPs, to finalize review inclusion criteria, and to select patient-important outcomes. During the grant application phase, they supported our grant application as knowledge users and provided letters of support. While conducting this scoping review, 3 patient partners collaborated with researchers in refining the search strategy, reviewing the included studies, identifying grey literature, interpreting study results (we shared knowledge of our study findings and integrated information obtained from them

For peer review only - http://bmjopen.bmj.com/site/about/guidelines.xhtml

in the reporting of the study findings and future directions), and in the knowledge dissemination process (conference presentation). Patient partners were compensated for their time.

Our team members (researchers and patient partners) from multiple provinces across Canada, and the partner organizations provided ongoing feedback. Three presentations were made to various members of the team during the conduct of this review by the principal investigator to discuss the extracted data, obtain feedback regarding the inclusion and classification of various identified interventions and strategies, as well as review outcomes of importance, and to discuss the future directions of the project (discussions regarding patient-important research priorities and review questions for future systematic reviews). Partnering organizations included Manitoba Medical Service Foundation (MMSF), SPOR SUPPORT units (Manitoba, Alberta, and Quebec), Emergency Medicine Research Group (EMeRG), and Primary and Integrated Healthcare Innovation network (PIHCI), and the knowledge users (Winnipeg Regional Health Authority, Emergency Strategic Clinical Network, Shared Health Manitoba, and Manitoba College of Family Physicians).

For peer review only - http://bmjopen.bmj.com/site/about/guidelines.xhtml

RESULTS

Our literature search identified 23,947 records from the initial database search, of which 268 studies (274 reports) met our inclusion criteria. A detailed description of the study selection process for this scoping review is depicted using the PRISMA study flow diagram for scoping reviews[22,28] (***Figure 1***).

Main Results

Study characteristics

The included studies (***Appendix Table 4***) were published between 1981 and 2020 with the majority of studies published in the last 10 years (2010-2020), showing an upward trend in the number of publications per year over time (1981-2020; ***Figure 2***). For details regarding the key characteristics of the included studies, see ***Table 1 & Appendix Table 5***. The included studies were mostly cohort (prospective and retrospective) studies (41.8%) from North America (58%) and Europe (28.4%) and published in peer-reviewed journals (76.9%). Nearly half of the included studies were conducted in USA (47%) and only 8.2% were randomized controlled trials. The ED location was most often urban (n = 137, 51%) and 16 studies (6%) reported an ED location that provided care to patients from rural and urban areas. Most of the studies did not specify the type of ED (n = 146, 54.5%); however, mixed (n = 79, 29.5%), adult (n = 19, 7%), or pediatric (n = 24, 9%) ED types were documented.

Interventions and Strategies involving PHCPs

For this review, interventions or strategies involving PHCPs were categorized as either "within ED" or "outside ED" interventions. A total of 139 studies (52%) reported "outside ED"

For peer review only - http://bmjopen.bmj.com/site/about/guidelines.xhtml

interventions or strategies, and 129 studies (48%) reported "within ED" interventions or strategies (***Appendix Table 6)***.

Several "within ED" interventions and strategies identified in this review. These interventions were grouped under four common themes as follows: (1) Area within ED staffed by PHCPs to manage lower acuity ED patients streamlined at triage[29]; (2) PHCPs located next to ED (sharing common triage with ED) to manage lower acuity ED patients streamed at triage as well as self-directed patients[30], (3) PHCPs located at ED triage[31] to manage lower acuity ED patients, and (4) PHCPs fully integrated within the ED to manage ED patients along with the ED team[32] (***Appendix Table 6)***.

The PHCP triage intervention involved patient management strategies such as, "see and treat" lower acuity patients,[31] diverting low acuity patients to adjacent/co-located primary care center or after-hours primary care center,[33] or the triage nurse was given increased authority to order diagnostic investigations or initiate a specified protocol.[34] A few "within ED" interventions involved low acuity patients streamlined at triage to a PHCP working alone within ED (for example rapid medical assessment units,[35] fast-track units,[36] or Emergency care access points[35]) for management. Other "within ED" interventions (non- triage) involved PHCPs working in conjunction with the emergency physician and the rest of the ED team in the interpretation of diagnostic imaging, management of lower acuity or in some cases higher acuity patients, and in discharge process.[32]

Studies employed several "outside ED" interventions or strategies. We identified four common themes across these interventions and grouped them under these following themes: (1) improving timely access to primary care, (2) integrating hospital and GP care, (3) providing financial support, and (4) implementing new clinics/services (***Appendix Table 6)***. Some examples

For peer review only - http://bmjopen.bmj.com/site/about/guidelines.xhtml

of the “outside ED” interventions or strategies were: increasing after-hours primary care,[37] free access to primary care for the uninsured,[38] adjacent or co-located primary care clinic for lower acuity patients,[39] introduction of a patient centered medical home that addresses primary care needs of patients,[40] implementation of GP co-operatives (out-of-hours primary health care in one centrally located practice),[41] urgent care collaborations between the GP and ED,[42] GP-led walk-in centers,[43] primary care provider blended fee for service,[44] hospital integrated general practice for emergency care services,[45] integrated emergency posts where care is provided by both ED and GPs,[46] rural health clinics,[47] and advanced access primary care with timely access.[48]

Involvement of primary healthcare providers

The type of PHCP involved in the interventions or strategies involving PHCPs were as follows: family physician (n = 85, 31.7%), nurse practitioner (n = 69, 25.7%), or nurse given increased authority (n = 43, 16%). There were also a number dyads reported, including family physician and nurse practitioner (n = 15, 5.6%), family physician and nurse (n = 2, 0.8%), or nurse practitioner and nurse (n = 8, 3%). In some studies, the intervention or strategy was described as primary care with no mention of the specific type of PHCP involved (n = 46, 17.2%). The majority of studies introduced or added a PHCP (n = 190, 70.1%) as a part of the intervention or strategy, and the rest of the studies increased the responsibility of the PHCPs as a part of the intervention or strategy (n = 59, 22%). In a few studies (n = 19, 7%) it was not clear if there was an addition or increased responsibility of a PHCP.

Reported Impact of PHCP Interventions and Strategies on ED outcomes

For peer review only - http://bmjopen.bmj.com/site/about/guidelines.xhtml

Several ED-related outcomes, and the impact (positive, negative or no impact) of the PHCP interventions and strategies on these ED outcomes were reported by the included studies. The majority of the included studies that reported "outside ED" interventions or strategies investigated the impact of these interventions on minimizing the ED utilization (ED visits), whereas studies reporting "within ED" interventions or strategies investigated the impact on ED patient flow metrics. The majority of the included studies reported ED outcomes such as ED visits, ED LOS (length of stay), leave without being seen (LWBS), patient satisfaction, patient safety, ED workup time, time to provider initial assessment, number of patients diverted to primary care, and ED cost savings (***Table 2***).

The reported impact (positive impact, negative impact, or no impact) of various PHCP-led interventions and strategies on various ED outcomes is reported in ***Table 2***. About 62% of the included studies reported a positive impact on ED utilization (i.e., decrease in lower acuity ED visits), whereas 28% reported no impact and 9.7% reported negative impact (increased ED utilization). Similarly, the majority of the included studies reported a positive impact on many important ED related outcomes such as ED length of stay (73.6%), leave without being seen (77.1%), patient satisfaction (62.1%), time to provider initial assessment (84.6%), ED workup time (62.5%), leave against medical advice (50%), patient safety (100%), and leave before completion of service (50%). The rest of the included studies reported either a negative impact or no impact for the above outcomes.

For peer review only - http://bmjopen.bmj.com/site/about/guidelines.xhtml

DISCUSSION

This scoping review identified 268 unique comparative studies that reported on various interventions or strategies involving PHCPs to manage ED overcrowding and ED patient flow (regardless of the geographic area or the type of healthcare system). Most of the included studies were journal articles of cohort design, published in North America, and were conducted in urban ED's. The interventions or strategies involving PHCPs were implemented either "within ED" or were "outside ED" within the community.

To the best of our knowledge, this is the first scoping review to describe various interventions or strategies involving PHCPs and their reported impact on ED outcomes. In this scoping review we identified and described various types of "within ED" (triage or non-triage) PHCP-led interventions from existing literature. In 2010, Carson et al[49] had described four models in which lower acuity patients could be managed within ED by PHCPs, as follows: (1) streaming ED patients at triage to an area within ED staffed by PHCPs, for management[49]; (2) PHCPs located next to ED (sharing triage with ED) for management of redirected low-acuity ED patients or self-directed patients[49]; (3) PHCPs located at triage who either "see and treat" or redirect low acuity patients to primary care[49]; (4) PHCPs fully integrated with the ED team supporting management of low acuity or higher acuity patients.[49] In this review, each of the "within ED" PHCP-led interventions reported by the included studies fit at least one of the four of the Carson's models. It is important to note that the PHCPs in these interventions were providing episodic care (episode related primary care) and not care continuity.

Although we are unaware of any previous scoping reviews on this topic, there are a few systematic reviews that have been previously published which found a positive impact of specific PHCP-led "within ED" interventions on ED patient flow metrics. In 2011, Rowe et al[50] reported

For peer review only - http://bmjopen.bmj.com/site/about/guidelines.xhtml

that triage nurse ordering (i.e. triage nurse given increased authority) had a positive impact on ED patient flow metrics Similarly, in another systematic review, Rowe et al[51] had reported a positive impact of triage liaison physicians on ED patient flow metrics. A more recent review reported that NPs practicing in the ED improved ED quality metrics and patient satisfaction [52], while another review investigating strategies to alleviate access block and overcrowding, suggested that co-locating primary care, fast-track and nurse practitioners within ED may have a positive impact.[53] Other reviews, however, have reported a limited impact of "within-ED" interventions on ED patient flow metrics. A previous Cochrane review,[54] which included only four studies, reported a very low certainty of evidence on the role of GP's and NP's within the ED (but not at triage) on ED flow metrics. In 2015, a narrative review[55] was published on the nurse practitioner role in the ED and reported that high quality research is required to demonstrate clinical and service effectiveness.

This review identified numerous types of "outside ED" interventions that have been employed across the literature. It is important to note that studies reporting "outside ED" interventions and strategies focused predominantly on reduction of ED utilization by patients and reported "ED visits" as an outcome in their study report. Although we are unaware of any scoping reviews on "outside ED" PHCP-led interventions, there have been a few relevant systematic reviews published to date on this topic. Ansell et al., 2017,[56] summarized interventions to reduce wait times for primary care appointments to mitigate ED utilization, and concluded that open access scheduling and patient-centered interventions may reduce wait times. Patients seeking ED care due to lack of timely primary care access (same-day or after-hours appointments) may contribute to ED overcrowding and in this context some of the "outside ED" interventions summarized in this scoping review may be beneficial in mitigating ED overcrowding.

For peer review only - http://bmjopen.bmj.com/site/about/guidelines.xhtml

The results of this scoping review found that the majority of studies reported a positive impact of PHCP-led interventions and strategies on ED overcrowding and patient flow metrics. Although what may work in one context may not work in another due to many factors (e.g., funding, political views, type of healthcare systems, etc.) that may play a role in determining the best intervention or strategy for a specific context. It is important to conduct systematic reviews to critically appraise and summarize evidence on the effectiveness of specific "within ED" or "outside ED" interventions involving PHCPs to mitigate ED overcrowding. In this scoping review we only identified eight relevant RCTs that reported PHCP-led interventions. Thus, it is important to conduct well designed RCTs evaluating the effectiveness of specific interventions involving PHCPs on ED outcomes to generate high quality evidence on this topic.

This review has numerous strengths. To limit selection bias, multiple steps and multiple independent reviewers reviewed the studies to make selections in an unbiased manner. One of the major strengths of this scoping review is that we engaged and collaborated with twelve patient partners from multiple provinces across Canada during the design stage to refine our review question and identify patient-important review outcomes, as well as during the conduct stage and knowledge dissemination stages. In addition, the JBI guidelines were used for conducting this scoping review along with an *a priori* registered protocol. A comprehensive search strategy was developed by an experienced health librarian and peer-reviewed by a second information specialist using PRESS guidelines along with search for grey literature sources, to identify relevant studies to answer our review question. In addition, the search strategy was reviewed by our patient partners who collaborated on the project. We did not have any restrictions on publication dates, population, or study design (with exception of narrative reviews, editorials, commentaries, and historical articles).

For peer review only

For peer review only - http://bmjopen.bmj.com/site/about/guidelines.xhtml

There are some important limitations with this review to be considered. In our review, publication bias was only partially addressed. Despite using a comprehensive and exhausting search strategy, we made a pragmatic decision to only include English language publications. Consequently, it is possible that some of the studies from countries where English is not the first language may have been missed. Our review focused on the interventions and strategies that involved only PHCPs such as family physicians, nurse practitioners and nurses with increased authority. Studies that involved ED physicians or specialists were not considered in this review for feasibility. We had also made an *a priori* decision, for feasibility, to not include studies that had exclusively investigated the role of physician assistants, but if a study reported them to be a part of a team that consisted of PHCPs, then we included those studies in our scoping review. We included studies that had involved any one of the included PHCPs individually or as a part of a team that had other professionals, and thus in these studies the impact of intervention cannot be solely ascribed to a specific PHCP.

Conclusions

This scoping review provides an up-to-date overview of various interventions and strategies involving PHCPs reported in the literature and summarizes the nature of the impact these interventions were reported to have on ED overcrowding and ED patient flow metrics. Mitigation of ED overcrowding is a multidimensional process. This scoping review identified and reported the innovative ways in which PHCPs may be engaged in patient care and help to mitigate ED overcrowding. The summarized impact of the interventions and strategies need to be approached with caution as the geographic area in which these studies were conducted, and the type of healthcare systems vary widely across the included studies and potentially influence how these

For peer review only - http://bmjopen.bmj.com/site/about/guidelines.xhtml

results may be interpreted. This scoping review could be a timely contribution to the growing problem of ED overcrowding. Moving forward, it is important to conduct systematic reviews to summarize evidence on effectiveness of specific "within ED" or "outside ED" interventions or strategies involving PHCPs to mitigate ED overcrowding and improve patient flow metrics. It is also important to engage and obtain diverse patient and caregiver perspectives throughout the systematic review process to identify patient-important priorities and key patient-important outcomes. In addition, it is also important for researchers to conduct well-designed RCTs to generate high quality evidence on this topic.

For peer review only

For peer review only - http://bmjopen.bmj.com/site/about/guidelines.xhtml

Acknowledgements: We are very grateful to the Canadian Institutes of Health Research (CIHR) (NKS 158643), Manitoba Medical Services Foundation (# 8-2018-06) and Winnipeg Foundation (# 8-2018-06) for providing financial support for this project. Dr. Rowe's research was supported by a Scientific Director's Grant (SOP 168483) from CIHR. Dr. Tricco's research is funded by a Tier 2 Canada Research Chair in Knowledge Synthesis. We thank the Emergency Medicine Research Group (EMeRG) in the Department of Emergency Medicine at the University of Alberta for in-kind resources. We are also very grateful to all the patient partners who collaborated with us in the design, conduct and dissemination stages of this project. We are thankful to Mr. Frank Krupka for the kind support of this project during his time as the Executive Director of CHI. We are very grateful to Dr. Cristina Villa-Roel for the kind support of this project. We thank Ms. Tamara Rader, Ms. Christine Nielson, and Ms. Janet Joyce for help with the search strategy for this study.

Funding: Research grant from Canadian Institutes of Health Research (NKS 158643), Manitoba Medical Services Foundation (# 8-2018-06) and Winnipeg Foundation (# 8-2018-06).

Competing interests: None declared.

Data sharing statement: Not applicable

Author contributions: MJ, AMAS, TB and MH substantially contributed to the design and conception of the study. MJ drafted the manuscript with input from all co-authors. MJ, AMAS and RR were involved in the data analysis. NA was involved in developing search strategy. LC, RA, YAY, SK, and NAY were involved in study selection process and in data extraction. MD, SB, JM, PT, BHR, AC, WS provided content expertise in emergency medicine. RS, GH, TB, and JE provided content expertise in family medicine. CS provided content expertise in patient

For peer review only - http://bmjopen.bmj.com/site/about/guidelines.xhtml

engagement. ACT, RZ and AMAS provided content expertise in scoping review methodology. All study authors provided critical feedback and approved the final manuscript.

Abbreviations:

ATS: Australian triage scale

CINAHL: Cumulative Index to Nursing and Allied Health Literature

CTAS: Canadian Triage and Acuity Scale

CBA: Controlled before and after

ED: Emergency department

EMBASE: Excerpta Medica Database

ESI: Emergency Severity Index

GP: General practitioner

JBI: Joanna Briggs Institute

LAMA: Leaving against medical advice

LOS: Length of stay

LWBS: Leaving without being seen

NP: Nurse practitioner

PHCP: Primary healthcare provider

PRESS: Peer Review of Electronic Search Strategies

For peer review only - http://bmjopen.bmj.com/site/about/guidelines.xhtml

PRISMA: Preferred Reporting Items for Systematic reviews and Meta-Analyses

ScR: Scoping Review

For peer review only

For peer review only - http://bmjopen.bmj.com/site/about/guidelines.xhtml

References:

1. Holden RJ. Lean Thinking in emergency departments: a critical review. *Annals of emergency medicine.* 2011;57(3):265-278.
2. Thrasher C, Purc-Stephenson RJ. Integrating nurse practitioners into Canadian emergency departments: a qualitative study of barriers and recommendations. *Cjem.* 2007;9(4):275-281.
3. Canadian Association of Emergency Physicians and National Emergency Nurses Affiliation. Joint position statement on emergency department overcrowding. Canadian Journal of Emergency Medecine. 2001;3(2):82-84. http://www.cjem-online.ca/v3/n2/p82.
4. Guo B, Harstall C. Strategies to reduce emergency department overcrowding. Health Technology Assesment report #38. *Alberta Heritage Foundation for Medical Research.* 2006.
5. Fee C, Weber EJ, Maak CA, Bacchetti P. Effect of emergency department crowding on time to antibiotics in patients admitted with community-acquired pneumonia. *Annals of emergency medicine.* 2007;50(5):501-509, 509 e501.
6. Pines JM, Hollander JE. Emergency department crowding is associated with poor care for patients with severe pain. *Annals of emergency medicine.* 2008;51(1):1-5.
7. Guttmann A, Schull MJ, Vermeulen MJ, Stukel TA. Association between waiting times and short term mortality and hospital admission after departure from emergency department: population based cohort study from Ontario, Canada. *Bmj.* 2011;342:d2983.
8. Fatovich DM. Effect of ambulance diversion on patient mortality: how access block can save your life. *The Medical journal of Australia.* 2005;183(11-12):672-673.
9. Fatovich DM, Nagree Y, Sprivulis P. Access block causes emergency department overcrowding and ambulance diversion in Perth, Western Australia. *Emergency medicine journal : EMJ.* 2005;22(5):351-354.
10. Asplin BR, Magid DJ, Rhodes KV, Solberg LI, Lurie N, Camargo CA, Jr. A conceptual model of emergency department crowding. *Annals of emergency medicine.* 2003;42(2):173-180.
11. Position Statement on Emergency Medicine Definitions From the Canadian Association of Emergency Physicians. *Canadian Association of Emergency Physicians 2016.*
12. Rocovich C, Patel T. Emergency department visits: Why adults choose the emergency room over a primary care physician visit during regular office hours? *World J Emerg Med.* 2012;3(2):91-97.
13. Doetzel CM, Rankin JA, Then KL. Nurse Practitioners in the Emergency Department: Barriers and Facilitators for Role Implementation. *Advanced emergency nursing journal.* 2016;38(1):43-55.
14. Statistics Canada. Access to a regular medical doctor, 2013. Available from: http://www.statcan.gc.ca/pub/82-625-x/2014001/article/14013-eng.htm.
15. Wong WB, Edgar G, Liddy C, Vaillancourt C. Can after-hours family medicine clinics represent an alternative to emergency departments? Survey of ambulatory patients seeking after-hours care. *Canadian family physician Medecin de famille canadien.* 2009;55(11):1106-1107 e1104.
16. Brousseau DC, Bergholte J, Gorelick MH. The effect of prior interactions with a primary care provider on nonurgent pediatric emergency department use. *Archives of pediatrics & adolescent medicine.* 2004;158(1):78-82.
17. Liggins K. Inappropriate attendance at accident and emergency departments: a literature review. *Journal of advanced nursing.* 1993;18(7):1141-1145.
18. Van den Heede K, Van de Voorde C. Interventions to reduce emergency department utilisation: A review of reviews. *Health policy.* 2016;120(12):1337-1349.
19. Primary Care Interventions and Strategies to Mitigate Overcrowding of Emergency Departments: A Scoping review protocol. Open Science Framework (OSF) pre-registration - https://osf.io/z2pgc. 2019.

For peer review only - http://bmjopen.bmj.com/site/about/guidelines.xhtml

20. The Joanna Briggs Institute Reviewers' Manual. 2015. Methodology for JBI Scoping Reviews. https://joannabriggs.org/assets/docs/sumari/Reviewers-Manual_Methodology-for-JBI-Scoping-Reviews_2015_v2.pdf
21. Levac D, Colquhoun H, O'Brien KK. Scoping studies: advancing the methodology. *Implementation science : IS.* 2010;5:69.
22. Tricco A, Straus S, Moher D. Preferred Reporting Items for Systematic Reviews and Meta-Analysis: extension for Scoping Reviews (PRISMA-ScR). http://www.equator-network.org/wp-content/uploads/2009/02/Executive-summary_ScR_Dec-9.pdf.
23. McGowan J, Straus S, Moher D, et al. Reporting scoping reviews-PRISMA ScR extension. *Journal of clinical epidemiology.* 2020.
24. McGowan J, Sampson M, Salzwedel DM, Cogo E, Foerster V, Lefebvre C. PRESS Peer Review of Electronic Search Strategies: 2015 Guideline Statement. *Journal of clinical epidemiology.* 2016;75:40-46.
25. Tricco AC, Lillie E, Zarin W, et al. PRISMA Extension for Scoping Reviews (PRISMA-ScR): Checklist and Explanation. *Ann Intern Med.* 2018;169(7):467-473.
26. Peters MDJGC, McInerney P, Baldini Soares C, Khalil H, Parker D. Chapter 11: scoping reviews. In: Aromataris E, Munn Z, editors. Joanna Briggs Institute Reviewer's Manual: The Joanna Briggs Institute, 2017. https://wiki.joannabriggs.org/display/MANUAL/Chapter+11%3A+Scoping+reviews. Accessed 15 Feb 2020. .
27. Staniszewska S, Brett J, Simera I, et al. GRIPP2 reporting checklists: tools to improve reporting of patient and public involvement in research. *Bmj.* 2017;358:j3453.
28. Moher D, Liberati A, Tetzlaff J, Altman DG, Group P. Preferred reporting items for systematic reviews and meta-analyses: the PRISMA statement. *PLoS medicine.* 2009;6(7):e1000097.
29. Rothwell S, McIltrot K, Khouri-Stevens Z. Addressing Emergency Department Issues Using Advanced Practice in Saudi Arabia. *Journal for Nurse Practitioners.* 2018;14(2):e41-e44.
30. Salisbury C, Hollinghurst S, Montgomery A, et al. The impact of co-located NHS walk-in centres on emergency departments. *Emergency Medicine Journal.* 2007;24(4):265-269.
31. Lutze M, Ratchford A, Fry M. A review of the Transitional Emergency Nurse Practitioner. *Australasian Emergency Nursing Journal.* 2011;14(4):226-231.
32. Ducharme J, Alder RJ, Pelletier C, Murray D, Tepper J. The impact on patient flow after the integration of nurse practitioners and physician assistants in 6 Ontario emergency departments. *CJEM Canadian Journal of Emergency Medical Care.* 2009;11(5):455-461.
33. Anantharaman V. Impact of health care system interventions on emergency department utilization and overcrowding in Singapore. *International Journal of Emergency Medicine.* 2008;1(1):11-20.
34. Lindley-Jones M, Finlayson BJ. Triage nurse requested x rays--are they worthwhile? *J Accid Emerg Med.* 2000;17(2):103-107.
35. Platter MEM, Kurvers RAJ, Janssen L, Verweij MMJ, Barten DG. The impact of an emergency care access point on pediatric attendances at the emergency department: An observational study. *The American journal of emergency medicine.* 2019.
36. Considine J, Kropman M, Kelly E, Winter C. Effect of emergency department fast track on emergency department length of stay: a case-control study. *Emergency Medicine Journal.* 2008;25(12):815-819.
37. Sterner SE, Coco T, Monroe KW, King WD, Losek JD. A new after-hours clinic model provides cost-saving, faster care compared with a pediatric emergency department. *Pediatric Emergency Care.* 2012;28(11):1162-1165.

For peer review only - http://bmjopen.bmj.com/site/about/guidelines.xhtml

38. Mackinney T, Visotcky AM, Tarima S, Whittle J. Does providing care for uninsured patients decrease emergency room visits and hospitalizations? *Journal of Primary Care & Community Health.* 2013;4(2):135-142.
39. Sharma A, Inder B. Impact of co-located general practitioner (GP) clinics and patient choice on duration of wait in the emergency department. *Emergency Medicine Journal.* 2011;28(8):658-661.
40. Nichols DE, Haber SG, Romaire MA, Wensky SG, Multi-Payer Advanced Primary Care Practice Evaluation T. Changes in Utilization and Expenditures for Medicare Beneficiaries in Patient-centered Medical Homes: Findings From the Multi-Payer Advanced Primary Care Practice Demonstration. *Medical care.* 2018;56(9):775-783.
41. Van Uden CJT, Winkens RAG, Wesseling GJ, Crebolder HFJM, Van Schayck CP. Use of out of hours services: A comparison between two organisations. *Emergency Medicine Journal.* 2003;20(2):184-187.
42. van Gils-van Rooij ESJ, Meijboom BR, Broekman SM, Yzermans CJ, de Bakker DH. Is patient flow more efficient in Urgent Care Collaborations? *European Journal of Emergency Medicine.* 2018;25(1):58-64.
43. Arain M, Nicholl J, Campbell M. GP-led walk-in centre in the UK: Another way for urgent healthcare provision. *Critical Care.* 2013;2):S97.
44. To T, Guan J, Zhu J, et al. Quality of asthma care under different primary care models in Canada: a population-based study. *BMC family practice.* 2015;16:19.
45. Chmiel C, Wang M, Sidler P, Eichler K, Rosemann T, Senn O. Implementation of a hospital-integrated general practice--a successful way to reduce the burden of inappropriate emergency-department use. *Swiss Medical Weekly.* 2016;146:w14284.
46. Kool RB, Homberg DJ, Kamphuis HC. Towards integration of general practitioner posts and accident and emergency departments: a case study of two integrated emergency posts in the Netherlands. *BMC health services research.* 2008;8:225.
47. Wright B, Potter AJ, Trivedi AN, Mueller KJ. The Relationship Between Rural Health Clinic Use and Potentially Preventable Hospitalizations and Emergency Department Visits Among Medicare Beneficiaries. *The Journal of rural health : official journal of the American Rural Health Association and the National Rural Health Care Association.* 2018;34(4):423-430.
48. Kobewka DM, Kunkel E, Hsu A, Talarico R, Tanuseputro P. Physician Availability in Long-term Care and Resident Hospital Transfer: A Retrospective Cohort Study. *Journal of the American Medical Directors Association.* 2019.
49. Carson D, Clay H, Stern R. Primary Care and Emergency Departments. *Primary Care Foundation* 2010.
50. Rowe BH, Villa-Roel C, Guo X, et al. The role of triage nurse ordering on mitigating overcrowding in emergency departments: a systematic review. *Academic emergency medicine : official journal of the Society for Academic Emergency Medicine.* 2011;18(12):1349-1357.
51. Rowe BH, Guo X, Villa-Roel C, et al. The role of triage liaison physicians on mitigating overcrowding in emergency departments: a systematic review. *Academic emergency medicine : official journal of the Society for Academic Emergency Medicine.* 2011;18(2):111-120.
52. Fowler LH, Landry J, Nunn MF. Nurse Practitioners Improving Emergency Department Quality and Patient Outcomes. *Crit Care Nurs Clin North Am.* 2019;31(2):237-247.
53. Chan SS, Cheung NK, Graham CA, Rainer TH. Strategies and solutions to alleviate access block and overcrowding in emergency departments. *Hong Kong Med J.* 2015;21(4):345-352.
54. Goncalves-Bradley D, Khangura JK, Flodgren G, Perera R, Rowe BH, Shepperd S. Primary care professionals providing non-urgent care in hospital emergency departments. *The Cochrane database of systematic reviews.* 2018;2:CD002097.

For peer review only - http://bmjopen.bmj.com/site/about/guidelines.xhtml

55. Jennings N, Clifford S, Fox AR, O'Connell J, Gardner G. The impact of nurse practitioner services on cost, quality of care, satisfaction and waiting times in the emergency department: a systematic review. *International journal of nursing studies.* 2015;52(1):421-435.
56. Ansell D, Crispo JAG, Simard B, Bjerre LM. Interventions to reduce wait times for primary care appointments: a systematic review. *BMC health services research.* 2017;17(1):295.

For peer review only

For peer review only - http://bmjopen.bmj.com/site/about/guidelines.xhtml

Table 1: Characteristics of included studies in a scoping review of primary healthcare professional interventions to address emergency department overcrowding.

**Characteristics**		**n (%) (N = 268)**
**Publication type**	Journal article	206 (76.9)
	Abstract	62 (23.1)
**Continents**	North America	156 (58.2)
	Europe	76 (28.4)
	Oceania	22 (8.2)
	Asia	9 (3.4)
	Middle east	3 (1.0)
	South America	1 (0.4)
	Not reported	1 (0.4)
**Type of ED (rural versus urban)**	Rural ED	5 (2.0)
	Urban ED	137 (51.0)
	Both	16 (6.0)
	Not reported	110 (41.0)
**Type of ED (adult versus other)**	Adult ED	19 (7.0)
	Pediatric ED	24 (9.0)
	Mixed ED	79 (29.5)
	Not reported	146 (54.5)
**Type of intervention or strategy**	Within ED	129 (48.0)
	Outside ED	139 (52.0)
**Type of primary healthcare professional**	Family Physician (FP)	85 (31.7)
	Nurse practitioner (NP)	69 (25.7)
	Nurse with expanded role	43 (16.0)
	FP & NP	15 (5.6)
	FP & Nurse	2 (0.8)
	NP & Nurse	8 (3.0)
	Not reported	46 (17.2)
**Nature of intervention or strategy**	Added a primary healthcare professional	190 (71.0)
	Increased responsibility	59 (22.0)
	Not reported	19 (7.0)
**Study designs**	Randomized clinical trial	22 (8.2)
	Non-randomized trial	9 (3.4)
	Cohort study (prospective or retrospective)	112 (41.8)
	Case-control study	4 (1.5)
	Cross-sectional study	27 (10.0)
	Pre-post study	76 (28.4)
	Interrupted time series	14 (5.2)
	Mixed-methods study	4 (1.5)

For peer review only - http://bmjopen.bmj.com/site/about/guidelines.xhtml

Table 2: Reported impact of interventions involving primary healthcare professionals to address emergency department overcrowding

Reported Outcomes	Positive Impact n (%)	No Impact n (%)	Negative Impact n (%)
**ED utilization (ED visits)**	109 (62.0%)	49 (28.0%)	17 (9.7%)
**Time to initial provider assessment**	22 (84.6%)	2 (7.7%)	2 (7.7%)
**Patient satisfaction**	36 (62.1%)	20 (34.5%)	2 (3.4%)
**Patient safety**	4 (100.0%)	0 (0.0%)	0 (0.0%)
**ED Length of stay (ED LOS)**	67 (73.6%)	21 (23.1%)	3 (3.3%)
**Leaving without being seen (LWBS)**	27 (77.1%)	7 (20.0%)	1 (2.9%)
**Time from being seen in the ED to discharge**	4 (66.7%)	1 (16.7%)	1 (16.7%)
**ED workup time**	5 (62.5%)	2 (25.0%)	1 (12.5%)
**Leave against medical advice (LAMA)**	1 (50.0%)	0 (0.0%)	1 (50.0%)
**Left before completion of service (LBCS)**	1 (50.0%)	1 (50.0%)	0 (0.0%)
**Time from admission to first nursing activity**	1 (100.0%)	0 (0.0%)	0 (0.0%)
**Time from admission to communication of the diagnosis**	1 (100.0%)	0 (0.0%)	0 (0.0%)
**Number of patients diverted to primary care**	14 (70.0%)	6 (30.0%)	0 (0.0%)
**Bed-to-provider time**	1 (100.0%)	0 (0.0%)	0 (0.0%)
**ED wait time**	22 (68.8%)	9 (28.1%)	1 (3.1%)
**Volume of patients visiting ED**	4 (66.7%)	1 (16.7%)	1 (16.7%)
**ED wait time (qualitative)**	7 (77.8%)	0 (0.0%)	2 (22.2%)
**Decrease in non-urgent/ low-acuity ED visits**	0 (0.0%)	1 (100.0%)	0 (0.0%)
**Annual local budget for treatment of emergency patients**	1 (50.0%)	1 (50.0%)	0 (0.0%)
**Triage time**	1 (100.0%)	0 (0.0%)	0 (0.0%)
**Cost saving**	8 (100.0%)	0 (0.0%)	0 (0.0%)
**Length of first consultation**	0 (0.0%)	0 (0.0%)	1 (100.0%)
**Wait time to receive other services**	0 (0.0%)	0 (0.0%)	1 (100.0%)

For peer review only - http://bmjopen.bmj.com/site/about/guidelines.xhtml

Figure legends

Figure 1: PRISMA study flow chart for scoping review of primary healthcare professional interventions to address emergency department overcrowding.

Figure 2: Number of publications per year involving primary healthcare professional interventions to address emergency department overcrowding.

For peer review only

For peer review only - http://bmjopen.bmj.com/site/about/guidelines.xhtml

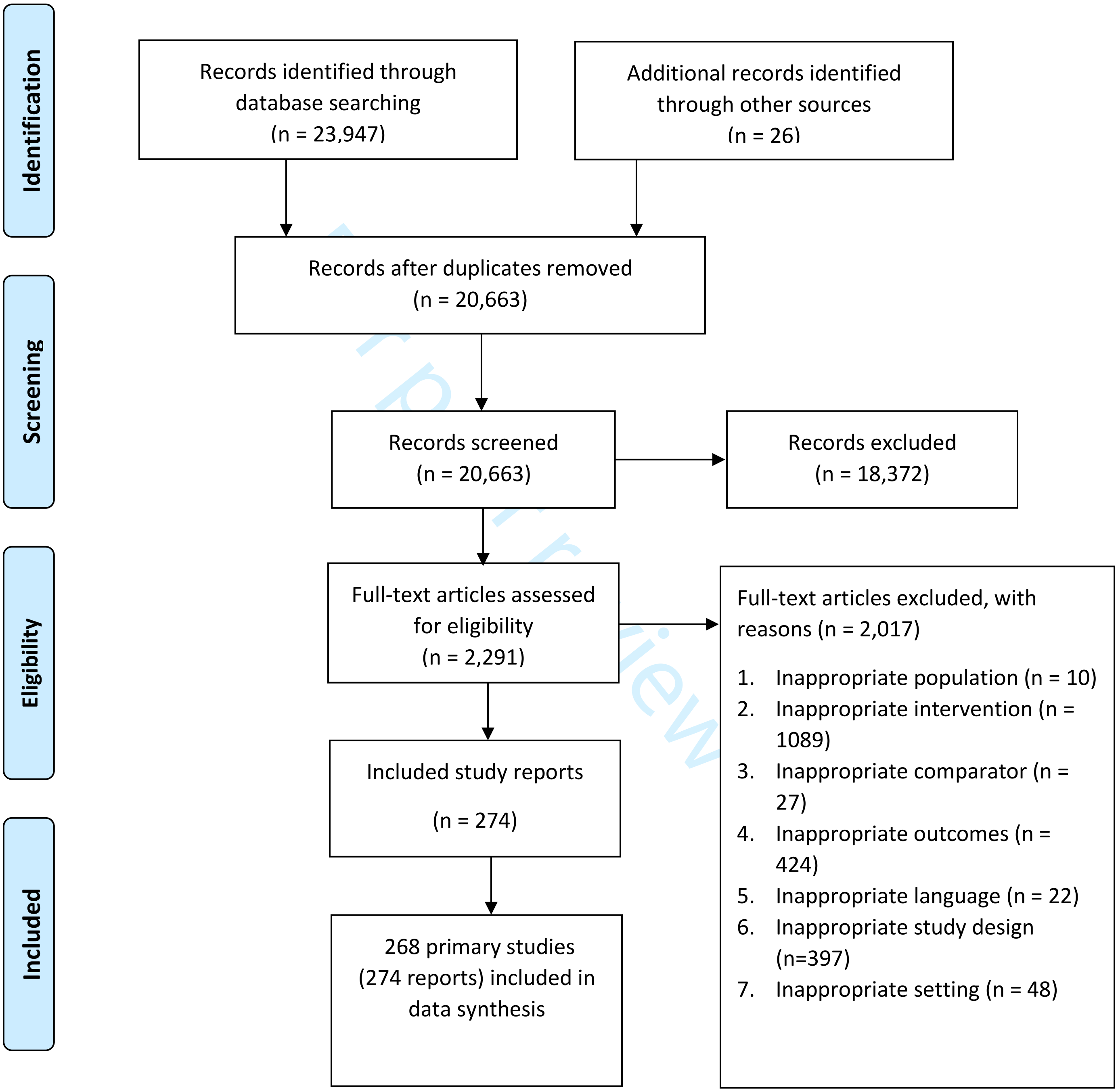

Identification
Screening
Eligibility
Included
Records identified through database searching (n = 23,947)
Additional records identified through other sources (n = 26)
Records after duplicates removed (n = 20,663)
Records screened (n = 20,663)
Records excluded (n = 18,372)
Full-text articles assessed for eligibility (n = 2,291)
Full-text articles excluded, with reasons (n = 2,017)
1. Inappropriate population (n = 10)
2. Inappropriate intervention (n = 1089)
3. Inappropriate comparator (n = 27)
4. Inappropriate outcomes (n = 424)
5. Inappropriate language (n = 22)
6. Inappropriate study design (n=397)
7. Inappropriate setting (n = 48)
Included study reports (n = 274)
268 primary studies (274 reports) included in data synthesis

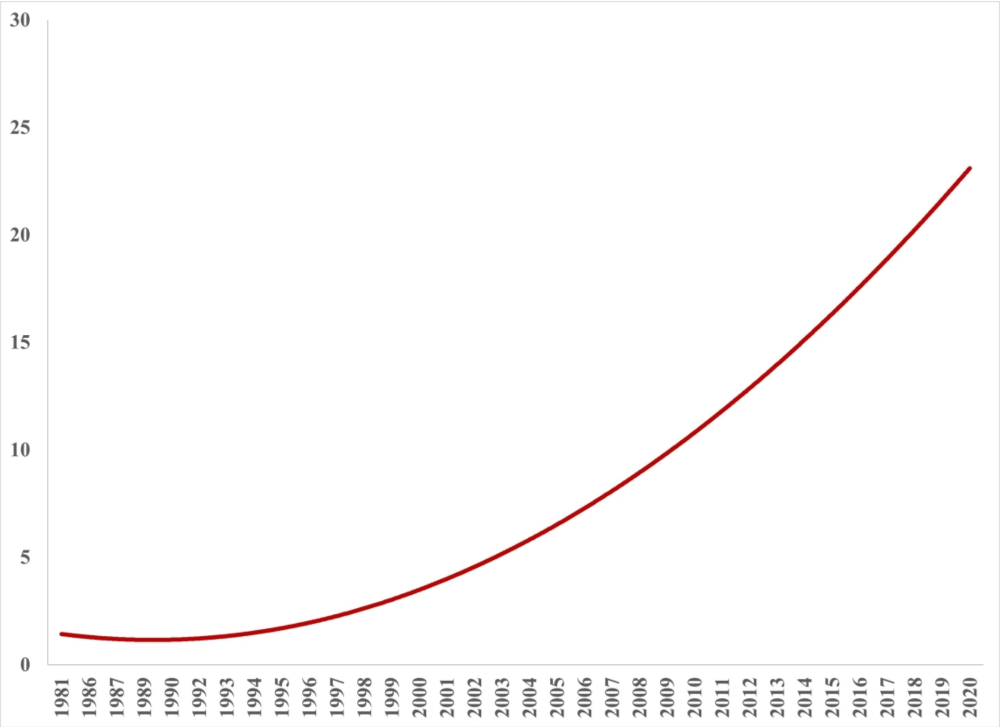

Number of publications per year involving primary healthcare professional interventions to address emergency department overcrowding.

240x174mm (300 x 300 DPI)

Appendix Table 1: Population, Concept and Context

**Population, Concept and Context**	
**Population**	Any patient population (children and adults of any age) visiting ED
**Intervention**	Any intervention or strategy involving primary healthcare professionals (family physician/general practitioner (GP), nurse practitioner (NP) & nurse with expanded authority) that was implemented to reduce overcrowding of ED
**Outcomes**	All ED outcomes that reflect the impact of interventions and strategies involving primary healthcare professionals (e.g., proportion of patients that leave ED without being seen (LWBS), ED length of stay (LOS), patient satisfaction, mean/median patient wait times in ED, percentage of patients with reduced wait times in ED, time to initial physician assessment, and volume of patients visiting ED)
**Study designs**	All study designs will be included except the following publication types: narrative reviews, editorials, commentaries, and historical articles.
**Publication status**	Only data from published studies will be included for feasibility.
**Language limits**	Only English language publications will be included for feasibility.
**Years considered**	No restriction on publication dates

For peer review only

Appendix Table 2: Medline Search Strategy

Database: Ovid MEDLINE(R) and Epub Ahead of Print, In-Process & Other Non-Indexed Citations and Daily <1946 to January 10, 2020>
Search Strategy:
----
1 exp primary health care/ (140156)
2 physicians, family/ (15853)
3 family practice/ (64029)
4 Physicians, Primary Care/ (2703)
5 general practice/ (11719)
6 general practitioners/ (6381)
7 (primary adj2 (care or health*)).ti,ab,kf. (129329)
8 ((general or family) adj (practice* or practitioner*)).ti,ab,kf. (84810)
9 (GP or GPs).ti,ab,kf. (51935)
10 nurse practitioners/ (16770)
11 primary care nursing/ (392)
12 family nursing/ (1349)
13 community mental health services/ (17905)
14 ((family or community or primary or ambulatory or triage) adj2 (medic* or doctor* or physician* or health* or nurs*)).ti,ab,kf. (68438)
15 Ambulatory Care/ (40524)
16 (ambulatory adj2 care).ti,ab,kf. (11413)
17 Health Services, Indigenous/ (2817)
18 Cultural Competency/ (4632)
19 Culturally Competent Care/ (830)
20 Medicine, Traditional/ (10299)
21 (trauma adj inform*).ti,ab,kf. (617)
22 (aborigin* or indigenous or native).ti,ab,kf. (225451)
23 ((after or out) adj2 hour*).ti,ab,kf. (137459)
24 or/1-23 (917850)
25 exp Emergency Service, Hospital/ (67418)
26 Emergency Medical Services/ (39232)
27 emergency treatment/ (10025)
28 Trauma centers/ (9210)
29 Triage/ (10240)
30 ((emergency or emergent or urgent) adj2 (care or healthcare or department* or unit or units or room* or treatment* or ward or service)).ti,ab,kf. (121497)
31 ("accident and emergency" or "accident & emergency" or ED or EDs or ER or A&E).ti,ab,kf. (162648)
32 (triage adj2 (centre or centres or center or centers or department? or unit or units)).ti,ab,kf. (538)
33 (emergency adj2 (care or healthcare or department? or unit or units or room? or treatment? or care or visit? or utilization or admit or admission?)).ti,ab,kf. (112731)
34 ("accident and emergency" or "accident & emergency" or emergency service?).ti,ab,kf. (10865)

For peer review only

35 (trauma adj2 (centre or centres or center or centers or department? or unit or units)).ti,ab,kf. (15573)
36 (triage adj2 (centre or centres or center or centers or department? or unit or units)).ti,ab,kf. (538)
37 (emergency adj2 (visit? or care or admit or admission?)).ti,ab,kf. (26760)
38 (urgent adj2 (care or healthcare or health care)).ti,ab,kf. (2099)
39 ((semiurgent or semi-urgent or nonemergen$ or non-emergen$) adj2 (treatment? or care or visit?)).ti,ab,kf. (289)
40 ((emergency or non-emergency or nonemergency or urgent or non-urgent or nonurgent or semi-urgent or semiurgent) adj2 patient?).ti,ab,kf. (11636)
41 or/25-40 (367776)
42 organizational efficiency/ (20744)
43 workflow/ (3295)
44 Waiting lists/ (10724)
45 ((wait or waiting) adj2 (time or times or list or lists)).ti. (3351)
46 ((wait or waiting or throughput or service or treatment) adj2 (time or times or list or lists) adj10 (reduce? or reduction or eliminat$ or lower or fewer or intervention or policy or policies or reform$ or effectiveness or impact or improv$ or organi?ational$ or quality or save or saving)).ab. (3119)
47 ((decrease or reduce or streamline or less or minimize or shorten or eliminate or cut or enhance or facilitate or speed or better or accelerate or optimize or reform or delay or change or faster or impact$ or assess$ or eliminat$ or improv$ or lower$ or reduc$) adj3 patient? wait$).ti,ab,kf. (303)
48 CROWDING/ (2930)
49 crowd$.ti,ab,kf. (16513)
50 congest$.ti,ab,kf. (61747)
51 overcrowd$.ti,ab,kf. (3425)
52 gridlock$.ti,ab,kf. (180)
53 queue$.ti,ab,kf. (1011)
54 overload$.ti,ab. (39413)
55 "access block$".ti,ab,kf. (166)
56 (throughput or through-put).ti,ab,kf. (87262)
57 warehous$.ti,ab,kf. (2303)
58 ("left without being seen" or "leave$ without being seen" or lwbs).ti,ab,kf. (284)
59 (patient adj2 elop$).ti,ab,kf. (16)
60 (ambulance$ adj2 diver$).ti,ab,kf. (194)
61 (ambulance$ adj2 redirect$).ti,ab,kf. (3)
62 "fast track$".ti,ab,kf. (3500)
63 delay$.ti,ab,kf. (428757)
64 ("patient flow$" or "flow of patient$").ti,ab,kf. (4939)
65 defer$.ti,ab,kf. (23198)
66 (over* adj3 (capacit$ or occupanc$)).ti,ab,kf. (4603)
67 (lama or (leave$ adj4 ("medical advice" or treatment$)) or (left adj4 ("medical advice" or treatment$))).ti,ab,kf. (8393)
68 ((hallway or corridor) adj2 (care or medicine)).ti,ab,kf. (6)
69 or/42-68 (776721)
70 24 and 41 and 69 (3799)

For peer review only

Appendix Table 3: Grey literature sources

Grey literature sources
BMJ Open Quality (https://bmjopenquality.bmj.com) and a Google Custom Search of the following websites:<br>Canadian Foundation for Healthcare Improvement (www.cfhi-fcass.ca), Institute for Healthcare Improvement (www.ihi.org), Agency for Healthcare Research and Quality (www.ahrq.gov), NHS Improvement (https://improvement.nhs.uk), International Society for Quality in Health Care (www.isqua.org), Health Quality Ontario (www.hqontario.ca), Saskatchewan Health Quality Council (https://hqc.sk.ca), Health Quality Council of Alberta (www.hqca.ca), BC Patient Safety & Quality Council (https://bcpsqc.ca), Australian Commission on Safety and Quality in Health Care (www.safetyandquality.gov.au), and Health Quality & Safety Commission New Zealand (www.hqsc.govt.nz).

For peer review only

Appendix 4: List of studies included in the scoping review

1. Adam H, Tamim H, Altamimi S, et al. Effect of triage nurse ordered distal extremity X-rays on emergency department length of stay: A randomized controlled trial. *Academic Emergency Medicine.* 2014;21(5 SUPPL. 1):S195.
2. Al Abri FH, Muliira JK, Al Awaisi H. Effect of triage nurse-led application of the Ottawa Ankle Rules on number of radiographic tests and length of stay in selected emergency departments in Oman. *Japan journal of nursing science : JJNS.* 2020;17(1):e12270.
3. Al Kadhi O, Manley K, Natarajan M, et al. A renal colic fast track pathway to improve waiting times and outcomes for patients presenting to the emergency department. *Open access emergency medicine : OAEM.* 2017;9:53-55.
4. Alcusky M, Singer D, Keith SW, et al. Evaluation of Care Processes and Health Care Utilization in Newly Implemented Medical Homes in Italy: A Population-Based Cross-sectional Study. *Am J Med Qual.* 2019:1062860619860590.
5. Almeida A, Vales J. The impact of primary health care reform on hospital emergency department overcrowding: Evidence from the Portuguese reform. *The International journal of health planning and management.* 2019.
6. Anantharaman V. Impact of health care system interventions on emergency department utilization and overcrowding in Singapore. *International Journal of Emergency Medicine.* 2008;1(1):11-20.
7. Ankeny A, Isenberger K, Westgard B, Stuck L, Wewerka S. Association of missed outpatient appointments and emergency department visits and inpatient admissions. *Annals of Emergency Medicine.* 2014;1):S87-S88.
8. Anonymous. Mobile unit helps ED cut LWBS in half. *ED management : the monthly update on emergency department management.* 2008;20(3):31-32.
9. Arain M, Nicholl J, Campbell M. GP-led walk-in centre in the UK: Another way for urgent healthcare provision. *Critical Care.* 2013;2):S97.
10. Ashurst JV, Nappe T, Digiambattista S, et al. Effect of triage-based use of the Ottawa foot and ankle rules on the number of orders for radiographic imaging. *The Journal of the American Osteopathic Association.* 2014;114(12):890-897.
11. Badgett JT. Can Medicaid format alter emergency department utilization patterns? *Pediatric Emergency Care.* 1986;2(2):67-70.
12. Barr M, Johnston D, McConnell D. Patient satisfaction with a new nurse practitioner service. *Accident and emergency nursing.* 2000;8(3):144-147.
13. Baughman AW, Li Z, Friedberg MW, et al. Impact of the patient-centered medical home on patients with depression. *Journal of General Internal Medicine.* 2016;1):S265-S266.
14. Beales J. Innovation in accident and emergency management: establishing a nurse practitioner-run minor injuries/primary care unit. *Accident and emergency nursing.* 1997;5(2):71-75.
15. Beales J, Baker B. Minor Injuries Unit: expanding the scope of accident and emergency provision. *Accident and emergency nursing.* 1995;3(2):65-67.
16. Begaz T, Salem R, Agarwal N, Duan L, Taira B. Initiating workups in the waiting room decreases emergency department bed time and left before completion of service rate. *Annals of Emergency Medicine.* 2015;1):S74.
17. Begum F, Khan H, Moss P. Solving the A & E crisis using GP lead triage and redirection Evaluation of GP-lead service to identify and re-direct patients from A & E to primary care services. https://wwwhealthylondonorg/wp-content/uploads/2017/10/30-Solving-the-AE-crisis-using-GP-lead-triage-and-redirectionpdf. 2016.

18. Bitton A. Medical home pilot lowers costs, improves quality and patient/staff experience. *Journal of Clinical Outcomes Management.* 2009;16(12):541-544.
19. Bleijenberg N, Drubbel I, Schuurmans MJ, et al. Effectiveness of a Proactive Primary Care Program on Preserving Daily Functioning of Older People: A Cluster Randomized Controlled Trial. *Journal of the American Geriatrics Society.* 2016;64(9):1779-1788.
20. Bodenmann P, Velonaki VS, Baggio S, et al. Case management for emergency department frequent users: a randomized controlled trial. *Praxis.* 2015;104:17-18.
21. Boeke AJP, van Randwijck-Jacobze ME, de Lange-Klerk EM, Grol SM, Kramer MH, van der Horst HE. Effectiveness of GPs in accident and emergency departments. *The British journal of general practice : the journal of the Royal College of General Practitioners.* 2010;60(579):e378-384.
22. Bonham GS, Barber GM. Use of health care before and during Citicare. *Medical Care.* 1987;25(2):111-119.
23. Bottle A, Honeyford K, Chowdhury F, Bell D, Aylin P. 2018.
24. Breslau J, Leckman-Westin E, Han B, et al. Impact of a mental health based primary care program on emergency department visits and inpatient stays. *General Hospital Psychiatry.* 2018;52:8-13.
25. Buerhaus P, Perloff J, Clarke S, O'Reilly-Jacob M, Zolotusky G, DesRoches CM. Quality of Primary Care Provided to Medicare Beneficiaries by Nurse Practitioners and Physicians. *Medical Care.* 2018;56(6):484-490.
26. Bynum JPW, Andrews A, Sharp S, McCullough D, Wennberg JE. Fewer hospitalizations result when primary care is highly integrated into a continuing care retirement community. *Health Affairs.* 2011;30(5):975-984.
27. Byrne G, Richardson M, Brunsdon J, Patel A. An evaluation of the care of patients with minor injuries in emergency settings. *Accident and emergency nursing.* 2000;8(2):101-109.
28. Campbell S, Atkinson P, Fraser J, Stewart C, Middleton J, McCloskey R. Nurse practitioners in the emergency department: A solution to long wait times and high walkout rates? *Canadian Journal of Emergency Medicine.* 2012;1):S11.
29. Carter A. The Ambulatory Care Unit at Derriford Hospital. *Clinical Medicine.* 2014;14(3):250-254.
30. Carter R, Quesnel-Vallee A, Plante C, Gamache P, Levesque JF. Effect of family medicine groups on visits to the emergency department among diabetic patients in Quebec between 2000 and 2011: a population-based segmented regression analysis. *BMC family practice.* 2016;17:23.
31. Casalino E, Choquet C, Curac S, et al. An evaluation of hospital attractiveness and primary care availability leading to increasing emergency department visits. *Public Health.* 2017;151:27-30.
32. Cecil E, Bottle A, Cowling TE, Majeed A, Wolfe I, Saxena S. Primary care access, emergency department visits, and unplanned short hospitalizations in the UK. *Pediatrics.* 2016;137 (2) (no pagination)(e20151492).
33. Celona CA, Amaranto A, Ferrer R, et al. Interdisciplinary Design to Improve Fast Track in the Emergency Department. *Advanced Emergency Nursing Journal.* 2018;40(3):198-203.
34. Chakravorty S, Knapp CA. The Impact of the Patient-Centered Medical Home on Asthma-Related Visits to the Emergency Room: A Fixed Effects Regression Approach. *Maternal and child health journal.* 2019;23(3):369-376.
35. Chang T, Kuo R. The Association of Quality of Primary Care and the Potentially Avoidable Emergency Department (Ed) Visit. *Value in Health.* 2018;21(Supplement 2):S49.
36. Cheung WWH, Heeney L, Pound JL. An advance triage system. *Accident & Emergency Nursing.* 2002;10(1):10-16.

For peer review only

37. Chmiel C, Wang M, Sidler P, Eichler K, Rosemann T, Senn O. Implementation of a hospital-integrated general practice--a successful way to reduce the burden of inappropriate emergency-department use. *Swiss Medical Weekly.* 2016;146:w14284.
38. Chu L, Sood N, Tu M, Miller K, Ray L, Sayles JN. Reduction of Emergency Department Use in People With Disabilities. *American Journal of Managed Care.* 2017;23(12):e409-e415.
39. Chu LH, Tu M, Lee YC, Sood N. The impact of patient-centered medical homes on safety net clinics. *American Journal of Managed Care.* 2016;22(87):532-538.
40. Clancy E, Mayo A. Launching a social enterprise see-and-treat service. *Emergency nurse : the journal of the RCN Accident and Emergency Nursing Association.* 2009;17(3):22-24.
41. Cloutier MM, Hall CB, Wakefield DB, Bailit H. Use of asthma guidelines by primary care providers to reduce hospitalizations and emergency department visits in poor, minority, urban children. *Journal of Pediatrics.* 2005;146(5):591-597.
42. Colligan M, Collins C, Foley B, Jones P, Miles J, Zeng I. Emergency nurse practitioners: do they provide an effective service in managing minor injuries, compared to emergency medicine registrars? *New Zealand Medical Journal.* 2011;124(1344):74-80.
43. Considine J, Kropman M, Kelly E, Winter C. Effect of emergency department fast track on emergency department length of stay: a case-control study. *Emergency Medicine Journal.* 2008;25(12):815-819.
44. Considine J, Martin R, Smit D, Winter C, Jenkins J. Emergency nurse practitioner care and emergency department patient flow: case-control study. *Emergency Medicine Australasia.* 2006;18(4):385-390.
45. Conway A, Richardson A. The respiratory nurse specialist role at a medical assessment unit. *Nursing times.* 2004;100(24):53-54.
46. Cooper MA, Lindsay GM, Kinn S, Swann IJ. Evaluating Emergency Nurse Practitioner services: a randomized controlled trial. *Journal of advanced nursing.* 2002;40(6):721-730.
47. Copeland J, Gray A. A Daytime Fast Track Improves Throughput in a Single Physician Coverage Emergency Department. *CJEM.* 2015;17(6):648-655.
48. Covington C, Erwin T, Sellers F. Implementation of a nurse practitioner-staffed fast track. *Journal of emergency nursing: JEN : official publication of the Emergency Department Nurses Association.* 1992;18(2):124-131.
49. Crits-Christoph P, Gallop R, Noll E, et al. Impact of a medical home model on costs and utilization among comorbid HIV-positive medicaid patients. *American Journal of Managed Care.* 2018;24(8):368-375.
50. Cuellar A, Helmchen LA, Gimm G, et al. The CareFirst Patient-Centered Medical Home Program: Cost and Utilization Effects in Its First Three Years. *Journal of General Internal Medicine.* 2016;31(11):1382-1388.
51. Dahrouge S, Hogg W, Younger J, Muggah E, Russell G, Glazier RH. Primary Care Physician Panel Size and Quality of Care: A Population-Based Study in Ontario, Canada. *Annals of Family Medicine.* 2016;14(1):26-33.
52. Dale J, Lang H, Roberts JA, Green J, Glucksman E. Cost effectiveness of treating primary care patients in accident and emergency: a comparison between general practitioners, senior house officers, and registrars. *BMJ.* 1996;312(7042):1340-1344.
53. Day TE, Al-Roubaie AR, Goldlust EJ. Decreased length of stay after addition of healthcare provider in emergency department triage: a comparison between computer-simulated and real-world interventions. *Emerg Med J.* 2013;30(2):134-138.
54. Demarco F, Gerardo CJ, Boardwine A, et al. Effect of a nurse rapid intake initiative on patient length of stay and satisfaction: A project IMPACT initiative. *Academic Emergency Medicine.* 2010;17(SUPPL. 1):S93-S94.

For peer review only

55. Derlet RW, Nishio D, Cole LM, Silva J, Jr. Triage of patients out of the emergency department: three-year experience. *The American journal of emergency medicine.* 1992;10(3):195-199.
56. Dewhirst S, Vaillancourt C, Mackenzie T, Zhao Y. Evaluating a nurse initiated analgesia protocol in the emergency department. *Canadian Journal of Emergency Medicine.* 2015;17(Supplement 2):S60-S61.
57. Dias RD, Rios IC, Canhada CL, et al. Using the Manchester triage system for refusing nonurgent patients in the emergency department: A 30-day outcome study. *Journal of Emergency Management.* 2016;14(5):349-364.
58. Dinh M, Walker A, Parameswaran A, Enright N. Evaluating the quality of care delivered by an emergency department fast track unit with both nurse practitioners and doctors. *Australasian Emergency Nursing Journal.* 2012;15(4):188-194.
59. Dixon A, Clarkin C, Barrowman N, Correll R, Osmond MH, Plint AC. Reduction of radial-head subluxation in children by triage nurses in the emergency department: A cluster-randomized controlled trial. *CMAJ.* 2014;186(9):E317-E323.
60. Dolton P, Pathania V. Can increased primary care access reduce demand for emergency care? Evidence from England's 7-day GP opening. *Journal of Health Economics.* 2016;49:193-208.
61. Dorado S, Pascual S, Urrutia I, et al. Prevalence of asthma and management of exacerbations in general practice (GP) after computerised Spanish guideline on the management of asthma (GEMA) and collaborative care environment. *European Respiratory Journal Conference: European Respiratory Society Annual Congress.* 2015;46(SUPPL. 59).
62. Douma MJ, Drake CA, O'Dochartaigh D, Smith KE, O'Dochartaigh D. A Pragmatic Randomized Evaluation of a Nurse-Initiated Protocol to Improve Timeliness of Care in an Urban Emergency Department. *Annals of Emergency Medicine.* 2016;68(5):546-552.
63. Driscoll DL, Hiratsuka V, Johnston JM, et al. Process and outcomes of patient-centered medical care with Alaska Native people at Southcentral Foundation. *Annals of family medicine.* 2013;11(Supplement 1):S41-S49.
64. Ducharme J, Alder RJ, Pelletier C, Murray D, Tepper J. The impact on patient flow after the integration of nurse practitioners and physician assistants in 6 Ontario emergency departments. *CJEM Canadian Journal of Emergency Medical Care.* 2009;11(5):455-461.
65. Edwards T. How rapid assessment at triage can improve care outcomes. *Emergency Nurse.* 2011;19(6):27-30.
66. Eichler K, Hess S, Chmiel C, et al. Sustained health-economic effects after reorganisation of a Swiss hospital emergency centre: a cost comparison study. *Emergency Medicine Journal.* 2014;31(10):818-823.
67. Eichler K, Senn O, Ruthemann I, Bogli K, Sidler P, Brugger U. Reorganisation of hospital emergency services: A business case for quality improvement. *Value in Health.* 2011;14 (7):A344-A345.
68. Fan J, Woolfrey K. The effect of triage-applied Ottawa Ankle Rules on the length of stay in a Canadian urgent care department: a randomized controlled trial. *Academic emergency medicine : official journal of the Society for Academic Emergency Medicine.* 2006;13(2):153-157.
69. Fanta K, Cook B, Falcone RA, et al. Pediatric trauma nurse practitioners provide excellent care with superior patient satisfaction for injured children. *Journal of pediatric surgery.* 2006;41(1):277-281.
70. Farion KJ, Tse S, Patrice J, et al. IMPACT of a tertiary pediatric emergency department ambulatory zone on wait times of both high and low acuity patients. *Canadian Journal of Emergency Medicine.* 2010;12 (3):239.

For peer review only

71. Fleetcroft R, Martin A, Coombes E, Ford J, Steel N, Noble M. Emergency hospital admissions for asthma and access to primary care: Cross-sectional analysis. *British Journal of General Practice.* 2016;66(650):e640-e666.
72. Fontanel A, Besson C, Gallegos C, Vallot C, Droal D. Can X-rays be prescribed by a nurse in an emergency department? *European Journal of Emergency Medicine.* 2011;18(5):310-311.
73. Fry M, Fong J. A 12-month prospective review of the impact of Emergency Transitional Nurse Practitioners in one metropolitan emergency department...2009 CENA International Conference for Emergency Nursing. *Australasian Emergency Nursing Journal.* 2009;12(4):164-165.
74. Fry M, Fong J, Asha S, Arendts G. A 12-month evaluation of the impact of Transitional Emergency Nurse Practitioners in one metropolitan Emergency Department. *Australasian Emergency Nursing Journal.* 2011;14(1):4-8.
75. Gabayan GZ, Asch SM, Starks SL, Sun BC. Does having and using a usual source of care decrease emergency department use? *Annals of Emergency Medicine.* 2009;1):S64.
76. Gardner RM, Friedman N, Bradham T, Barrett TW. Impact of revised triage approach to improving emergency department throughput for treat and release patients. *Academic Emergency Medicine.* 2017;24 (Supplement 1):S157.
77. Gardner RM, Friedman NA, Carlson M, Bradham TS, Barrett TW. Impact of revised triage to improve throughput in an ED with limited traditional fast track population. *American Journal of Emergency Medicine.* 2018;36(1):124-127.
78. Gaucher N, Bailey B, Gravel J. Triage nurses' counselling influences return visits of children leaving the emergency department before being seen by a physician. *Academic Emergency Medicine.* 2010;17(SUPPL. 1):S118-S119.
79. Gedmintas A, Atkinson P, Fraser J, Howlett M. Impact of a new emergency department and process changes on quality indicators. *Canadian Journal of Emergency Medicine.* 2012;1):S11.
80. Grasso MA, Cotter B, Jerrard DA. The impact of a follow-up clinic on unscheduled return visits to an emergency department. *Annals of Emergency Medicine.* 2015;1):S19.
81. Gray C. Advanced practice in emergency care: the paediatric flow nurse. *Nursing Children & Young People.* 2016;28(4):33-38.
82. Green LA, Chang HC, Markovitz AR, Paustian ML. The Reduction in ED and Hospital Admissions in Medical Home Practices Is Specific to Primary Care-Sensitive Chronic Conditions. *Health Services Research.* 2018;53(2):1163-1179.
83. Hackman JL, Roth ED, Gaddis ML, Gratton MC. The effect of a nurse-initiated chest pain protocol on disposition time: A retrospective review. *Annals of Emergency Medicine.* 2015;66(4 SUPPL. 1):S9-S10.
84. Hansagi H. Referral of non-urgent cases from an emergency department: patient compliance, satisfaction and attitudes. *Scandinavian Journal of Social Medicine.* 1990;18(4):249-255.
85. Hansagi H, Allebeck P, Edhag O. Health care utilization after referral from a hospital emergency department. *Scandinavian Journal of Social Medicine.* 1989;17(4):291-299.
86. Harris MD, Kirsh S, Higgins PA. Shared Medical Appointments: Impact on Clinical and Quality Outcomes in Veterans With Diabetes. *Quality management in health care.* 2016;25(3):176-180.
87. Hayden C, Burlingame P, Thompson H, Sabol VK. Improving patient flow in the emergency department by placing a family nurse practitioner in triage: a quality-improvement project. *Journal of Emergency Nursing.* 2014;40(4):346-351.
88. Heaney D, Paxton F. Evaluation of a nurse-led minor injuries unit. *Nursing standard (Royal College of Nursing (Great Britain) : 1987).* 1997;12(4):35-38.

For peer review only

89. Hearld KR, Hearld LR, Landry AY, Budhwani H. Evidence that patient-centered medical homes are effective in reducing emergency department admissions for patients with depression. *Health Services Management Research.* 2018:951484818794340.
90. Hearld LR, Alexander JA. Patient-centered care and emergency department utilization: a path analysis of the mediating effects of care coordination and delays in care. *Medical Care Research & Review.* 2012;69(5):560-580.
91. Hearld LR, Hearld KR, Guerrazzi C. Patient-Centered Medical Home Capacity and Ambulatory Care Utilization. *American Journal of Medical Quality.* 2017;32(5):508-517.
92. Hebert P, Liu CF, Wong E, et al. National evaluation of the effects on healthcare utilization and costs of the va patient centered medical home initiative. *Journal of General Internal Medicine.* 2013;1):S124.
93. Ho JK-M, Chau JP-C, Chan JT-S, Yau CH-Y. Nurse-initiated radiographic-test protocol for ankle injuries: A randomized controlled trial. *International emergency nursing.* 2018;41:1-6.
94. Hunold KM, Richmond NL, Waller AE, Cutchin MP, Voss PR, Platts-Mills TF. Primary care availability and emergency department use by older adults: A population-based analysis. *Journal of the American Geriatrics Society.* 2014;62(9):1699-1706.
95. Hwang W, Liao K, Griffin L, Foley KL. Do free clinics reduce unnecessary emergency department visits? The Virginian experience. *Journal of Health Care for the Poor and Underserved.* 2012;23(3):1189-1204.
96. Ingram SJ, Maloney A, McDonnell N, et al. Impact of a nurse led ambulatory low-intermediate risk chest pain service. *European Heart Journal: Acute Cardiovascular Care.* 2013;1):127.
97. James D, Kithany H, Robbins A, Crouch R. Children and young people seen in a colocated primary care hub; an observational study. *Archives of Disease in Childhood.* 2019;104(Supplement 2):A134.
98. Jamshed N, Cawthon C, Rubin C. Impact analysis of a home based primary care program (HBPC) next generation aco (NGACO) model. *Journal of the American Geriatrics Society.* 2018;66 (Supplement 2):S122.
99. Jeanmonod R, Delcollo J, Jeanmonod D, Dombchewsky O, Reiter M. Comparison of resident and mid-level provider productivity and patient satisfaction in an emergency department fast track. *Emergency Medicine Journal.* 2013;30(1):e12.
100. Jennings N, Gardner G, O'Reilly G, Mitra B. Emergency NP Model of Care in an Australian Emergency Department. *Journal for Nurse Practitioners.* 2015;11(8):774-781.
101. Jennings N, O'Reilly G, Lee G, Cameron P, Free B, Bailey M. Evaluating outcomes of the emergency nurse practitioner role in a major urban emergency department, Melbourne, Australia. *Journal of Clinical Nursing.* 2008;17(8):1044-1050.
102. Jobé J, Vandercleyen C, Ghuysen A, D'Orio V. Prospective study of an advanced nurse triage for a target pathology at the admission in the emergency department. *Acta clinica belgica.* 2013;68(6):482-.
103. Johnson P, Linzer M, Shippee ND, Heegaard W, Webb F, Vickery KD. Development and Implementation of an Interdisciplinary Intensive Primary Care Clinic for High-Need High-Cost Patients in a Safety Net Hospital. *Population health management.* 2019.
104. Jones D. How GPs can help reduce inappropriate attendance. *Emergency Nurse.* 2011;19(4):20-23.
105. Kao YH, Tseng TS, Ng YY, Wu SC. Association between continuity of care and emergency department visits and hospitalization in senior adults with asthma-COPD overlap. *Health Policy.* 2019;123(2):222-228.

For peer review only

106. Keogh C, O'Brien KK, Hoban A, O'Carroll A, Fahey T. Health and use of health services of people who are homeless and at risk of homelessness who receive free primary health care in Dublin. *BMC health services research.* 2015;15:58.
107. Kern LM, Seirup JK, Rajan M, Jawahar R, Stuard SS. Fragmented ambulatory care and subsequent healthcare utilization among medicare beneficiaries. *American Journal of Managed Care.* 2018;24(9):e278-e284.
108. Kern LM, Seirup JK, Rajan M, Jawahar R, Stuard SS. Fragmented ambulatory care and subsequent emergency department visits and hospital admissions among Medicaid beneficiaries. *The American journal of managed care.* 2019;25(3):107-112.
109. Kim KM, Jeon H, Lee JH. Having a Physician Rather than a Place as a Usual Source of Care Would Be Better - from 2012 Korea Health Panel Data. *Journal of Korean medical science.* 2017;32(1):4-12.
110. Kim M, Asche CV, Tillis W, Ren J. Association between availability of care providers and healthcare utilizations among adults with asthma. *Current Medical Research & Opinion.* 2017;33(3):479-487.
111. Klassen TP, Ropp LJ, Sutcliffe T, et al. A randomized, controlled trial of radiograph ordering for extremity trauma in a pediatric emergency department. *Annals of emergency medicine.* 1993;22(10):1524-1529.
112. Klemmer DK, Ziebel C, Sharif N, Grubb S, Sookram S. Clinical lead nurse practitioner Strathcona Community Hospital. *Canadian Journal of Emergency Medicine.* 2018;20 (Supplement 1):S85.
113. Kobewka DM, Kunkel E, Hsu A, Talarico R, Tanuseputro P. Physician Availability in Long-term Care and Resident Hospital Transfer: A Retrospective Cohort Study. *Journal of the American Medical Directors Association.* 2019.
114. Kool RB, Homberg DJ, Kamphuis HC. Towards integration of general practitioner posts and accident and emergency departments: a case study of two integrated emergency posts in the Netherlands. *BMC Health Services Research.* 2008;8:225.
115. Krakau I. Trends in use of health care services in Swedish primary care district. A ten year perspective. *Scandinavian Journal of Primary Health Care.* 1992;10(1):66-71.
116. Kravet SJ, Shore AD, Miller R, Green GB, Kolodner K, Wright SM. Health Care Utilization and the Proportion of Primary Care Physicians. *American Journal of Medicine.* 2008;121(2):142-148.
117. Krook CE, Parks P. Emergency department follow-up for unattached patients. *Canadian Journal of Emergency Medicine.* 2013;15 (Suppl 1):S5.
118. Krupski A, West II, Scharf DM, et al. Integrating primary care into community mental health centers: Impact on utilization and costs of health care. *Psychiatric Services.* 2016;67(11):1233-1239.
119. Kuo Y-F, Adhikari D, Eke CG, Goodwin JS, Raji MA. Processes and Outcomes of Congestive Heart Failure Care by Different Types of Primary Care Models. *Journal of cardiac failure.* 2018;24(1):9-18.
120. Lagisetty PA, Hu HM, Goesling J, et al. Care coordination and adverse opioid related outcomes: The role of the usual provider. *Journal of General Internal Medicine.* 2018;33(2 Supplement 1):134.
121. Laine C, Lin YT, Hauck WW, Turner BJ. Availability of medical care services in drug treatment clinics associated with lower repeated emergency department use. *Medical Care.* 2005;43(10):985-995.

For peer review only

122. Lee C, Sung NJ, Lim HS, Lee JH. Emergency Department Visits Can Be Reduced by Having a Regular Doctor for Adults with Diabetes Mellitus: Secondary Analysis of 2013 Korea Health Panel Data. *Journal of Korean Medical Science.* 2017;32(12):1921-1930.
123. Lee GA, Jennings N. A comparative study of patients who did not wait for treatment and those treated by emergency nurse practitioners. *Australasian Emergency Nursing Journal.* 2006;9(4):179-185.
124. Lee KM, Wong TW, Chan R, Lau CC, Fu YK, Fung KH. Accuracy and efficiency of X-ray requests initiated by triage nurses in an accident and emergency department. *Accident and emergency nursing.* 1996;4(4):179-181.
125. Lee MH, Effenberger A. Effect of formation of an accountable care organization on characteristics of emergency department visits. *Academic Emergency Medicine.* 2014;1):S219.
126. Lee WW, Filiatrault L, Abu-Laban RB, Rashidi A, Yau L, Liu N. Effect of triage nurse initiated radiography using the Ottawa Ankle Rules on emergency department length of stay at a tertiary care center. *Canadian Journal of Emergency Medicine.* 2014;16(SUPPL. 1):S38.
127. Lee WW, Filiatrault L, Abu-Laban RB, Rashidi A, Yau L, Liu N. Effect of Triage Nurse Initiated Radiography Using the Ottawa Ankle Rules on Emergency Department Length of Stay at a Tertiary Centre. *CJEM.* 2016;18(2):90-97.
128. Li Y, Lu Q, Du H, Zhang J, Zhang L. The Impact of Triage Nurse-ordered Diagnostic Studies on Pediatric Emergency Department Length of Stay. *Indian journal of pediatrics.* 2018;85(10):849-854.
129. Liferidge A, McCarthy M, Blanchard J, Ding R, Li S. Triage of low acuity emergency department patients to a primary care clinic and medical home: A utilization and cost effectiveness analysis. *Annals of Emergency Medicine.* 2015;1):S87-S88.
130. Lijuan Z. Advanced Triage Protocols in the Emergency Department. *Advanced Triage Protocols in the Emergency Department.* 2017:1-1.
131. Lindley-Jones M, Finlayson BJ. Triage nurse requested x rays--are they worthwhile? *J Accid Emerg Med.* 2000;17(2):103-107.
132. Lindly OJ, Zuckerman KE, Kuhlthau KA. Healthcare access and services use among US children with autism spectrum disorder. *Autism : the international journal of research and practice.* 2018:1362361318815237.
133. Lippi Bruni M, Mammi I, Ugolini C. Does the extension of primary care practice opening hours reduce the use of emergency services? *Journal of Health Economics.* 2016;50:144-155.
134. Long SK, Zuckerman S. Urban health care in transition: Challenges facing Los Angeles County. *Health Care Financing Review.* 1998;20(1):45-58.
135. Love RA, Murphy JA, Lietz TE, Jordan KS. The effectiveness of a provider in triage in the emergency department: a quality improvement initiative to improve patient flow. *Advanced Emergency Nursing Journal.* 2012;34(1):65-74.
136. Lowe RA. Better access to primary care may reduce ED use in Medicaid managed care. *Capitation rates & data.* 2006;11(5):57-60.
137. Lowe RA, Fu R, Ong ET, et al. Community characteristics affecting emergency department use by medicaid enrollees. *Medical Care.* 2009;47(1):15-22.
138. Lukes T, Schjodt K, Struwe L. Implementation of a Nursing Based Order Set: Improved Antibiotic Administration Times for Pediatric ED Patients with Therapy-Induced Neutropenia and Fever. *Journal of Pediatric Nursing.* 2019;46:78-82.
139. Lutze M, Ratchford A, Fry M. A review of the Transitional Emergency Nurse Practitioner. *Australasian Emergency Nursing Journal.* 2011;14(4):226-231.
140. Lynch C, Wajnberg A, Jacob M, et al. Intensive primary care for high readmission risk adults. *Journal of General Internal Medicine.* 2014;1):S488-S489.

For peer review only

141. MacKenzie RS, Burmeister DB, Brown JA, et al. Implementation of a rapid assessment unit (intake team): impact on ED length of stay. *American Journal of Emergency Medicine.* 2015;33(2):291-293.
142. MacKenzie RS, Burmeister DB, Brown JA, et al. Implementation of a rapid assessment unit (intake team): Impact on emergency department length of stay. *Annals of Emergency Medicine.* 2013;1):S12-S13.
143. Mackinney T, Visotcky AM, Tarima S, Whittle J. Does providing care for uninsured patients decrease emergency room visits and hospitalizations? *Journal of Primary Care & Community Health.* 2013;4(2):135-142.
144. Manns BJ, Tonelli M, Zhang J, et al. Enrolment in primary care networks: Impact on outcomes and processes of care for patients with diabetes. *Cmaj.* 2012;184(2):E144-E152.
145. Martin G, McGowan M, Postic M, Gaunt K. Level of patient satisfaction with the NP role in fast track: preliminary findings. *Canadian Journal of Emergency Medicine.* 2013;15 (Suppl 1):S1.
146. Martin M, Girma F, Rushing J. Care plus: Five years of providing team based care to high need high cost patients. *Journal of General Internal Medicine.* 2019;34(2 Supplement):S711.
147. McAlister FA, Bakal JA, Green L, Bahler B, Lewanczuk R. The effect of provider affiliation with a primary care network on emergency department visits and hospital admissions. *CMAJ.* 2018;190(10):E276-E284.
148. McCarron M, Burgess K, Gibbs S. An evaluation of primary care streaming in a tertiary paediatric emergency department. *Archives of Disease in Childhood.* 2019;104(Supplement 2):A138-A139.
149. McClellan C, Cramp F, Powell J, Benger JR. Clinical and cost effectiveness of different emergency department healthcare professionals in the management of musculoskeletal soft tissue injuries. *Physiotherapy (United Kingdom).* 2011;1):eS776-eS777.
150. McDonnell WM, Carpenter P, Jacobsen K, Kadish HA. Relative productivity of nurse practitioner and resident physician care models in the pediatric emergency department. *Pediatric Emergency Care.* 2015;31(2):101-106.
151. Megahy A, Lloyd M. Managing minor injuries. *Emergency nurse : the journal of the RCN Accident and Emergency Nursing Association.* 2004;12(5):14-16.
152. Michael L, Brady AK, Russell G, et al. Connecting Refugees to Medical Homes Through Multi-Sector Collaboration. *Journal of immigrant and minority health.* 2019;21(1):198-203.
153. Miller D, Loftus AM, O'Boyle PJ, et al. Impact of a telephone-first consultation system in general practice. *Postgraduate medical journal.* 2019;95(1129):590-595.
154. Mitchell EA, Didsbury PB, Kruithof N, et al. A randomized controlled trial of an asthma clinical pathway for children in general practice. *Acta Paediatrica, International Journal of Paediatrics.* 2005;94(2):226-233.
155. Mittmann N, Beglaryan H, Liu N, et al. Examination of health system resources and costs associated with transitioning cancer survivors to primary care: A propensity-score-matched cohort study. *Journal of Oncology Practice.* 2018;14(11).
156. Moe J, Oland R, Moe G. Impact of a Primary Care After-Hours Clinic on Avoidable Emergency Department Visits and Costs. *Healthcare quarterly (Toronto, Ont).* 2019;22(1):42-47.
157. Morin C, Choukroun J, Callahan JC. Safety and efficiency of a redirection procedure toward an out of hours general practice before admission to an emergency department, an observational study. *BMC Emergency Medicine.* 2018;18(1):26.
158. Muller K, Chee Z, Doan Q. Using Nurse Practitioners to Optimize Patient Flow in a Pediatric Emergency Department. *Pediatric Emergency Care.* 2018;34(6):396-399.

For peer review only

159. Munday T, Forbes M, Little J, Warren G, Bowe P, Lind J. The patient flow effects of a new medical assessment unit within a large urban health service. *Internal Medicine Journal.* 2012;42 (SUPPL.2):9-10.

160. Murphy AW, Bury G, Plunkett PK, et al. Randomised controlled trial of general practitioner versus usual medical care in an urban accident and emergency department: process, outcome, and comparative cost. *BMJ (clinical research ed).* 1996;312(7039):1135-1142.

161. Musich S, Wang S, Hawkins K, Klemes A. The impact of personalized preventive care on health care quality, utilization, and expenditures. *Population Health Management.* 2016;19(6):389-397.

162. Nash K, Zachariah B, Nitschmann J, Psencik B. Evaluation of the fast track unit of a university emergency department. *JEN: Journal of Emergency Nursing.* 2007;33(1):14-94.

163. Nelson KM, Sylling PW, Taylor L, et al. Improved team function associated with lower health care use. *Journal of General Internal Medicine.* 2016;1):S269.

164. Newman RJ, Bikowski R, Nakayama K, Cunningham T, Acker P, Bradshaw D. Outcomes of Embedded Care Management in a Family Medicine Residency Patient-Centered Medical Home. *Family medicine.* 2017;49(1):46-51.

165. Nguyen ND, Moore JB, McIntosh NP, Jones ML, Zimmerman J, Summers RL. Emergency department triage of low acuity patients to a Federally Qualified Health Center. *Journal of the Mississippi State Medical Association.* 2013;54(10):280-283.

166. Nichols DE, Haber SG, Romaire MA, Wensky SG, Multi-Payer Advanced Primary Care Practice Evaluation T. Changes in Utilization and Expenditures for Medicare Beneficiaries in Patient-centered Medical Homes: Findings From the Multi-Payer Advanced Primary Care Practice Demonstration. *Medical care.* 2018;56(9):775-783.

167. Nyweide DJ, Bynum JPW. Relationship Between Continuity of Ambulatory Care and Risk of Emergency Department Episodes Among Older Adults. *Annals of Emergency Medicine.* 2017;69(4):407-415.e403.

168. O'Keeffe N. The effect of a new general practice out-of-hours co-operative on a county hospital accident and emergency department. *Irish journal of medical science.* 2008;177(4):367-370.

169. O'Kelly FD, Teljeur C, Carter I, Plunkett PK. Impact of a GP cooperative on lower acuity emergency department attendances. *Emergency Medicine Journal.* 2010;27(10):770-773.

170. Olsen JC, Ogarek J, Goldenberg E, Sulo S. Effect of a chronic pain protocol on emergency department utilization. *Academic Emergency Medicine.* 2015;1):S151.

171. Organ K, Chinnick P, Higgison I, Stanhope B, Hoskins R, Benger J. Evaluating the introduction of a paediatric emergency nurse practitioner service. *Emergency nurse : the journal of the RCN Accident and Emergency Nursing Association.* 2005;13(7):8-11.

172. O'Toole TP, Buckel L, Bourgault C, et al. Applying the chronic care model to homeless veterans: effect of a population approach to primary care on utilization and clinical outcomes. *American journal of public health.* 2010;100(12):2493-2499.

173. Owens D, Eby K, Burson S, Green M, McGoodwin W, Isaac M. Primary Palliative Care Clinic Pilot Project demonstrates benefits of a nurse practitioner-directed clinic providing primary and palliative care. *Journal of the American Academy of Nurse Practitioners.* 2012;24(1):52-58.

174. Parris W, McCarthy S, Kelly AM, Richardson S. Do triage nurse-initiated X-rays for limb injuries reduce patient transit time? *Accid Emerg Nurs.* 1997;5(1):14-15.

175. Parsons PL, McMurtry CT. NP care/discharge planning saves money. *The Nurse practitioner.* 1997;22(3):238-240.

For peer review only

176. Patel H, Celenza A, Watters T. Effect of nurse initiated X-rays of the lower limb on patient transit time through the emergency department. *Australasian Emergency Nursing Journal.* 2012;15(4):229-234.
177. Peterson GG, Geonnotti KL, Hula L, et al. Association between extending carefirst's medical home program to medicare patients and quality of care, utilization, and spending. *JAMA Internal Medicine.* 2017;177(9):1334-1342.
178. Philips H, Remmen R, Van Royen P, et al. What's the effect of the implementation of general practitioner cooperatives on caseload? Prospective intervention study on primary and secondary care. *BMC Health Services Research.* 2010;10:222.
179. Phillips RL, Bronnikov S, Petterson S, et al. Case study of a primary care-based accountable care system approach to medical home transformation. *Journal of Ambulatory Care Management.* 2011;34(1):67-77.
180. Pickin DM, O'Cathain A, Fall M, Morgan AB, Howe A, Nicholl JP. The impact of a general practice co-operative on accident and emergency services, patient satisfaction and GP satisfaction. *Family Practice.* 2004;21(2):180-182.
181. Pierce BA, Gormley D. Are Split Flow and Provider in Triage Models in the Emergency Department Effective in Reducing Discharge Length of Stay? *Journal of Emergency Nursing.* 2016;42(6):487-491.
182. Pirraglia PA, Rowland E, Wu WC, et al. Benefits of a primary care clinic co-located and integrated in a mental health setting for veterans with serious mental illness. *Preventing chronic disease.* 2012;9:E51.
183. Platter MEM, Kurvers RAJ, Janssen L, Verweij MMJ, Barten DG. The impact of an emergency care access point on pediatric attendances at the emergency department: An observational study. *The American journal of emergency medicine.* 2019.
184. Prescott C, Stackhouse N. Developing an advanced nurse practitioner approach to clinical assessments. *Emergency Nurse.* 2017;24(9):33-37.
185. Preston-Suni K, Fleischman R, Ramon J, et al. Triage improvements reduce wait times and eliminate disparities for patients with limited english proficiency. *Academic Emergency Medicine.* 2019;26(Supplement 1):S84.
186. Raffetto B, Balingit P. Safety net hospitals: Are medical homes the answer to overcrowding emergency departments? *Academic Emergency Medicine.* 2014;1):S221.
187. Raphael JL, Cooley WC, Vega A, et al. Outcomes for Children with Chronic Conditions Associated with Parent- and Provider-reported Measures of the Medical Home. *Journal of health care for the poor and underserved.* 2015;26(2):358-376.
188. Rautava V, Valpas T, Nurmikari M, Palomaki A. Establishing a new emergency department: Effects on patient flow. *Critical Care.* 2013;2):S97.
189. Ravichandran Y, Parker SJ, Mowbray FI, et al. Examining utility of advanced practice providers for asthma management in a pediatric emergency department. *Academic Emergency Medicine.* 2019;26(Supplement 1):S298.
190. Ray M, Reinoso H. A New Process to Improve Throughput in the Emergency Department. *Journal for Nurse Practitioners.* 2019;15(10):e193-e196.
191. Reich H, Nwogu N, Garcia-Jimenez MD, et al. Ambulatory care visits increase emergency room visits and admissions in an urban, resident-run clinic. *Journal of General Internal Medicine.* 2018;33 (2 Supplement 1):109.
192. Retezar R, Bessman E, Ding R, Zeger SL, McCarthy ML. The effect of triage diagnostic standing orders on emergency department treatment time. *Annals of emergency medicine.* 2011;57(2):89-99.e82.

For peer review only

193. Rhee KJ, Dermyer AL. Patient satisfaction with a nurse practitioner in a university emergency service. *Annals of Emergency Medicine.* 1995;26(2):130-132.
194. Rivara RP, Wall HP, Worley P, James KD. Pediatric nurse triage: Its efficacy, safety, and implications for care. *American Journal of Diseases of Children.* 1986;140(3):205-210.
195. Riverin BD, Li P, Naimi AI, Strumpf E. Team-based versus traditional primary care models and short-term outcomes after hospital discharge. *Cmaj.* 2017;189(16):E585-E593.
196. Rogers T, Ross N, Spooner D. Evaluation of a 'see and treat' pilot study introduced to an emergency department. *Accident and Emergency Nursing.* 2004;12(1):24-27.
197. Rosenthal MB, Alidina S, Friedberg MW, et al. A Difference-in-Difference Analysis of Changes in Quality, Utilization and Cost Following the Colorado Multi-Payer Patient-Centered Medical Home Pilot. *Journal of General Internal Medicine.* 2016;31(3):289-296.
198. Rosenthal MB, Friedberg MW, Singer SJ, Eastman D, Li Z, Schneider EC. Effect of a multipayer patient-centered medical home on health care utilization and quality: The rhode island chronic care sustainability initiative pilot program. *JAMA Internal Medicine.* 2013;173(20):1907-1913.
199. Rothkopf J, Brookler K, Wadhwa S, Sajovetz M. Medicaid patients seen at federally qualified health centers use hospital services less than those seen by private providers. *Health Affairs.* 2011;30(7):1335-1342.
200. Rothwell S, McIltrot K, Khouri-Stevens Z. Addressing Emergency Department Issues Using Advanced Practice in Saudi Arabia. *Journal for Nurse Practitioners.* 2018;14(2):e41-e44.
201. Rust G, Baltrus P, Ye J, et al. Presence of a community health center and uninsured emergency department visit rates in rural counties. *Journal of Rural Health.* 2009;25(1):8-16.
202. Rutten MH, Smits M, Peters YAS, Assendelft WJJ, Westert GP, Giesen PHJ. Effects of access to radiology in out-of-hours primary care in the Netherlands: a prospective observational study. *Family Practice.* 2018;35(3):253-258.
203. Salisbury C, Hollinghurst S, Montgomery A, et al. The impact of co-located NHS walk-in centres on emergency departments. *Emergency Medicine Journal.* 2007;24(4):265-269.
204. Sanchez M, Smally AJ, Grant RJ, Jacobs LM. Effects of a fast-track area on emergency department performance. *J Emerg Med.* 2006;31(1):117-120.
205. Savage AI, Lauby T, Burkard JF. Examining selected patient outcomes and staff satisfaction in a primary care clinic at a military treatment facility after implementation of the patient-centered medical home. *Military medicine.* 2013;178(2):128-134.
206. Schmidt EK, Hess A, Hanks C, Hand B. A Patient-Centered Medical Home for Young Adults with Autism and Subsequent Emergency Department Utilization. *Archives of Physical Medicine and Rehabilitation.* 2019;100(10):e35-e36.
207. Schwartzman K, Duquette G, Zaoude M, et al. Respiratory day hospital: A novel approach to acute respiratory care. *Cmaj.* 2001;165(8):1067-1071.
208. Seaberg DC, Hennings J, Good M, et al. Redirecting low-acuity pediatric emergency department patients to a hospital-based federally qualified health center. *Academic Emergency Medicine.* 2010;1):S96.
209. Sharma A, Inder B. Impact of co-located general practitioner (GP) clinics and patient choice on duration of wait in the emergency department. *Emergency Medicine Journal.* 2011;28(8):658-661.
210. Shea SS, Hoyt KS. "RAPID" Team triage: One hospitals approach to patient-centered team triage. *Advanced Emergency Nursing Journal.* 2012;34(2):177-189.
211. Sheikh S, Pitts J, Ryan-Wenger N, Hayes D, McCoy K. Impact of type of health-care provider on long-term asthma care in children: A model for primary care. *Chest.* 2016;150 (4 Supplement 1):886A.

For peer review only

212. Shrimpling M. Redesigning triage to reduce waiting times. *Emerg Nurse.* 2002;10(2):34-37.
213. Shumacher RJ, Meyer J, Johnson B, Grisham T, Chamberlain L, Jennings J. Clinical impact of optumTM careplus on nursing homes and residents. *Journal of the American Geriatrics Society.* 2014;1):S15.
214. Sikkenga T, Dumkow L, Draper H, et al. Implementation of a nursing triage order to improve utilization of rapid diagnostic testing for chlamydia and gonorrhea in the emergency department. *Open Forum Infectious Diseases.* 2016;3(Supplement 1).
215. Sinclair L, Hunter R, Hagen S, Nelson D, Hunt J. How effective are mental health nurses in A&E departments? *Emergency medicine journal.* 2006;23(9):687-692.
216. Sjonell G. Effect of establishing a primary health care center on the utilization of primary health care and other out-patient care in a Swedish urban area. *Family Practice.* 1986;3(3):148-154.
217. Sledge WH, Brown KE, Levine JM, et al. A randomized trial of primary intensive care to reduce hospital admissions in patients with high utilization of inpatient services. *Disease Management.* 2006;9(6):328-338.
218. Smith JJ, Johnston JM, Hiratsuka VY, Dillard DA, Tierney S, Driscoll DL. Medical home implementation and trends in diabetes quality measures for AN/AI primary care patients. *Primary Care Diabetes.* 2015;9(2):120-126.
219. Smith L, Narang Y, Ibarz Pavon AB, et al. To GP or not to GP: a natural experiment in children triaged to see a GP in a tertiary paediatric emergency department (ED). *BMJ Quality & Safety.* 2018;27(7):521-528.
220. Smith-Campbell B. Emergency department and community health center visits and costs in an uninsured population. *Journal of nursing scholarship : an official publication of Sigma Theta Tau International Honor Society of Nursing / Sigma Theta Tau.* 2005;37(1):80-86.
221. Sprandio JD. Oncology patient-centered medical home and accountable cancer care. *Community Oncology.* 2010;7(12):565-572.
222. Steiner IP, Nichols DN, Blitz S, et al. Impact of a nurse practitioner on patient care in a Canadian emergency department. *CJEM Canadian Journal of Emergency Medical Care.* 2009;11(3):207-214.
223. Sterner SE, Coco T, Monroe KW, King WD, Losek JD. A new after-hours clinic model provides cost-saving, faster care compared with a pediatric emergency department. *Pediatric Emergency Care.* 2012;28(11):1162-1165.
224. Stoddart D, Ireland AJ, Crawford R, Kelly B. Impact on an accident and emergency department of Glasgow's new primary care emergency service. *Health bulletin.* 1999;57(3):186-191.
225. Stone M, Nguygen ND, Moore JB, et al. Emergency department triage of low acuity patients to a federally qualified health center. *Annals of Emergency Medicine.* 2013;1):S46-S47.
226. Strange P. The nurse practitioner in A&E. *Australian nursing journal (July 1993).* 1994;1(9):20-23.
227. Subash F, Dunn F, McNicholl B, Marlow J. Team triage improves emergency department efficiency. *Emergency medicine journal.* 2004;21(5):542-544.
228. Sukpraprut-Braaten S, Henderson JC, Kinchen DL, et al. Medical screening exam utilized to manage emergent and non-emergent patients presenting to the emergency department. *Annals of Emergency Medicine.* 2016;68 (4 Supplement 1):S71.
229. Sung NJ, Choi YJ, Lee JH. Primary Care Comprehensiveness Can Reduce Emergency Department Visits and Hospitalization in People with Hypertension in South Korea. *International Journal of Environmental Research & Public Health [Electronic Resource].* 2018;15(2):05.

For peer review only

230. Terp S, Mehta R, Arora S, Goldman D, Romley J, Menchine M. Evaluating the association between primary care provider availability and ed visits and hospitalizations for ambulatory care sensitive conditions in California. *Academic Emergency Medicine.* 2013;1):S295.

231. Thijssen W, Kraaijvanger N, Barten DG, Boerma MLM, Giesen P, Wensing M. Impact of a well-developed primary care system on the length of stay in emergency departments in the Netherlands: a multicenter study. *BMC Health Services Research.* 2016;16:149.

232. Thijssen WA, Wijnen-van Houts M, Koetsenruijter J, Giesen P, Wensing M. The impact on emergency department utilization and patient flows after integrating with a general practitioner cooperative: an observational study. *Emergency Medicine International Print.* 2013;2013:364659.

233. Thompson K, Etheridge L. The impact of children's ambulatory unit model of care on children's emergency department flow. *Archives of Disease in Childhood.* 2019;104(Supplement 2):A129.

234. Thompson W, Meskell P. Evaluation of an Advanced Nurse Practitioner (Emergency Care)—An Irish Perspective. *Journal for Nurse Practitioners.* 2012;8(3):200-205.

235. Thrasher C, Purc-Stephenson R. Patient satisfaction with nurse practitioner care in emergency departments in Canada. *Journal of the American Academy of Nurse Practitioners.* 2008;20(5):231-237.

236. Thurston J, Field S. Should accident and emergency nurses request radiographs? Results of a multicentre evaluation. *J Accid Emerg Med.* 1996;13(2):86-89.

237. Timbie JW, Setodji CM, Kress A, et al. Implementation of medical homes in Federally Qualified Health Centers. *New England Journal of Medicine.* 2017;377(3):246-256.

238. To T, Guan J, Zhu J, et al. Quality of asthma care under different primary care models in Canada: a population-based study. *BMC family practice.* 2015;16:19.

239. Traub SJ, Wood JP, Kelley J, et al. Emergency department rapid medical assessment: overall effect and mechanistic considerations. *Journal of Emergency Medicine.* 2015;48(5):620-627.

240. Tsai MH, Xirasagar S, Carroll S, et al. Reducing High-Users' Visits to the Emergency Department by a Primary Care Intervention for the Uninsured: A Retrospective Study. *Inquiry : a journal of medical care organization, provision and financing.* 2018;55:46958018763917.

241. Tsai V, Harley J, Sharieff G, Carlson LA, Kanegaye J. Rapid medical assessment: Improved patient flow and left without being seen rates. *Pediatric Emergency Care.* 2009;25 (10):706.

242. Tsai VW, Sharieff GQ, Kanegaye JT, Carlson LA, Harley J. Rapid medical assessment: improving pediatric emergency department time to provider, length of stay, and left without being seen rates. *Pediatric Emergency Care.* 2012;28(4):354-356.

243. Tucker A, Bernard M. Making the Case for Nurse Practitioners in the Emergency Department: A Clinical Case Study. *Advanced Emergency Nursing Journal.* 2015;37(4):308-312.

244. Tucker S, Jiang R, Beharry A, Raman A, Jubelt LE. Turn no patient away: Adopting advanced access scheduling in primary care. *Journal of General Internal Medicine.* 2018;33(2 Supplement 1):839-840.

245. Uthman OA, Walker C, Lahiri S, et al. General practitioners providing non-urgent care in emergency department: a natural experiment. *BMJ Open.* 2018;8(5):e019736.

246. van den Bersselaar DLCM, Maas M, Thijssen WAMH. Does X-ray imaging by GPC at emergency care access points in the Netherlands change patient flow and reduce ED crowding? A cohort study. *Health science reports.* 2018;1(2):e26.

247. van der Linden C, Reijnen R, de Vos R. Diagnostic accuracy of emergency nurse practitioners versus physicians related to minor illnesses and injuries. *Journal of Emergency Nursing.* 2010;36(4):311-316.

For peer review only

248. van Gils-van Rooij ESJ, Meijboom BR, Broekman SM, Yzermans CJ, de Bakker DH. Is patient flow more efficient in Urgent Care Collaborations? *European Journal of Emergency Medicine.* 2018;25(1):58-64.
249. Van Uden CJT, Ament AJHA, Voss GBWE, et al. Out-of-hours primary care. Implications of organisation on costs. *BMC Family Practice.* 2006;7(no pagination).
250. Van Uden CJT, Winkens RAG, Wesseling GJ, Crebolder HFJM, Van Schayck CP. Use of out of hours services: A comparison between two organisations. *Emergency Medicine Journal.* 2003;20(2):184-187.
251. van Veelen MJ, van den Brand CL, Reijnen R, van der Linden MC. Effects of a general practitioner cooperative co-located with an emergency department on patient throughput. *World journal of emergency medicine.* 2016;7(4):270-273.
252. Vinton D, Riordan JP, O'Conner R, Joseph JW. Impact of the management of low-acuity patients at triage on their length of stay. *Academic Emergency Medicine.* 2019;26(Supplement 1):S235-S236.
253. Voelker J, Hegarty SE, Keith SW, Alcusky M, Lombardi M, Maio V. Do medical homes affect healthcare utilization rates? a longitudinal evaluation on medical home implementation in Italy. *Value in Health.* 2018;21(Supplement 1):S133.
254. Wallis M, Marsden E, Taylor A, et al. The Geriatric Emergency Department Intervention model of care: a pragmatic trial. *BMC geriatrics.* 2018;18(1):297.
255. Wand T, D'Abrew N, Acret L, White K. Evaluating a new model of nurse-led emergency department mental health care in Australia; perspectives of key informants. *International emergency nursing.* 2016;24:16-21.
256. Wand T, D'Abrew N, Barnett C, Acret L, White K. Evaluation of a nurse practitioner-led extended hours mental health liaison nurse service based in the emergency department. *Australian Health Review.* 2015;39(1):1-8.
257. Wang M, Wild S, Hilfiker G, et al. Hospital-integrated general practice: a promising way to manage walk-in patients in emergency departments. *Journal of Evaluation in Clinical Practice.* 2014;20(1):20-26.
258. Watts B, Lawrence RH, Carter C, et al. Does intensive ambulatory management of high risk patients reduce acute care utilization? a pilot study. *Journal of General Internal Medicine.* 2016;1):S199.
259. Weinberger M, Oddone EZ, Henderson WG. Does increased access to primary care reduce hospital readmissions? *New England Journal of Medicine.* 1996;334(22):1441-1447.
260. Wilson A, Shifaza F. An evaluation of the effectiveness and acceptability of nurse practitioners in an adult emergency department. *International Journal of Nursing Practice.* 2008;14(2):149-156.
261. Winston S. Nurse practitioners in the emergency department: a case study of the Washington Hospital Center, Washington, D.C. *Hosp Top.* 1981;59(4):10-13.
262. Wright B, Potter AJ, Trivedi AN, Mueller KJ. The Relationship Between Rural Health Clinic Use and Potentially Preventable Hospitalizations and Emergency Department Visits Among Medicare Beneficiaries. *The Journal of rural health : official journal of the American Rural Health Association and the National Rural Health Care Association.* 2018;34(4):423-430.
263. Wright DB, Ricketts TC. The road to efficiency? Re-examining the impact of the primary care physician workforce on health care utilization rates. *Social Science and Medicine.* 2010;70(12):2006-2010.
264. Wright SW, Erwin TL, Blanton DM, Covington CM. Fast Track in the emergency department: a one-year experience with nurse practitioners. *Journal of Emergency Medicine.* 1992;10(3):367-373.

For peer review only

265. Wu FM, Rubenstein LV, Yoon J. Team functioning as a predictor of patient outcomes in early medical home implementation. *Health care management review.* 2018;43(3):238-248.
266. Xin H, Kilgore ML, Menachemi N, Sen B. The relationships between access to and use of a patient-centered medical home and healthcare utilization and costs: A cohort study using Medical Expenditure Panel Survey data from 2007 to 2010. *Health Services Management Research.* 2014;27(3-4):70-81.
267. Yuan X, Guh S, Hochheiser D, Mayo T, Nigam S. Incremental effect of quality blue practices on disease management in Louisiana. *Value in Health.* 2018;21(Supplement 1):S136-S137.
268. Zager K, Taylor YJ. Discharge to medical home: A new care delivery model to treat non-urgent cases in a rural emergency department. *Healthcare.* 2018;21:21.

For peer review only

Appendix Table 5: Countries in which studies were conducted

Countries	n (N=268)	Percentage (%)
**USA**	126	47
**UK**	43	16
**Canada**	30	11.2
**Australia**	20	7.5
**Netherlands**	13	4.8
**Switzerland**	5	1.9
**Sweden**	4	1.5
**France**	4	1.5
**Italy**	3	1
**South Korea**	3	1
**China**	3	1
**New Zealand**	2	0.8
**Saudi Arabia**	2	0.8
**Taiwan**	2	0.8
**Belgium**	1	0.4
**Brazil**	1	0.4
**Finland**	1	0.4
**Oman**	1	0.4
**Portugal**	1	0.4
**Spain**	1	0.4
**Singapore**	1	0.4
**Not reported**	1	0.4

For peer review only

Appendix Table 6: Types of interventions reported by the included studies

Within ED<br>Interventions or strategies	Outside ED<br>Interventions or strategies
**1. Area within ED staffed by PHCPs - to manage ED patients streamlined at triage**	**1. Improving timely access to primary care**
Minor injuries unit – NP assess, diagnose, treat, and discharge within agreed set of protocols	GP Cooperatives able to request X-rays in the evenings
Low acuity 4-bed ED triage area	GP Cooperatives - re-organised 100 GPs in the region and centralised the location for out-of-hours primary health care in one centrally located practice
NP in ED treat low acuity patients	Increasing number of GPs per 1000 patients
NP in fast track area	After-hour primary care practices (12 or more evening hours per week)
Rapid Medical Assessment – NP assess, diagnose, discharge low-acuity patients	High team functioning of primary care practices
**2. PHCPs located next to ED (sharing triage with ED) to manage ED patients streamed at triage and self-directed patients**	7-day opening of GP practices
The Emergency Care Access Point (ECAP) - a collaboration between a GP cooperative (GPC) and the ED	Transitioning low-risk cancer survivors back to their primary care provider (PCP) to decrease ED utilization
Walk-in centers co-located with ED	Advanced access to primary care (provide timely access to scheduled appointments, accommodate requests for same day access, understand facilitators and barriers to promoting uptake)
**3. PHCPs located at ED triage to manage patients**	High primary care access (95-100%)- ability for patients to schedule GP appointments
Advanced nurse practitioners-led rapid assessment triage (RAT)	Shared medical appointments (SMAs) - health care providers care for multiple patients with similar

For peer review only

	health conditions in a supportive, educational environment
Combined nurse and provider at triage	Los Angeles County Department of Health Services (LACDHS) - restructuring to improve access to and efficiency for providing ambulatory care and preventive services to Medicaid and uninsured populations
Paediatric "see-and-treat" service	Telephone-first consultation system - GPs were available for telephone consultations
Diversion of non-urgent patients to primary care	Same-day physician access in long-term care homes
Advanced practice providers (NP) in triage	**2. Integrating hospital and GP patient care**
Discharge to primary care with same day appointment	Hospital integrated general practice for emergency care services (HGP) (based on a team of GPs and emergency staff physicians) - HGPs are located within the hospital, sharing the same access point as the ED, as well as some infrastructure
Triage nurse order set	Evening clinics and walk-in centers within hospital campus located fairly close to, but outside ED
ED triage nurses applying Ottawa ankle, foot and knee rules	Hospital associated primary care unit with GPs (H-GP-unit as a fast track)
Split flow model of care combined with provider in triage	Primary care visits and clinic visits for all medical and surgical sub-specialities
Brief screening exam and tests order by triage nurse	Integrated emergency posts (IEPs) - integrating the care provided by A&E departments and GP posts
Triage nurse Renal Colic Fast Track pathway	Co-located GP clinics - low acuity patients are diverted from the ED to attend these clinics
Full triage Standing Orders	Emergency Care Access Point's (ECAP) - EDs and GPs work together. Triage decides if the patient will be seen by a GP or in the ED. To redirect non-urgent

For peer review only

	self-referrals to GP. GP functions as a gatekeeper for ED visits.
Nurse triage order to initiate the CT and NG rapid testing	Urgent Care Collaborations (UCCs) - out of hours GP sharing one combined entrance and joint triage with the ED
Nurse-initiated analgesia protocol	Integrated GP cooperatives - GP cooperative is located at the site of the hospital ED to ensure self-referred patients cannot enter ED without first having been seen by a GP
Nurse-initiated chest pain protocol	Integrating Primary Care Into Community Mental Health Centers
Triage Nurse Counselling	Primary Care Clinic Co-Located and Integrated in a Mental Health Setting
Nurse-initiated suspected fracture protocol	Mobile unit - located outside the main ED staffed by a registered nurse, a NP and a technician who floats between the unit and the main ED with an ED physician available for consultation as needed.
Nurse-initiated vaginal bleeding during pregnancy protocol	Primary Care Hub - Patients presenting to ED are streamed to the hub provided they do not meet any of the set exclusion criteria.
Nurse-initiated lower abdominal pain protocol	**3. Providing financial support**
Nurse-initiated upper abdominal pain	Adult walk-in primary care clinic on-campus, free of cost for uninsured patients under 200% of poverty and to actively urge insured ED high-users to either acquire a primary care physician if they did not have one, or regularly visit their existing PCP
Triage nurse-initiated X-ray	Citicare program - The GP (prospective capitation) was required to be available or provide physician coverage 24 hours a day, seven days a week, and functioned as a gatekeeper to all medical services

For peer review only

See-and-treat minor injury illnesses and injuries (NP + physician)	Primary care provider blended fee-for-service
**4. PHCPs fully integrated with the ED to manage ED patients**	**4. Implementing new clinics/services**
NP interpret X-rays	Patient centered medical home model – Team based primary care delivery and coordination with providers across the healthcare system
Asthma management for pediatric patients by advanced practice providers in ED	Primary health care provided by Nurse Practitioner - in centers for Medicare and Medicaid Services
GP added to the ED	GPs affiliated with newly established medical homes (MHs)
NP added to the ED staff during busy hours	Milwaukee Cares - provided uninsured patients with insurance and connected patients with free primary care
Registered nurse advises on appropriate care sources for non-urgent patients	Federally Qualify Health Centers (FQHC)- low acuity patients were scheduled a same day clinic appointment with a local FQHC that functions as "safety net" providers
Extended hours mental health liaison nurse (MHLN) service	Community Health Centers in rural counties in Georgia - access to a primary care medical home for the uninsured in rural communities with no other primary care safety net to potentially reduce uninsured ED visits
Pediatric trauma NP care	Clinic-based ambulatory case management intervention (Primary Intensive Care) by NP, to reduce hospital utilization
Nurse-led Chest pain service to risk-stratify	After-hours clinic - an appointment-only clinic where patients must contact their primary care physician or the children's hospital's Pediatric Health Information

For peer review only

	Line (telephone triage) to be scheduled for same-day visits
NP-led extended hours mental health liaison nurse (MHLN) service embedded within the ED	GP-led walk-in centres
Public education campaign within ED to patients on proper use of Eds and directing them to appropriate care	Primary Behavioral Health Care Integration (PBHCI) program – Physical and mental health clinics integrated
Chronic pain protocol - family physician and ED physician	Family Medicine Groups (FMG) - intended to include six to ten physicians and two nurses to serve a population of 10,000 to 20,000 patients, longer hours (weekdays, weekends, and holidays) and on-call services for vulnerable patients
Pediatric flow nurse - collaborates with ED nursing and medical staff to start treating patients and to help move children from the ED to the paediatric emergency short stay unit	Computerised Spanish guideline on the management of asthma (GEMA) in 7 GP clinics and a collaborative care environment between GP and Pulmonology department
Nurse triage and GP-led emergency service	Easy Breathing is an asthma management program for primary care clinicians
Transitional emergency nurse practitioner model	ED physicians have the option of scheduling follow-up appointments in a GP clinic
Geriatric Emergency Department Intervention (GEDI) model of service – NP In consultation, with the ED physician will undertake targeted geriatric assessment and problem formulation. They will then work with the primary ED team to fast-track diagnostic process and decision-making	Free clinic - offers general medical care, pregnancy testing, gynecology/birth control, immunizations, chronic disease management, and links to medication assistance
	Large primary-care based accountable care organizations (ACO) (both a Medicare and private insurer-sponsored plan)
	Internal Medicine Associates- Preventable Admissions Care Team (IMA/PACT) primary care clinic

For peer review only

	Asthma clinical pathway intervention - Each GP was given a 2-h group education session (algorithms for management of acute and chronic asthma)
	MD-Value in Prevention (MDVIP) - personalized preventive health care via annual 60- to 90-minute appointment that includes health screenings, diagnostics, personalized coaching and online tools for nutrition and exercise for a membership fee
	Population-tailored primary care clinic - hospital-based, open-access care model with nurse case management and wraparound onsite services
	The Geriatric Speciality Clinic (GSC) - interdisciplinary primary care geriatric practice with both NPs and geriatricians as primary care providers
	Extending CareFirst's Medical Home Program - nurses work with patients' usual primary care practitioners to coordinate care for high-risk Medicare patients
	WellMed - Each patient has a primary care clinician who coordinates all care and refers to specialists as needed
	Pediatric asthma centre - Patients were seen by either physicians or ANP (Advanced Practice Nurse)
	Optum Care Plus - Program that provides onsite care to nursing homes residents along with the resident's primary care physician and nursing home staff
	Medical Home practices - GP affiliated with the newly established medical homes
	Intensive primary care intervention - The intervention involved close follow-up by a nurse or a primary care

For peer review only

	physician beginning before discharge and continuing for the next 6 months.
	Center for Autism Services and Transition (CAST) - primary care center designed for adults with autism
	Family Health Units (FHU)
	Rural Health Clinics - located in a rural health professional shortage area and provide primary care and laboratory services
	Refugee Health Collaborative - a Collaborative-developed novel algorithm guided the process by which refugees establish care in Patient centered medical homes
	Team-based primary care and care coordination for high need high cost patients from academic safety net practices
	Children's and Young People's Ambulatory Unit (CAU) - ambulatory care model with limited opening hours
	Pediatric primary care practices
	Coordinated Care Center (CCC) - An intensive primary care clinic (NPs and nurses), created to address the unique needs of high-needs and high-cost patients

For peer review only

Preferred Reporting Items for Systematic reviews and Meta-Analyses extension for Scoping Reviews (PRISMA-ScR) Checklist

SECTION	ITEM	PRISMA-ScR CHECKLIST ITEM	REPORTED ON PAGE #
**TITLE**			
Title	1	Identify the report as a scoping review.	
**ABSTRACT**			
Structured summary	2	Provide a structured summary that includes (as applicable): background, objectives, eligibility criteria, sources of evidence, charting methods, results, and conclusions that relate to the review questions and objectives.	
**INTRODUCTION**			
Rationale	3	Describe the rationale for the review in the context of what is already known. Explain why the review questions/objectives lend themselves to a scoping review approach.	
Objectives	4	Provide an explicit statement of the questions and objectives being addressed with reference to their key elements (e.g., population or participants, concepts, and context) or other relevant key elements used to conceptualize the review questions and/or objectives.	
**METHODS**			
Protocol and registration	5	Indicate whether a review protocol exists; state if and where it can be accessed (e.g., a Web address); and if available, provide registration information, including the registration number.	
Eligibility criteria	6	Specify characteristics of the sources of evidence used as eligibility criteria (e.g., years considered, language, and publication status), and provide a rationale.	
Information sources*	7	Describe all information sources in the search (e.g., databases with dates of coverage and contact with authors to identify additional sources), as well as the date the most recent search was executed.	
Search	8	Present the full electronic search strategy for at least 1 database, including any limits used, such that it could be repeated.	
Selection of sources of evidence†	9	State the process for selecting sources of evidence (i.e., screening and eligibility) included in the scoping review.	
Data charting process‡	10	Describe the methods of charting data from the included sources of evidence (e.g., calibrated forms or forms that have been tested by the team before their use, and whether data charting was done independently or in duplicate) and any processes for obtaining and confirming data from investigators.	
Data items	11	List and define all variables for which data were sought and any assumptions and simplifications made.	
Critical appraisal of individual sources of evidence§	12	If done, provide a rationale for conducting a critical appraisal of included sources of evidence; describe the methods used and how this information was used in any data synthesis (if appropriate).	
Synthesis of results	13	Describe the methods of handling and summarizing the data that were charted.	

For peer review only - http://bmjopen.bmj.com/site/about/guidelines.xhtml

SECTION	ITEM	PRISMA-ScR CHECKLIST ITEM	REPORTED ON PAGE #
**RESULTS**			
Selection of sources of evidence	14	Give numbers of sources of evidence screened, assessed for eligibility, and included in the review, with reasons for exclusions at each stage, ideally using a flow diagram.	
Characteristics of sources of evidence	15	For each source of evidence, present characteristics for which data were charted and provide the citations.	
Critical appraisal within sources of evidence	16	If done, present data on critical appraisal of included sources of evidence (see item 12).	
Results of individual sources of evidence	17	For each included source of evidence, present the relevant data that were charted that relate to the review questions and objectives.	
Synthesis of results	18	Summarize and/or present the charting results as they relate to the review questions and objectives.	
**DISCUSSION**			
Summary of evidence	19	Summarize the main results (including an overview of concepts, themes, and types of evidence available), link to the review questions and objectives, and consider the relevance to key groups.	
Limitations	20	Discuss the limitations of the scoping review process.	
Conclusions	21	Provide a general interpretation of the results with respect to the review questions and objectives, as well as potential implications and/or next steps.	
**FUNDING**			
Funding	22	Describe sources of funding for the included sources of evidence, as well as sources of funding for the scoping review. Describe the role of the funders of the scoping review.	

JBI = Joanna Briggs Institute; PRISMA-ScR = Preferred Reporting Items for Systematic reviews and Meta-Analyses extension for Scoping Reviews.
* Where *sources of evidence* (see second footnote) are compiled from, such as bibliographic databases, social media platforms, and Web sites.
† A more inclusive/heterogeneous term used to account for the different types of evidence or data sources (e.g., quantitative and/or qualitative research, expert opinion, and policy documents) that may be eligible in a scoping review as opposed to only studies. This is not to be confused with *information sources* (see first footnote).
‡ The frameworks by Arksey and O'Malley (6) and Levac and colleagues (7) and the JBI guidance (4, 5) refer to the process of data extraction in a scoping review as data charting.
§ The process of systematically examining research evidence to assess its validity, results, and relevance before using it to inform a decision. This term is used for items 12 and 19 instead of "risk of bias" (which is more applicable to systematic reviews of interventions) to include and acknowledge the various sources of evidence that may be used in a scoping review (e.g., quantitative and/or qualitative research, expert opinion, and policy document).

From: Tricco AC, Lillie E, Zarin W, O'Brien KK, Colquhoun H, Levac D, et al. PRISMA Extension for Scoping Reviews (PRISMAScR): Checklist and Explanation. Ann Intern Med. 2018;169:467–473. doi: 10.7326/M18-0850.

BMJ Open

Interventions and strategies involving primary healthcare professionals to manage emergency department overcrowding: a scoping review

Journal:	*BMJ Open*
Manuscript ID	bmjopen-2021-048613.R1
Article Type:	Original research
Date Submitted by the Author:	01-Apr-2021
Complete List of Authors:	Jeyaraman, Maya; University of Manitoba, George and Fay Yee Center for Healthcare Innovation<br>Copstein, Leslie; University of Manitoba, George & Fay Yee Center for Healthcare Innovation<br>Al-Yousif, Nameer; University of Manitoba, George & Fay Yee Center for Healthcare Innovation<br>Alder, Rachel; University of Manitoba, Max Rady College of Medicine, Rady Faculty of Health Sciences,<br>Kirkland, Scott ; University of Alberta, Department of Emergency Medicine, Faculty of Medicine & Dentistry,<br>Al-Yousif, Yahya; University of Manitoba, George & Fay Yee Center for Healthcare Innovation<br>Suss, Roger; University of Manitoba, Department of Family Medicine, Rady Faculty of Health Sciences,<br>Zarychanski, Ryan ; CancerCare Manitoba, Department of Medical Oncology and Hematology<br>Doupe, Malcolm; University of Manitoba, Department of Community Health Sciences, Rady Faculty of Health Sciences,<br>Berthelot, Simon; Centre de recherche du CHU de Québec-Université Laval, Axe Santé des populations et Pratiques optimales en santé,<br>Mireault, Jean; Université de Montréal, HEC Pôle santé,<br>Tardif, Patrick; Department of Emergency Medicine, Cité de la santé de Laval,<br>Askin, Nicole; University of Manitoba, WRHA Virtual Library,<br>Buchel, Tamara; Manitoba College of Family Physicians<br>Rabbani, Rasheda; University of Manitoba, George and Fay Yee Center for Healthcare Innovation,<br>Beaudry, Thomas; George & Fay Yee Center for Healthcare Innovation, Patient and Public Engagement Collaborative Partnership,<br>Hartwell, Melissa; The Alberta Primary and Integrated Health care Innovation Network<br>Shimmin, Carolyn; University of Manitoba, George and Fay Yee Center for Healthcare Innovation,<br>Edwards, Jeanette; Shared Health Manitoba, Community Health Quality and Learning<br>Halas , Gayle; Manitoba Primary and Integrated Health care Innovation Network<br>Sevcik, William; University of Alberta, Department of Emergency Medicine, Faculty of Medicine & Dentistry,

	Tricco, Andrea; St Michael's Hospital Li Ka Shing Knowledge Institute, Knowledge Translation Program, Unity Health Toronto,<br>Chochinov, Alecs; University of Manitoba, Department of Emergency Medicine, Faculty of Medicine,<br>Rowe, Brian; University of Alberta, Department of Emergency Medicine, Faculty of Medicine & Dentistry and School of Public Health,<br>Abou-Setta, Ahmed; University of Manitoba, George and Fay Yee Center for Healthcare Innovation
**Primary Subject Heading**:	Emergency medicine
Secondary Subject Heading:	Public health
Keywords:	ACCIDENT & EMERGENCY MEDICINE, PRIMARY CARE, PUBLIC HEALTH

SCHOLARONE™
Manuscripts

I, the Submitting Author has the right to grant and does grant on behalf of all authors of the Work (as defined in the below author licence), an exclusive licence and/or a non-exclusive licence for contributions from authors who are: i) UK Crown employees; ii) where BMJ has agreed a CC-BY licence shall apply, and/or iii) in accordance with the terms applicable for US Federal Government officers or employees acting as part of their official duties; on a worldwide, perpetual, irrevocable, royalty-free basis to BMJ Publishing Group Ltd ("BMJ") its licensees and where the relevant Journal is co-owned by BMJ to the co-owners of the Journal, to publish the Work in this journal and any other BMJ products and to exploit all rights, as set out in our licence.

The Submitting Author accepts and understands that any supply made under these terms is made by BMJ to the Submitting Author unless you are acting as an employee on behalf of your employer or a postgraduate student of an affiliated institution which is paying any applicable article publishing charge ("APC") for Open Access articles. Where the Submitting Author wishes to make the Work available on an Open Access basis (and intends to pay the relevant APC), the terms of reuse of such Open Access shall be governed by a Creative Commons licence – details of these licences and which Creative Commons licence will apply to this Work are set out in our licence referred to above.

Other than as permitted in any relevant BMJ Author's Self Archiving Policies, I confirm this Work has not been accepted for publication elsewhere, is not being considered for publication elsewhere and does not duplicate material already published. I confirm all authors consent to publication of this Work and authorise the granting of this licence.

For peer review only

Interventions and strategies involving primary healthcare professionals to manage emergency department overcrowding: a scoping review

Review Authors: Maya M. Jeyaraman[1], Leslie Copstein[1], Nameer Al-Yousif[1], Rachel N. Alder[2] Scott W. Kirkland[3], Yahya Al-Yousif[1], Roger Suss[4], Ryan Zarychanski[5], Malcolm Doupe[6], Simon Berthelot[7], Jean Mireault[8], Patrick Tardif[9], Nicole Askin[10], Tamara Buchel[11], Rasheda Rabbani[1], Thomas Beaudry[12], Melissa Hartwell[13], Carolyn Shimmin[1], Jeanette Edwards[14], Gayle Halas[15], William Sevcik[3], Andrea C. Tricco[16], Alecs Chochinov[17], Brian H. Rowe[3,18], Ahmed M. Abou-Setta[1]

Affiliations:

[1]George & Fay Yee Center for Healthcare Innovation, University of Manitoba, Winnipeg, Manitoba, Canada

[2] Max Rady College of Medicine, Rady Faculty of Health Sciences, University of Manitoba, Winnipeg, Manitoba, Canada

[3] Department of Emergency Medicine, Faculty of Medicine & Dentistry, University of Alberta, Edmonton, Alberta, Canada

[4] Department of Family Medicine, Rady Faculty of Health Sciences, University of Manitoba, Winnipeg, Manitoba, Canada

[5] Department of Medical Oncology and Hematology, Cancer Care Manitoba, Winnipeg, Manitoba, Canada

[6] Department of Community Health Sciences, University of Manitoba, Winnipeg, Manitoba, Canada

[7] Centre de recherche du CHU de Québec-Université Laval, Axe Santé des populations et Pratiques optimales en santé, Laval, Québec, Canada.

[8] HEC Pôle santé, Université de Montréal, Montreal, Quebec, Canada

[9] Department of Emergency Medicine, Cité de la santé de Laval, Laval, Quebec, Canada

[10] WRHA Virtual Library, University of Manitoba, Winnipeg, Manitoba, Canada

[11] Manitoba College of Family Physicians, Winnipeg, Manitoba. Canada

[12] Patient and Public Engagement Collaborative Partnership, George & Fay Yee Center for Healthcare Innovation, Winnipeg, Manitoba, Canada

For peer review only

For peer review only - http://bmjopen.bmj.com/site/about/guidelines.xhtml

[13] The Alberta Primary and Integrated Health care Innovation Network, Edmonton, Alberta, Canada

[14] Community Health Quality and Learning, Shared Health Manitoba, Winnipeg, Manitoba, Canada

[15] Manitoba Primary and Integrated Health care Innovation Network, Winnipeg, Manitoba, Canada

[16] Knowledge Translation Program, St. Michael's Hospital Li Ka Shing Knowledge Institute, Unity Health Toronto, Toronto, Ontario, Canada

[17] Department of Emergency Medicine, Faculty of Medicine, University of Manitoba, Winnipeg, Manitoba, Canada

[18] School of Public Health, University of Alberta, Edmonton, Alberta, Canada.

Corresponding author: Maya Jeyaraman, MD PhD
George & Fay Yee Center for Healthcare Innovation
753 Mc Dermot Avenue, Winnipeg, MB, R3E 0T6, Canada
Email: maya.jeyaraman@umanitoba.ca
Phone: (204) 594-5362; Fax: (204) 594-5394.

Running Title: Interventions involving primary healthcare professionals to manage emergency department overcrowding

Keywords: emergency department overcrowding, primary healthcare professionals, patient engagement

Total word count: 3696 words; **Abstract**: 279 words**; Figures**: 2; **Total tables**: 9 (Main tables: 1; Appendix tables: 8)

For peer review only

Abstract

Objectives: To conduct a scoping review to identify and summarize the existing literature on interventions involving primary healthcare professionals to manage emergency department overcrowding

Design: Scoping review

Data sources: A comprehensive database search of Medline (Ovid), EMBASE (Ovid), Cochrane Library (Wiley), and CINAHL (EBSCO)) databases was conducted (inception until January 2020) using peer-reviewed search strategies, complemented by a search of grey literature sources.

Eligibility criteria: Interventions and strategies involving primary healthcare professionals (PHCPs: general practitioners [GP], nurse practitioners [NP], or nurses with expanded role) to manage emergency department (ED) overcrowding.

Methods: We engaged and collaborated, with 13 patient partners during the design and conduct stages of this review. We conducted this review using the JBI guidelines. Two reviewers independently selected studies and extracted data. We conducted descriptive analysis of the included studies (frequencies and percentages).

Results: From 23,947 records identified, we included 268 studies published between 1981 to 2020. The majority (58%) of studies were conducted in North America and were predominantly cohort studies (42%). The reported interventions were either "within ED" (48%) interventions (e.g., PHCP-led ED triage or fast track), or "outside ED" (52%) interventions (e.g., after-hours GP clinic, GP cooperatives). PHCPs involved in the interventions were: GP (32%), NP (26%), nurses with expanded role (16%) and combinations of the PHCPs (42%). The "within ED" and "outside ED" interventions reported outcomes on patient flow and ED utilization, respectively.

For peer review only

For peer review only - http://bmjopen.bmj.com/site/about/guidelines.xhtml

Conclusions: We identified many interventions involving PHCPs that predominantly reported a positive impact on ED utilization/ patient flow metrics. Future research needs to focus on conducting well-designed RCTs and systematic reviews to evaluate the effectiveness of specific interventions involving PHCPs to critically appraise and summarize evidence on this topic.

For peer review only

For peer review only - http://bmjopen.bmj.com/site/about/guidelines.xhtml

Article Summary

Strengths and limitations of this study

- A major strength of our study is that we collaborated with twelve patient partners during the design, conduct and dissemination stages of this scoping review to refine the review question, identify patient-important review outcomes, develop search terms, grey literature search and knowledge dissemination.
- This comprehensive scoping review was conducted using JBI guidelines and an *a priori* registered protocol without any restrictions on publication dates, population, or study design
- A limitation of this scoping review is that we only included English language publications

For peer review only

For peer review only - http://bmjopen.bmj.com/site/about/guidelines.xhtml

INTRODUCTION

Emergency department (ED) overcrowding is an increasing global crisis,[1] leading to challenges in the delivery of timely healthcare to patients, which can result in adverse patient outcomes.[2,3] Overcrowding occurs when the demand for services exceeds the ability of the ED to provide quality care within acceptable time frames.[4] Studies have shown how ED crowding compromises patient safety and contributes to poor quality of care, such as delays in antibiotic treatment,[5] poor pain management for patients with severe pain,[6] increased patient mortality,[7] and staff frustration.[8,9]

The problem of ED overcrowding is complex and challenging, and models have been proposed to characterize it using input (e.g., health lines, patient volume, diversion strategies), throughput (e.g., provider availability, diagnostic testing, response to therapy), output (e.g., consultation times, ED boarding and lack of hospital beds), and system-wide (e.g., remuneration, pay-for performance, public reporting, accountability frameworks) factors.[10] One such input factor impacting ED overcrowding is the large volume of low acuity patient visits to the ED, which can stress the available resources in the ED, leading to decreased patient and staff satisfaction.[11,12]

A lack of access to a primary care provider (PCP),[12-14] as well as an inability to see their PCP after-hours,[15] or to get an appointment within an appropriate time frame[3,15,16] can often force patients with low acuity conditions to turn to ED as their last resort.[17] Some have suggested that many of these presentations represent primary care sentinel conditions (e.g., asthma, hypertension, low back pain) which could be better managed through greater continuity and ongoing follow-up from primary care providers.[18] In many rural communities, EDs are often the only source of primary care.[4]

For peer review only - http://bmjopen.bmj.com/site/about/guidelines.xhtml

As such, it is crucial to identify interventions and strategies involving primary healthcare professionals (PHCP) that have an impact on the ED metrics. Using a scoping review methodology, in order to map the existing literature on this topic, the main objective was to identify and summarize existing literature on the interventions and strategies involving PHCPs (family physicians/general practitioners (GP), nurse practitioners (NP), or nurses with expanded role) to manage ED overcrowding.

For peer review only

For peer review only - http://bmjopen.bmj.com/site/about/guidelines.xhtml

METHODS

An *a priori* protocol was developed and posted on the Open Science Framework platform.[19] This scoping review was conducted using the methodologically rigorous JBI Guideline for scoping reviews,[20] and the six-stage methodological framework outlined by Levac et al[21]. This scoping review adheres to the reporting guidelines of the Preferred Reporting Items for Systematic reviews and Meta-Analyses extension for scoping reviews (PRISMA-ScR).[22,23]

Population, Concept and Context (PCC)

Studies were included in the review if they investigated an intervention or strategy to manage ED overcrowding and ED patient flow and involved a primary healthcare professional (PHCP) including family physician/general practitioner (GP), nurse practitioner (NP) or nurse with increased authority. Editorials, commentaries, reviews, and historical articles were excluded from the review. For feasibility, only English language publications were included. Additional details regarding study eligibility criteria (Population, Concept and Context) are available in ***Appendix Table 1***.

Search methods for identifying relevant citations

A health librarian (TR) with experience conducting systematic literature searches designed a comprehensive search strategy for Medline (Ovid) to identify literature relevant to the objectives (***Appendix Table 2***). After feedback from the principal investigators and the patient partners, the Medline search was then peer reviewed by an independent librarian (JJ) using the Peer Review of Electronic Search Strategies (PRESS) checklist.[24] Once finalized, it was adapted for use in the following three additional electronic databases: EMBASE (Ovid), Cochrane Library (Wiley), and

For peer review only - http://bmjopen.bmj.com/site/about/guidelines.xhtml

CINAHL (EBSCO)). The databases were searched from their inception to January 2020 by an experienced librarian (NA). A search of several grey literature sources (***Appendix Table 3)*** was conducted to identify additional publications. Reference lists of all the included publications were searched for additional relevant studies. References were imported and managed in EndNote™ (Version X8, Thomson Reuters, New York, NY, USA).

Selection of sources of evidence

Two reviewers (NAY & (LC, YAY or RA)) independently reviewed and screened the titles and abstracts of all citations identified by our search strategy using standardized pilot-tested (n=10) screening forms. Citations identified as potentially relevant, or which the reviewers were unclear of its inclusion were eligible for full-text screening. Two reviewers (NAY & (LC or YAY)) independently reviewed full texts of all included studies and discrepancies were resolved via consensus or a third-party adjudicator (MJ).

Charting information, risk of bias assessment and synthesis of results

A standardized data extraction form was developed, and pilot tested (n=10) independently by reviewers in the team. As a part of data extraction, two reviewers (LC & (NAY, YAY or RA)) independently extracted data from the included studies. When required, the form was modified, using an iterative consensus-based process[25]. All disagreements among reviewers were resolved by consensus and checked for accuracy by a third reviewer (SK and/or MJ). Extracted data included: study identification, author(s), publication year, publication type, study design, country of origin, type of ED (urban versus rural; adult, pediatric, or mixed), type of intervention or strategy (within ED or outside ED), type of PHCP (GP, NP or nurse with increased authority), nature of

For peer review only - http://bmjopen.bmj.com/site/about/guidelines.xhtml

intervention or strategy (added a PHCP, or increased responsibility to a PHCP), and the nature of impact of interventions on ED related outcomes as reported by the study authors in the included studies (positive impact, negative impact, or no impact).

Consistent with established scoping review methodology,[25,26] we did not appraise the risk of bias of the included studies, nor did we summarize the data quantitatively (meta-analyses). Instead, we synthesized the findings using descriptive statistical analyses (e.g., frequencies and percentages) of the extracted variables, and reported using graphs and tables.

Patient and public involvement (Collaboration with stakeholders)

We collaborated with a diverse group of 13 patient partners (self-identified as Indigenous, Immigrant, White and/or living with disability) identified with the help of Strategy for Patient-Oriented Research (SPOR) Support for People and Patient-Oriented Research and Trials units (Manitoba, Alberta, and Quebec), during the design phase, grant application phase and in the conduct of this review. The patient engagement process has been reported according to the GRIPP2 checklist (short form).[27] During the design phase, 12 patient partners collaborated with the principle investigators (12 one-on-one interviews followed by discussion group to arrive at a consensus) to refine the review question to focus on interventions involving PHCPs, to finalize review inclusion criteria, and to select patient-important outcomes. During the grant application phase, they supported our grant application as knowledge users and provided letters of support. While conducting this scoping review, 3 patient partners collaborated with researchers in refining the search strategy, reviewing the included studies, identifying grey literature, interpreting study results (we shared knowledge of our study findings and integrated information obtained from them

For peer review only - http://bmjopen.bmj.com/site/about/guidelines.xhtml

in the reporting of the study findings and future directions), and in the knowledge dissemination process (conference presentation). Patient partners were compensated for their time.

Our team members (researchers and patient partners) from multiple provinces across Canada, and the partner organizations provided ongoing feedback. Three presentations were made to various members of the team during the conduct of this review by the principal investigator to discuss the extracted data, obtain feedback regarding the inclusion and classification of various identified interventions and strategies, as well as review outcomes of importance, and to discuss the future directions of the project (discussions regarding patient-important research priorities and review questions for future systematic reviews). Partnering organizations included Manitoba Medical Service Foundation (MMSF), SPOR SUPPORT units (Manitoba, Alberta, and Quebec), Emergency Medicine Research Group (EMeRG), and Primary and Integrated Healthcare Innovation network (PIHCI), and the knowledge users (Winnipeg Regional Health Authority, Emergency Strategic Clinical Network, Shared Health Manitoba, and Manitoba College of Family Physicians).

For peer review only - http://bmjopen.bmj.com/site/about/guidelines.xhtml

RESULTS

Our literature search identified 23,947 records from the initial database search, of which 268 studies (274 reports) met our inclusion criteria. A detailed description of the study selection process for this scoping review is depicted using the PRISMA study flow diagram for scoping reviews[22,28] (***Figure 1***).

Main Results

Study characteristics

The included studies (***Appendix Table 4***) were published between 1981 and 2020 with the majority of studies published in the last 10 years (2010-2020), showing an upward trend in the number of publications per year over time (1981-2020; ***Figure 2***). For details regarding the key characteristics of the included studies, see ***Table 1 & Appendix Table 5***. The included studies were mostly cohort (prospective and retrospective) studies (41.8%) from North America (58%) and Europe (28.4%) and published in peer-reviewed journals (76.9%). Nearly half of the included studies were conducted in USA (47%) and only 8.2% were randomized controlled trials. The ED location was most often urban (n = 137, 51%) and 16 studies (6%) reported an ED location that provided care to patients from rural and urban areas. Most of the studies did not specify the type of ED (n = 146, 54.5%); however, mixed (n = 79, 29.5%), adult (n = 19, 7%), or pediatric (n = 24, 9%) ED types were documented.

Interventions and Strategies involving PHCPs

For this review, interventions or strategies involving PHCPs were categorized as either "within ED" or "outside ED" interventions. A total of 139 studies (52%) reported "outside ED"

For peer review only - http://bmjopen.bmj.com/site/about/guidelines.xhtml

interventions or strategies, and 129 studies (48%) reported "within ED" interventions or strategies (***Appendix Table 6)***. The study intervention for each of the 268 included studies has been reported in ***Appendix Table*** *7*.

Several "within ED" interventions and strategies identified in this review. These interventions were grouped under four common themes as follows: (1) Area within ED staffed by PHCPs to manage lower acuity ED patients streamlined at triage[29]; (2) PHCPs located next to ED (sharing common triage with ED) to manage lower acuity ED patients streamed at triage as well as self-directed patients[30], (3) PHCPs located at ED triage[31] to manage lower acuity ED patients, and (4) PHCPs fully integrated within the ED to manage ED patients along with the ED team[32] (***Appendix Table 6)***.

The PHCP triage intervention involved patient management strategies such as, "see and treat" lower acuity patients,[31] diverting low acuity patients to adjacent/co-located primary care center or after-hours primary care center,[33] or the triage nurse was given increased authority to order diagnostic investigations or initiate a specified protocol.[34] A few "within ED" interventions involved low acuity patients streamlined at triage to a PHCP working alone within ED (for example rapid medical assessment units,[35] fast-track units,[36] or Emergency care access points[35]) for management. Other "within ED" interventions (non- triage) involved PHCPs working in conjunction with the emergency physician and the rest of the ED team in the interpretation of diagnostic imaging, management of lower acuity or in some cases higher acuity patients, and in discharge process.[32]

Studies employed several "outside ED" interventions or strategies. We identified four common themes across these interventions and grouped them under these following themes: (1) improving timely access to primary care, (2) integrating hospital and GP care, (3) providing

For peer review only - http://bmjopen.bmj.com/site/about/guidelines.xhtml

financial support, and (4) implementing new clinics/services (***Appendix Table 6)***. Some examples of the "outside ED" interventions or strategies were: increasing after-hours primary care,[37] free access to primary care for the uninsured,[38] adjacent or co-located primary care clinic for lower acuity patients,[39] introduction of a patient centered medical home that addresses primary care needs of patients,[40] implementation of GP co-operatives (out-of-hours primary health care in one centrally located practice),[41] urgent care collaborations between the GP and ED,[42] GP-led walk-in centers,[43] primary care provider blended fee for service,[44] hospital integrated general practice for emergency care services,[45] integrated emergency posts where care is provided by both ED and GPs,[46] rural health clinics,[47] and advanced access primary care with timely access.[48]

Involvement of primary healthcare providers

The type of PHCP involved in the interventions or strategies involving PHCPs were as follows: family physician (n = 85, 31.7%), nurse practitioner (n = 69, 25.7%), or nurse given increased authority (n = 43, 16%). There were also a number of dyads reported, including family physician and nurse practitioner (n = 15, 5.6%), family physician and nurse (n = 2, 0.8%), or nurse practitioner and nurse (n = 8, 3%). In some studies, the intervention or strategy was described as primary care with no mention of the specific type of PHCP involved (n = 46, 17.2%). The majority of studies introduced or added a PHCP (n = 190, 70.1%) as a part of the intervention or strategy, and the rest of the studies increased the responsibility of the PHCPs as a part of the intervention or strategy (n = 59, 22%). In a few studies (n = 19, 7%) it was not clear if there was an addition or increased responsibility of a PHCP.

Reported Impact of PHCP Interventions and Strategies on ED outcomes

For peer review only - http://bmjopen.bmj.com/site/about/guidelines.xhtml

Several ED-related outcomes, and the impact (positive, negative or no impact) of the PHCP interventions and strategies on these ED outcomes were reported by the included studies. The majority of the included studies that reported "outside ED" interventions or strategies investigated the impact of these interventions on "input" related outcomes (i.e., minimizing the ED utilization), whereas studies reporting "within ED" interventions or strategies investigated the impact of the interventions on "throughput" related outcomes (i.e., ED patient flow metrics). The majority of the included studies reported ED outcomes such as ED visits, ED LOS (length of stay), leave without being seen (LWBS), patient satisfaction, patient safety, ED workup time, time to provider initial assessment, number of patients diverted to primary care, and ED cost savings (***Appendix Table 8***).

The reported impact (positive impact, negative impact, or no impact) of various PHCP-led interventions and strategies on various ED outcomes is reported in ***Appendix Table 8***. About 62% of the included studies reported a positive impact on ED utilization (i.e., decrease in lower acuity ED visits), whereas 28% reported no impact and 9.7% reported negative impact (increased ED utilization). Similarly, the majority of the included studies reported a positive impact on many important ED related outcomes such as ED length of stay (73.6%), leave without being seen (77.1%), patient satisfaction (62.1%), time to provider initial assessment (84.6%), ED workup time (62.5%), leave against medical advice (50%), patient safety (100%), and leave before completion of service (50%). The rest of the included studies reported either a negative impact or no impact for the above outcomes.

For peer review only - http://bmjopen.bmj.com/site/about/guidelines.xhtml

DISCUSSION

This scoping review identified 268 unique comparative studies that reported on various interventions or strategies involving PHCPs to manage ED overcrowding and ED patient flow (regardless of the geographic area or the type of healthcare system). Most of the included studies were journal articles of cohort design, published in North America, and were conducted in urban ED's. The interventions or strategies involving PHCPs were implemented either "within ED" or "outside ED.

To the best of our knowledge, this is the first scoping review to describe various interventions or strategies involving PHCPs and their reported impact on ED outcomes. In this scoping review we identified and described various types of "within ED" (triage or non-triage) PHCP-led interventions from existing literature. In 2010, Carson et al[49] had described four models in which lower acuity patients could be managed within ED by PHCPs, as follows: (1) streaming ED patients at triage to an area within ED staffed by PHCPs, for management[49]; (2) PHCPs located next to ED (sharing triage with ED) for management of redirected low-acuity ED patients or self-directed patients[49]; (3) PHCPs located at triage who either "see and treat" or redirect low acuity patients to primary care[49]; (4) PHCPs fully integrated with the ED team supporting management of low acuity or higher acuity patients.[49] In this review, each of the "within ED" PHCP-led interventions reported by the included studies fit at least one of the four of the Carson's models. It is important to note that the PHCPs in these interventions were providing episodic care (episode related primary care) and not care continuity.

Although we are unaware of any previous scoping reviews on this topic, there are a few systematic reviews that have been previously published which found a positive impact of specific PHCP-led "within ED" interventions on ED patient flow metrics. In 2011, Rowe et al[50] reported

For peer review only - http://bmjopen.bmj.com/site/about/guidelines.xhtml

that triage nurse ordering (i.e. triage nurse given increased authority) had a positive impact on ED patient flow metrics Similarly, in another systematic review, Rowe et al[51] had reported a positive impact of triage liaison physicians on ED patient flow metrics. A more recent review reported that NPs practicing in the ED improved ED quality metrics and patient satisfaction [52], while another review investigating strategies to alleviate access block and overcrowding, suggested that co-locating primary care, fast-track and nurse practitioners within ED may have a positive impact.[53] Other reviews, however, have reported a limited impact of "within-ED" interventions on ED patient flow metrics. A previous Cochrane review,[54] which included only four studies, reported a very low certainty of evidence on the role of GP's and NP's within the ED (but not at triage) on ED flow metrics. In 2015, a narrative review[55] was published on the nurse practitioner role in the ED and reported that high quality research is required to demonstrate clinical and service effectiveness.

This review identified numerous types of "outside ED" interventions that have been employed across the literature. It is important to note that studies reporting "outside ED" interventions and strategies focused predominantly on reduction of ED utilization by patients and reported "ED visits" as an outcome (input related outcomes) in their study report. Although we are unaware of any scoping reviews on "outside ED" PHCP-led interventions, there have been a few focused systematic reviews published to date on specific areas under this broad topic. Ansell et al., 2017,[56] summarized interventions to reduce wait times for primary care appointments to mitigate ED utilization, and concluded that open access scheduling and patient-centered interventions may reduce wait times. Patients seeking ED care due to lack of timely primary care access (same-day or after-hours appointments) may contribute to ED overcrowding and in this context some of the

For peer review only - http://bmjopen.bmj.com/site/about/guidelines.xhtml

“outside ED” interventions summarized in this scoping review may be beneficial in mitigating ED overcrowding.

The results of this scoping review found that the majority of studies reported a positive impact of PHCP-led interventions and strategies on ED overcrowding and patient flow metrics. Although what may work in one context may not work in another due to many factors (e.g., funding, political views, type of healthcare systems, etc.) that may play a role in determining the best intervention or strategy for a specific context. It is important to conduct systematic reviews to critically appraise and summarize evidence on the effectiveness of specific “within ED” or “outside ED” interventions involving PHCPs to mitigate ED overcrowding. In this scoping review we only identified eight relevant RCTs that reported PHCP-led interventions. Thus, it is important to conduct well designed RCTs evaluating the effectiveness of specific interventions involving PHCPs on ED outcomes to generate high quality evidence on this topic.

This review has numerous strengths. To limit selection bias, multiple steps and multiple independent reviewers reviewed the studies to make selections in an unbiased manner. One of the major strengths of this scoping review is that we engaged and collaborated with twelve patient partners from multiple provinces across Canada during the design stage to refine our review question and identify patient-important review outcomes, as well as during the conduct stage and knowledge dissemination stages. In addition, the JBI guidelines were used for conducting this scoping review along with an *a priori* registered protocol. A comprehensive search strategy was developed by an experienced health librarian and peer-reviewed by a second information specialist using PRESS guidelines along with search for grey literature sources, to identify relevant studies to answer our review question. In addition, the search strategy was reviewed by our patient partners who collaborated on the project. We did not have any restrictions on publication dates, population,

For peer review only - http://bmjopen.bmj.com/site/about/guidelines.xhtml

or study design (with exception of narrative reviews, editorials, commentaries, and historical articles).

There are some important limitations with this review to be considered. In our review, publication bias was only partially addressed. Despite using a comprehensive and exhausting search strategy, we made a pragmatic decision to only include English language publications. Consequently, it is possible that some of the studies from countries where English is not the first language may have been missed. Our review focused on the interventions and strategies that involved only PHCPs such as family physicians, nurse practitioners and nurses with increased authority. Studies that involved ED physicians or specialists were not considered in this review for feasibility. We had also made an *a priori* decision, for feasibility, to not include studies that had exclusively investigated the role of physician assistants, but if a study reported them to be a part of a team that consisted of PHCPs, then we included those studies in our scoping review. We included studies that had involved any one of the included PHCPs individually or as a part of a team that had other professionals, and thus in these studies the impact of intervention cannot be solely ascribed to a specific PHCP.

Conclusions

This review provides a timely contribution to improving the understanding of the growing problem of ED overcrowding. In this scoping review we have identified a variety of interventions "outside" and "within" the ED involving PHCPs from the existing literature and we have summarized the nature of the impact of these interventions on "input" (ED utilization) and "throughput" (ED patient flow metrics) related outcomes. We have also identified and reported innovative ways in which PHCPs may be engaged in patient care. The geographic area in which

For peer review only - http://bmjopen.bmj.com/site/about/guidelines.xhtml

these studies were conducted, and the type of healthcare systems vary widely across the included studies and may influence how these results can be interpreted. In the future, it will be important for researchers to focus on conducting high quality systematic reviews to synthesize evidence on the effectiveness and unintended consequences of specific “within ED” or “outside ED” interventions involving PHCPs. It will also be important to engage diverse patient and caregiver perspectives in the systematic review process to align with patient-important priorities and key patient-identified outcomes prior to conducting the systematic reviews. Finally, researchers should be encouraged to conduct well-designed RCTs to generate high quality evidence on this topic, as there have been a limited number of RCTs conducted on this topic.

For peer review only

For peer review only - http://bmjopen.bmj.com/site/about/guidelines.xhtml

Acknowledgements: We are very grateful to the Canadian Institutes of Health Research (CIHR) (NKS 158643), Manitoba Medical Services Foundation (# 8-2018-06) and Winnipeg Foundation (# 8-2018-06) for providing financial support for this project. Dr. Rowe's research was supported by a Scientific Director's Grant (SOP 168483) from CIHR. Dr. Tricco's research is funded by a Tier 2 Canada Research Chair in Knowledge Synthesis. We thank the Emergency Medicine Research Group (EMeRG) in the Department of Emergency Medicine at the University of Alberta for in-kind resources. We are also very grateful to all the patient partners who collaborated with us in the design, conduct and dissemination stages of this project. We are thankful to Mr. Frank Krupka for the kind support of this project during his time as the Executive Director of CHI. We are very grateful to Dr. Cristina Villa-Roel for the kind support of this project. We thank Ms. Tamara Rader, Ms. Christine Nielson, and Ms. Janet Joyce for help with the search strategy for this study.

Funding: Research grant from Canadian Institutes of Health Research (NKS 158643), Manitoba Medical Services Foundation (# 8-2018-06) and Winnipeg Foundation (# 8-2018-06).

Competing interests: None declared.

Data sharing statement: All data relevant to the study are included in the article or uploaded as supplementary information.

Ethics statement: Not applicable.

Author contributions: MJ, AMAS, TB and MH substantially contributed to the design and conception of the study. MJ drafted the manuscript with input from all co-authors. MJ, AMAS and RR were involved in the data analysis. NA was involved in developing search strategy. LC, RA, YAY, SK, and NAY were involved in study selection process and in data extraction. MD, SB, JM, PT, BHR, AC, WS provided content expertise in emergency medicine. RS, GH, TB, and

For peer review only - http://bmjopen.bmj.com/site/about/guidelines.xhtml

JE provided content expertise in family medicine. CS provided content expertise in patient engagement. ACT, RZ and AMAS provided content expertise in scoping review methodology. All study authors provided critical feedback and approved the final manuscript.

Abbreviations:

ATS: Australian triage scale

CINAHL: Cumulative Index to Nursing and Allied Health Literature

CTAS: Canadian Triage and Acuity Scale

CBA: Controlled before and after

ED: Emergency department

EMBASE: Excerpta Medica Database

ESI: Emergency Severity Index

GP: General practitioner

JBI: Joanna Briggs Institute

LAMA: Leaving against medical advice

LOS: Length of stay

LWBS: Leaving without being seen

NP: Nurse practitioner

PHCP: Primary healthcare provider

For peer review only - http://bmjopen.bmj.com/site/about/guidelines.xhtml

PRESS: Peer Review of Electronic Search Strategies

PRISMA: Preferred Reporting Items for Systematic reviews and Meta-Analyses

ScR: Scoping Review

For peer review only

For peer review only - http://bmjopen.bmj.com/site/about/guidelines.xhtml

References:

1. Holden RJ. Lean Thinking in emergency departments: a critical review. *Annals of emergency medicine.* 2011;57(3):265-278.
2. Thrasher C, Purc-Stephenson RJ. Integrating nurse practitioners into Canadian emergency departments: a qualitative study of barriers and recommendations. *Cjem.* 2007;9(4):275-281.
3. Canadian Association of Emergency Physicians and National Emergency Nurses Affiliation. Joint position statement on emergency department overcrowding. Canadian Journal of Emergency Medecine. 2001;3(2):82-84. http://www.cjem-online.ca/v3/n2/p82.
4. Guo B, Harstall C. Strategies to reduce emergency department overcrowding. Health Technology Assesment report #38. *Alberta Heritage Foundation for Medical Research.* 2006.
5. Fee C, Weber EJ, Maak CA, Bacchetti P. Effect of emergency department crowding on time to antibiotics in patients admitted with community-acquired pneumonia. *Annals of emergency medicine.* 2007;50(5):501-509, 509 e501.
6. Pines JM, Hollander JE. Emergency department crowding is associated with poor care for patients with severe pain. *Annals of emergency medicine.* 2008;51(1):1-5.
7. Guttmann A, Schull MJ, Vermeulen MJ, Stukel TA. Association between waiting times and short term mortality and hospital admission after departure from emergency department: population based cohort study from Ontario, Canada. *Bmj.* 2011;342:d2983.
8. Fatovich DM. Effect of ambulance diversion on patient mortality: how access block can save your life. *The Medical journal of Australia.* 2005;183(11-12):672-673.
9. Fatovich DM, Nagree Y, Sprivulis P. Access block causes emergency department overcrowding and ambulance diversion in Perth, Western Australia. *Emergency medicine journal : EMJ.* 2005;22(5):351-354.
10. Asplin BR, Magid DJ, Rhodes KV, Solberg LI, Lurie N, Camargo CA, Jr. A conceptual model of emergency department crowding. *Annals of emergency medicine.* 2003;42(2):173-180.
11. Position Statement on Emergency Medicine Definitions From the Canadian Association of Emergency Physicians. *Canadian Association of Emergency Physicians 2016.*
12. Rocovich C, Patel T. Emergency department visits: Why adults choose the emergency room over a primary care physician visit during regular office hours? *World J Emerg Med.* 2012;3(2):91-97.
13. Doetzel CM, Rankin JA, Then KL. Nurse Practitioners in the Emergency Department: Barriers and Facilitators for Role Implementation. *Advanced emergency nursing journal.* 2016;38(1):43-55.
14. Statistics Canada. Access to a regular medical doctor, 2013. Available from: http://www.statcan.gc.ca/pub/82-625-x/2014001/article/14013-eng.htm.
15. Wong WB, Edgar G, Liddy C, Vaillancourt C. Can after-hours family medicine clinics represent an alternative to emergency departments? Survey of ambulatory patients seeking after-hours care. *Canadian family physician Medecin de famille canadien.* 2009;55(11):1106-1107 e1104.
16. Brousseau DC, Bergholte J, Gorelick MH. The effect of prior interactions with a primary care provider on nonurgent pediatric emergency department use. *Archives of pediatrics & adolescent medicine.* 2004;158(1):78-82.
17. Liggins K. Inappropriate attendance at accident and emergency departments: a literature review. *Journal of advanced nursing.* 1993;18(7):1141-1145.
18. Van den Heede K, Van de Voorde C. Interventions to reduce emergency department utilisation: A review of reviews. *Health policy.* 2016;120(12):1337-1349.
19. Primary Care Interventions and Strategies to Mitigate Overcrowding of Emergency Departments: A Scoping review protocol. Open Science Framework (OSF) pre-registration - https://osf.io/z2pgc. 2019.

For peer review only - http://bmjopen.bmj.com/site/about/guidelines.xhtml

20. The Joanna Briggs Institute Reviewers' Manual. 2015. Methodology for JBI Scoping Reviews. https://joannabriggs.org/assets/docs/sumari/Reviewers-Manual_Methodology-for-JBI-Scoping-Reviews_2015_v2.pdf
21. Levac D, Colquhoun H, O'Brien KK. Scoping studies: advancing the methodology. *Implementation science : IS.* 2010;5:69.
22. Tricco A, Straus S, Moher D. Preferred Reporting Items for Systematic Reviews and Meta-Analysis: extension for Scoping Reviews (PRISMA-ScR). http://www.equator-network.org/wp-content/uploads/2009/02/Executive-summary_ScR_Dec-9.pdf.
23. McGowan J, Straus S, Moher D, et al. Reporting scoping reviews-PRISMA ScR extension. *Journal of clinical epidemiology.* 2020.
24. McGowan J, Sampson M, Salzwedel DM, Cogo E, Foerster V, Lefebvre C. PRESS Peer Review of Electronic Search Strategies: 2015 Guideline Statement. *Journal of clinical epidemiology.* 2016;75:40-46.
25. Tricco AC, Lillie E, Zarin W, et al. PRISMA Extension for Scoping Reviews (PRISMA-ScR): Checklist and Explanation. *Ann Intern Med.* 2018;169(7):467-473.
26. Peters MDJ, McInerney P, Baldini Soares C, Khalil H, Parker D. Chapter 11: scoping reviews. In: Aromataris E, Munn Z, eds. *Joanna Briggs Institute Reviewer's Manual: The Joanna Briggs Institute.* 2017.
27. Staniszewska S, Brett J, Simera I, et al. GRIPP2 reporting checklists: tools to improve reporting of patient and public involvement in research. *Bmj.* 2017;358:j3453.
28. Moher D, Liberati A, Tetzlaff J, Altman DG, Group P. Preferred reporting items for systematic reviews and meta-analyses: the PRISMA statement. *PLoS medicine.* 2009;6(7):e1000097.
29. Rothwell S, McIltrot K, Khouri-Stevens Z. Addressing Emergency Department Issues Using Advanced Practice in Saudi Arabia. *Journal for Nurse Practitioners.* 2018;14(2):e41-e44.
30. Salisbury C, Hollinghurst S, Montgomery A, et al. The impact of co-located NHS walk-in centres on emergency departments. *Emergency Medicine Journal.* 2007;24(4):265-269.
31. Lutze M, Ratchford A, Fry M. A review of the Transitional Emergency Nurse Practitioner. *Australasian Emergency Nursing Journal.* 2011;14(4):226-231.
32. Ducharme J, Alder RJ, Pelletier C, Murray D, Tepper J. The impact on patient flow after the integration of nurse practitioners and physician assistants in 6 Ontario emergency departments. *CJEM Canadian Journal of Emergency Medical Care.* 2009;11(5):455-461.
33. Anantharaman V. Impact of health care system interventions on emergency department utilization and overcrowding in Singapore. *International Journal of Emergency Medicine.* 2008;1(1):11-20.
34. Lindley-Jones M, Finlayson BJ. Triage nurse requested x rays--are they worthwhile? *J Accid Emerg Med.* 2000;17(2):103-107.
35. Platter MEM, Kurvers RAJ, Janssen L, Verweij MMJ, Barten DG. The impact of an emergency care access point on pediatric attendances at the emergency department: An observational study. *The American journal of emergency medicine.* 2019.
36. Considine J, Kropman M, Kelly E, Winter C. Effect of emergency department fast track on emergency department length of stay: a case-control study. *Emergency Medicine Journal.* 2008;25(12):815-819.
37. Sterner SE, Coco T, Monroe KW, King WD, Losek JD. A new after-hours clinic model provides cost-saving, faster care compared with a pediatric emergency department. *Pediatric Emergency Care.* 2012;28(11):1162-1165.
38. Mackinney T, Visotcky AM, Tarima S, Whittle J. Does providing care for uninsured patients decrease emergency room visits and hospitalizations? *Journal of Primary Care & Community Health.* 2013;4(2):135-142.

For peer review only - http://bmjopen.bmj.com/site/about/guidelines.xhtml

39. Sharma A, Inder B. Impact of co-located general practitioner (GP) clinics and patient choice on duration of wait in the emergency department. *Emergency Medicine Journal.* 2011;28(8):658-661.
40. Nichols DE, Haber SG, Romaire MA, Wensky SG, Multi-Payer Advanced Primary Care Practice Evaluation Team. Changes in Utilization and Expenditures for Medicare Beneficiaries in Patient-centered Medical Homes: Findings From the Multi-Payer Advanced Primary Care Practice Demonstration. *Medical care.* 2018;56(9):775-783.
41. Van Uden CJT, Winkens RAG, Wesseling GJ, Crebolder HFJM, Van Schayck CP. Use of out of hours services: A comparison between two organisations. *Emergency Medicine Journal.* 2003;20(2):184-187.
42. van Gils-van Rooij ESJ, Meijboom BR, Broekman SM, Yzermans CJ, de Bakker DH. Is patient flow more efficient in Urgent Care Collaborations? *European Journal of Emergency Medicine.* 2018;25(1):58-64.
43. Arain M, Nicholl J, Campbell M. GP-led walk-in centre in the UK: Another way for urgent healthcare provision. *Critical Care.* 2013;2):S97.
44. To T, Guan J, Zhu J, et al. Quality of asthma care under different primary care models in Canada: a population-based study. *BMC family practice.* 2015;16:19.
45. Chmiel C, Wang M, Sidler P, Eichler K, Rosemann T, Senn O. Implementation of a hospital-integrated general practice--a successful way to reduce the burden of inappropriate emergency-department use. *Swiss Medical Weekly.* 2016;146:w14284.
46. Kool RB, Homberg DJ, Kamphuis HC. Towards integration of general practitioner posts and accident and emergency departments: a case study of two integrated emergency posts in the Netherlands. *BMC health services research.* 2008;8:225.
47. Wright B, Potter AJ, Trivedi AN, Mueller KJ. The Relationship Between Rural Health Clinic Use and Potentially Preventable Hospitalizations and Emergency Department Visits Among Medicare Beneficiaries. *The Journal of rural health : official journal of the American Rural Health Association and the National Rural Health Care Association.* 2018;34(4):423-430.
48. Kobewka DM, Kunkel E, Hsu A, Talarico R, Tanuseputro P. Physician Availability in Long-term Care and Resident Hospital Transfer: A Retrospective Cohort Study. *Journal of the American Medical Directors Association.* 2019.
49. Carson D, Clay H, Stern R. Primary Care and Emergency Departments. *Primary Care Foundation* 2010.
50. Rowe BH, Villa-Roel C, Guo X, et al. The role of triage nurse ordering on mitigating overcrowding in emergency departments: a systematic review. *Academic emergency medicine : official journal of the Society for Academic Emergency Medicine.* 2011;18(12):1349-1357.
51. Rowe BH, Guo X, Villa-Roel C, et al. The role of triage liaison physicians on mitigating overcrowding in emergency departments: a systematic review. *Academic emergency medicine : official journal of the Society for Academic Emergency Medicine.* 2011;18(2):111-120.
52. Fowler LH, Landry J, Nunn MF. Nurse Practitioners Improving Emergency Department Quality and Patient Outcomes. *Crit Care Nurs Clin North Am.* 2019;31(2):237-247.
53. Chan SS, Cheung NK, Graham CA, Rainer TH. Strategies and solutions to alleviate access block and overcrowding in emergency departments. *Hong Kong Med J.* 2015;21(4):345-352.
54. Goncalves-Bradley D, Khangura JK, Flodgren G, Perera R, Rowe BH, Shepperd S. Primary care professionals providing non-urgent care in hospital emergency departments. *The Cochrane database of systematic reviews.* 2018;2:CD002097.
55. Jennings N, Clifford S, Fox AR, O'Connell J, Gardner G. The impact of nurse practitioner services on cost, quality of care, satisfaction and waiting times in the emergency department: a systematic review. *International journal of nursing studies.* 2015;52(1):421-435.

For peer review only - http://bmjopen.bmj.com/site/about/guidelines.xhtml

56. Ansell D, Crispo JAG, Simard B, Bjerre LM. Interventions to reduce wait times for primary care appointments: a systematic review. *BMC health services research.* 2017;17(1):295.

For peer review only

For peer review only - http://bmjopen.bmj.com/site/about/guidelines.xhtml

Table 1: Characteristics of included studies in a scoping review of primary healthcare professional interventions to address emergency department overcrowding.

**Characteristics**		**n (%) (N = 268)**
**Publication type**	Journal article	206 (76.9)
	Abstract	62 (23.1)
**Continents**	North America	156 (58.2)
	Europe	76 (28.4)
	Oceania	22 (8.2)
	Asia	9 (3.4)
	Middle east	3 (1.0)
	South America	1 (0.4)
	Not reported	1 (0.4)
**Type of ED (rural versus urban)**	Rural ED	5 (2.0)
	Urban ED	137 (51.0)
	Both	16 (6.0)
	Not reported	110 (41.0)
**Type of ED (adult versus other)**	Adult ED	19 (7.0)
	Pediatric ED	24 (9.0)
	Mixed ED	79 (29.5)
	Not reported	146 (54.5)
**Type of intervention or strategy**	Within ED	129 (48.0)
	Outside ED	139 (52.0)
**Type of primary healthcare professional**	Family Physician (FP)	85 (31.7)
	Nurse practitioner (NP)	69 (25.7)
	Nurse with expanded role	43 (16.0)
	FP & NP	15 (5.6)
	FP & Nurse	2 (0.8)
	NP & Nurse	8 (3.0)
	Not reported	46 (17.2)
**Nature of intervention or strategy**	Added a primary healthcare professional	190 (71.0)
	Increased responsibility	59 (22.0)
	Not reported	19 (7.0)
**Study designs**	Randomized clinical trial	22 (8.2)
	Non-randomized trial	9 (3.4)
	Cohort study (prospective or retrospective)	112 (41.8)
	Case-control study	4 (1.5)
	Cross-sectional study	27 (10.0)
	Pre-post study	76 (28.4)
	Interrupted time series	14 (5.2)
	Mixed-methods study	4 (1.5)

For peer review only - http://bmjopen.bmj.com/site/about/guidelines.xhtml

Figure legends

Figure 1: PRISMA study flow chart for scoping review of primary healthcare professional interventions to address emergency department overcrowding.

Figure 2: Number of publications per year involving primary healthcare professional interventions to address emergency department overcrowding.

For peer review only

For peer review only - http://bmjopen.bmj.com/site/about/guidelines.xhtml

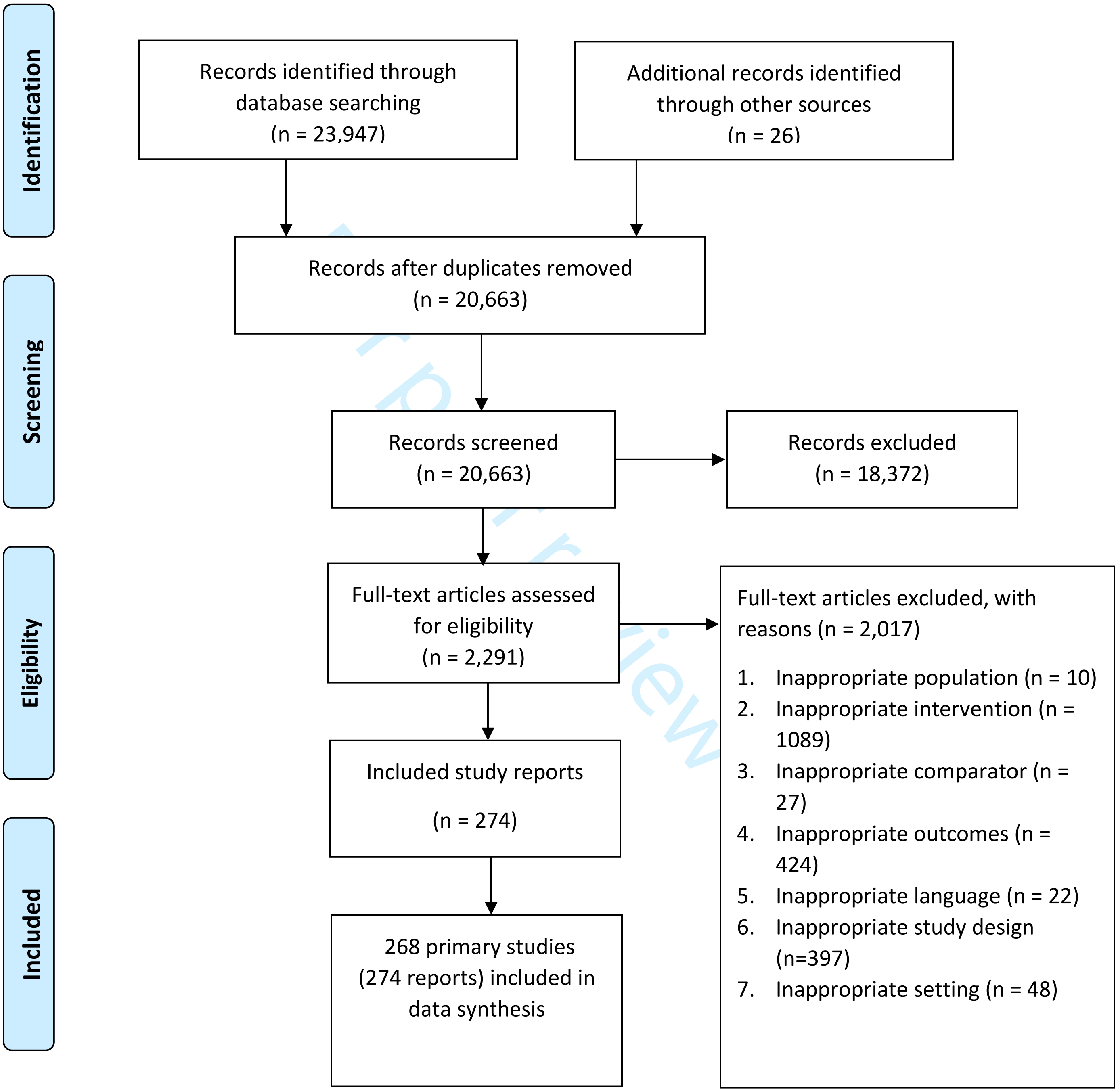

For peer review only - http://bmjopen.bmj.com/site/about/guidelines.xhtml

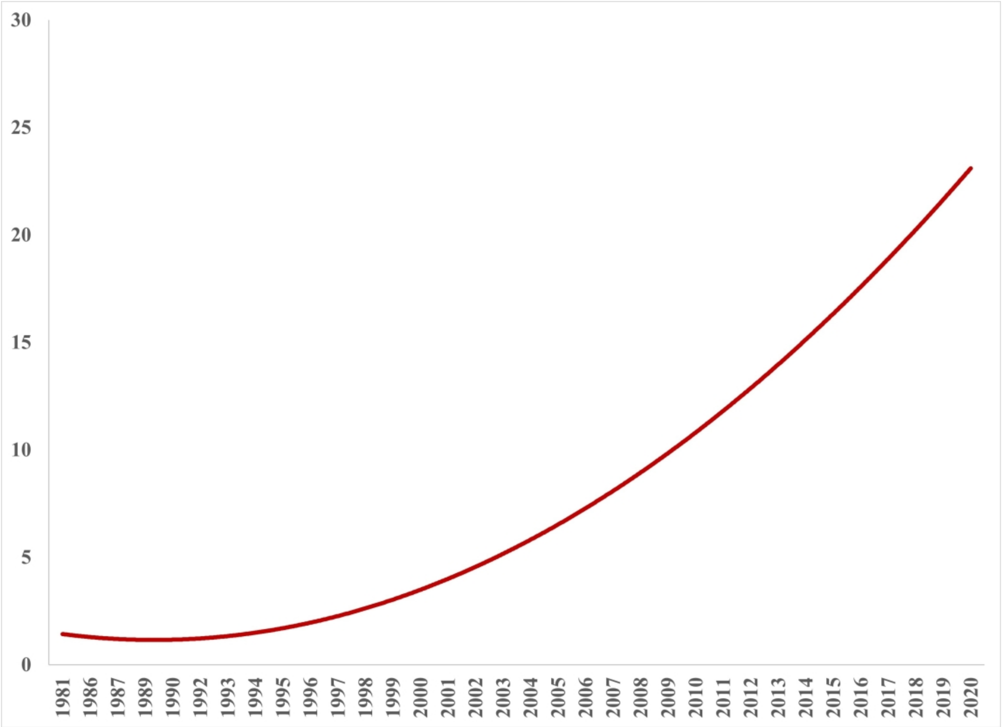

Number of publications per year involving primary healthcare professional interventions to address emergency department overcrowding.

240x174mm (330 x 330 DPI)

Appendix Table 1: Population, Concept and Context

**Population, Concept and Context**	
**Population**	Any patient population (children and adults of any age) visiting ED
**Intervention**	Any intervention or strategy involving primary healthcare professionals (family physician/general practitioner (GP), nurse practitioner (NP) & nurse with expanded authority) that was implemented to reduce overcrowding of ED
**Outcomes**	All ED outcomes that reflect the impact of interventions and strategies involving primary healthcare professionals (e.g., proportion of patients that leave ED without being seen (LWBS), ED length of stay (LOS), patient satisfaction, mean/median patient wait times in ED, percentage of patients with reduced wait times in ED, time to initial physician assessment, and volume of patients visiting ED)
**Study designs**	All study designs will be included except the following publication types: narrative reviews, editorials, commentaries, and historical articles.
**Publication status**	Only data from published studies will be included for feasibility.
**Language limits**	Only English language publications will be included for feasibility.
**Years considered**	No restriction on publication dates

For peer review only

Appendix Table 2: Medline Search Strategy

Database: Ovid MEDLINE(R) and Epub Ahead of Print, In-Process & Other Non-Indexed Citations and Daily <1946 to January 10, 2020>
Search Strategy:
----
1 exp primary health care/ (140156)
2 physicians, family/ (15853)
3 family practice/ (64029)
4 Physicians, Primary Care/ (2703)
5 general practice/ (11719)
6 general practitioners/ (6381)
7 (primary adj2 (care or health*)).ti,ab,kf. (129329)
8 ((general or family) adj (practice* or practitioner*)).ti,ab,kf. (84810)
9 (GP or GPs).ti,ab,kf. (51935)
10 nurse practitioners/ (16770)
11 primary care nursing/ (392)
12 family nursing/ (1349)
13 community mental health services/ (17905)
14 ((family or community or primary or ambulatory or triage) adj2 (medic* or doctor* or physician* or health* or nurs*)).ti,ab,kf. (68438)
15 Ambulatory Care/ (40524)
16 (ambulatory adj2 care).ti,ab,kf. (11413)
17 Health Services, Indigenous/ (2817)
18 Cultural Competency/ (4632)
19 Culturally Competent Care/ (830)
20 Medicine, Traditional/ (10299)
21 (trauma adj inform*).ti,ab,kf. (617)
22 (aborigin* or indigenous or native).ti,ab,kf. (225451)
23 ((after or out) adj2 hour*).ti,ab,kf. (137459)
24 or/1-23 (917850)
25 exp Emergency Service, Hospital/ (67418)
26 Emergency Medical Services/ (39232)
27 emergency treatment/ (10025)
28 Trauma centers/ (9210)
29 Triage/ (10240)
30 ((emergency or emergent or urgent) adj2 (care or healthcare or department* or unit or units or room* or treatment* or ward or service)).ti,ab,kf. (121497)
31 ("accident and emergency" or "accident & emergency" or ED or EDs or ER or A&E).ti,ab,kf. (162648)
32 (triage adj2 (centre or centres or center or centers or department? or unit or units)).ti,ab,kf. (538)
33 (emergency adj2 (care or healthcare or department? or unit or units or room? or treatment? or care or visit? or utilization or admit or admission?)).ti,ab,kf. (112731)
34 ("accident and emergency" or "accident & emergency" or emergency service?).ti,ab,kf. (10865)

35 (trauma adj2 (centre or centres or center or centers or department? or unit or units)).ti,ab,kf. (15573)
36 (triage adj2 (centre or centres or center or centers or department? or unit or units)).ti,ab,kf. (538)
37 (emergency adj2 (visit? or care or admit or admission?)).ti,ab,kf. (26760)
38 (urgent adj2 (care or healthcare or health care)).ti,ab,kf. (2099)
39 ((semiurgent or semi-urgent or nonemergen$ or non-emergen$) adj2 (treatment? or care or visit?)).ti,ab,kf. (289)
40 ((emergency or non-emergency or nonemergency or urgent or non-urgent or nonurgent or semi-urgent or semiurgent) adj2 patient?).ti,ab,kf. (11636)
41 or/25-40 (367776)
42 organizational efficiency/ (20744)
43 workflow/ (3295)
44 Waiting lists/ (10724)
45 ((wait or waiting) adj2 (time or times or list or lists)).ti. (3351)
46 ((wait or waiting or throughput or service or treatment) adj2 (time or times or list or lists) adj10 (reduce? or reduction or eliminat$ or lower or fewer or intervention or policy or policies or reform$ or effectiveness or impact or improv$ or organi?ational$ or quality or save or saving)).ab. (3119)
47 ((decrease or reduce or streamline or less or minimize or shorten or eliminate or cut or enhance or facilitate or speed or better or accelerate or optimize or reform or delay or change or faster or impact$ or assess$ or eliminat$ or improv$ or lower$ or reduc$) adj3 patient? wait$).ti,ab,kf. (303)
48 CROWDING/ (2930)
49 crowd$.ti,ab,kf. (16513)
50 congest$.ti,ab,kf. (61747)
51 overcrowd$.ti,ab,kf. (3425)
52 gridlock$.ti,ab,kf. (180)
53 queue$.ti,ab,kf. (1011)
54 overload$.ti,ab. (39413)
55 "access block$".ti,ab,kf. (166)
56 (throughput or through-put).ti,ab,kf. (87262)
57 warehous$.ti,ab,kf. (2303)
58 ("left without being seen" or "leave$ without being seen" or lwbs).ti,ab,kf. (284)
59 (patient adj2 elop$).ti,ab,kf. (16)
60 (ambulance$ adj2 diver$).ti,ab,kf. (194)
61 (ambulance$ adj2 redirect$).ti,ab,kf. (3)
62 "fast track$".ti,ab,kf. (3500)
63 delay$.ti,ab,kf. (428757)
64 ("patient flow$" or "flow of patient$").ti,ab,kf. (4939)
65 defer$.ti,ab,kf. (23198)
66 (over* adj3 (capacit$ or occupanc$)).ti,ab,kf. (4603)
67 (lama or (leave$ adj4 ("medical advice" or treatment$)) or (left adj4 ("medical advice" or treatment$))).ti,ab,kf. (8393)
68 ((hallway or corridor) adj2 (care or medicine)).ti,ab,kf. (6)
69 or/42-68 (776721)
70 24 and 41 and 69 (3799)

Appendix Table 3: Grey literature sources

Grey literature sources
BMJ Open Quality (https://bmjopenquality.bmj.com) and a Google Custom Search of the following websites:<br>Canadian Foundation for Healthcare Improvement (www.cfhi-fcass.ca), Institute for Healthcare Improvement (www.ihi.org), Agency for Healthcare Research and Quality (www.ahrq.gov), NHS Improvement (https://improvement.nhs.uk), International Society for Quality in Health Care (www.isqua.org), Health Quality Ontario (www.hqontario.ca), Saskatchewan Health Quality Council (https://hqc.sk.ca), Health Quality Council of Alberta (www.hqca.ca), BC Patient Safety & Quality Council (https://bcpsqc.ca), Australian Commission on Safety and Quality in Health Care (www.safetyandquality.gov.au), and Health Quality & Safety Commission New Zealand (www.hqsc.govt.nz).

For peer review only

Appendix 4: List of studies included in the scoping review

1. Adam H, Tamim H, Altamimi S, et al. Effect of triage nurse ordered distal extremity X-rays on emergency department length of stay: A randomized controlled trial. *Academic Emergency Medicine.* 2014;21(5 SUPPL. 1):S195.
2. Al Abri FH, Muliira JK, Al Awaisi H. Effect of triage nurse-led application of the Ottawa Ankle Rules on number of radiographic tests and length of stay in selected emergency departments in Oman. *Japan journal of nursing science : JJNS.* 2020;17(1):e12270.
3. Al Kadhi O, Manley K, Natarajan M, et al. A renal colic fast track pathway to improve waiting times and outcomes for patients presenting to the emergency department. *Open access emergency medicine : OAEM.* 2017;9:53-55.
4. Alcusky M, Singer D, Keith SW, et al. Evaluation of Care Processes and Health Care Utilization in Newly Implemented Medical Homes in Italy: A Population-Based Cross-sectional Study. *Am J Med Qual.* 2019:1062860619860590.
5. Almeida A, Vales J. The impact of primary health care reform on hospital emergency department overcrowding: Evidence from the Portuguese reform. *The International journal of health planning and management.* 2019.
6. Anantharaman V. Impact of health care system interventions on emergency department utilization and overcrowding in Singapore. *International Journal of Emergency Medicine.* 2008;1(1):11-20.
7. Ankeny A, Isenberger K, Westgard B, Stuck L, Wewerka S. Association of missed outpatient appointments and emergency department visits and inpatient admissions. *Annals of Emergency Medicine.* 2014;1):S87-S88.
8. Anonymous. Mobile unit helps ED cut LWBS in half. *ED management : the monthly update on emergency department management.* 2008;20(3):31-32.
9. Arain M, Nicholl J, Campbell M. GP-led walk-in centre in the UK: Another way for urgent healthcare provision. *Critical Care.* 2013;2):S97.
10. Ashurst JV, Nappe T, Digiambattista S, et al. Effect of triage-based use of the Ottawa foot and ankle rules on the number of orders for radiographic imaging. *The Journal of the American Osteopathic Association.* 2014;114(12):890-897.
11. Badgett JT. Can Medicaid format alter emergency department utilization patterns? *Pediatric Emergency Care.* 1986;2(2):67-70.
12. Barr M, Johnston D, McConnell D. Patient satisfaction with a new nurse practitioner service. *Accident and emergency nursing.* 2000;8(3):144-147.
13. Baughman AW, Li Z, Friedberg MW, et al. Impact of the patient-centered medical home on patients with depression. *Journal of General Internal Medicine.* 2016;1):S265-S266.
14. Beales J. Innovation in accident and emergency management: establishing a nurse practitioner-run minor injuries/primary care unit. *Accident and emergency nursing.* 1997;5(2):71-75.
15. Beales J, Baker B. Minor Injuries Unit: expanding the scope of accident and emergency provision. *Accident and emergency nursing.* 1995;3(2):65-67.
16. Begaz T, Salem R, Agarwal N, Duan L, Taira B. Initiating workups in the waiting room decreases emergency department bed time and left before completion of service rate. *Annals of Emergency Medicine.* 2015;1):S74.
17. Begum F, Khan H, Moss P. Solving the A & E crisis using GP lead triage and redirection Evaluation of GP-lead service to identify and re-direct patients from A & E to primary care services. *https://wwwhealthylondonorg/wp-content/uploads/2017/10/30-Solving-the-AE-crisis-using-GP-lead-triage-and-redirectionpdf.* 2016.

For peer review only - http://bmjopen.bmj.com/site/about/guidelines.xhtml

18. Bitton A. Medical home pilot lowers costs, improves quality and patient/staff experience. *Journal of Clinical Outcomes Management.* 2009;16(12):541-544.
19. Bleijenberg N, Drubbel I, Schuurmans MJ, et al. Effectiveness of a Proactive Primary Care Program on Preserving Daily Functioning of Older People: A Cluster Randomized Controlled Trial. *Journal of the American Geriatrics Society.* 2016;64(9):1779-1788.
20. Bodenmann P, Velonaki VS, Baggio S, et al. Case management for emergency department frequent users: a randomized controlled trial. *Praxis.* 2015;104:17-18.
21. Boeke AJP, van Randwijck-Jacobze ME, de Lange-Klerk EM, Grol SM, Kramer MH, van der Horst HE. Effectiveness of GPs in accident and emergency departments. *The British journal of general practice : the journal of the Royal College of General Practitioners.* 2010;60(579):e378-384.
22. Bonham GS, Barber GM. Use of health care before and during Citicare. *Medical Care.* 1987;25(2):111-119.
23. Bottle A, Honeyford K, Chowdhury F, Bell D, Aylin P. 2018.
24. Breslau J, Leckman-Westin E, Han B, et al. Impact of a mental health based primary care program on emergency department visits and inpatient stays. *General Hospital Psychiatry.* 2018;52:8-13.
25. Buerhaus P, Perloff J, Clarke S, O'Reilly-Jacob M, Zolotusky G, DesRoches CM. Quality of Primary Care Provided to Medicare Beneficiaries by Nurse Practitioners and Physicians. *Medical Care.* 2018;56(6):484-490.
26. Bynum JPW, Andrews A, Sharp S, McCullough D, Wennberg JE. Fewer hospitalizations result when primary care is highly integrated into a continuing care retirement community. *Health Affairs.* 2011;30(5):975-984.
27. Byrne G, Richardson M, Brunsdon J, Patel A. An evaluation of the care of patients with minor injuries in emergency settings. *Accident and emergency nursing.* 2000;8(2):101-109.
28. Campbell S, Atkinson P, Fraser J, Stewart C, Middleton J, McCloskey R. Nurse practitioners in the emergency department: A solution to long wait times and high walkout rates? *Canadian Journal of Emergency Medicine.* 2012;1):S11.
29. Carter A. The Ambulatory Care Unit at Derriford Hospital. *Clinical Medicine.* 2014;14(3):250-254.
30. Carter R, Quesnel-Vallee A, Plante C, Gamache P, Levesque JF. Effect of family medicine groups on visits to the emergency department among diabetic patients in Quebec between 2000 and 2011: a population-based segmented regression analysis. *BMC family practice.* 2016;17:23.
31. Casalino E, Choquet C, Curac S, et al. An evaluation of hospital attractiveness and primary care availability leading to increasing emergency department visits. *Public Health.* 2017;151:27-30.
32. Cecil E, Bottle A, Cowling TE, Majeed A, Wolfe I, Saxena S. Primary care access, emergency department visits, and unplanned short hospitalizations in the UK. *Pediatrics.* 2016;137 (2) (no pagination)(e20151492).
33. Celona CA, Amaranto A, Ferrer R, et al. Interdisciplinary Design to Improve Fast Track in the Emergency Department. *Advanced Emergency Nursing Journal.* 2018;40(3):198-203.
34. Chakravorty S, Knapp CA. The Impact of the Patient-Centered Medical Home on Asthma-Related Visits to the Emergency Room: A Fixed Effects Regression Approach. *Maternal and child health journal.* 2019;23(3):369-376.
35. Chang T, Kuo R. The Association of Quality of Primary Care and the Potentially Avoidable Emergency Department (Ed) Visit. *Value in Health.* 2018;21(Supplement 2):S49.
36. Cheung WWH, Heeney L, Pound JL. An advance triage system. *Accident & Emergency Nursing.* 2002;10(1):10-16.

For peer review only

37. Chmiel C, Wang M, Sidler P, Eichler K, Rosemann T, Senn O. Implementation of a hospital-integrated general practice--a successful way to reduce the burden of inappropriate emergency-department use. *Swiss Medical Weekly.* 2016;146:w14284.
38. Chu L, Sood N, Tu M, Miller K, Ray L, Sayles JN. Reduction of Emergency Department Use in People With Disabilities. *American Journal of Managed Care.* 2017;23(12):e409-e415.
39. Chu LH, Tu M, Lee YC, Sood N. The impact of patient-centered medical homes on safety net clinics. *American Journal of Managed Care.* 2016;22(87):532-538.
40. Clancy E, Mayo A. Launching a social enterprise see-and-treat service. *Emergency nurse : the journal of the RCN Accident and Emergency Nursing Association.* 2009;17(3):22-24.
41. Cloutier MM, Hall CB, Wakefield DB, Bailit H. Use of asthma guidelines by primary care providers to reduce hospitalizations and emergency department visits in poor, minority, urban children. *Journal of Pediatrics.* 2005;146(5):591-597.
42. Colligan M, Collins C, Foley B, Jones P, Miles J, Zeng I. Emergency nurse practitioners: do they provide an effective service in managing minor injuries, compared to emergency medicine registrars? *New Zealand Medical Journal.* 2011;124(1344):74-80.
43. Considine J, Kropman M, Kelly E, Winter C. Effect of emergency department fast track on emergency department length of stay: a case-control study. *Emergency Medicine Journal.* 2008;25(12):815-819.
44. Considine J, Martin R, Smit D, Winter C, Jenkins J. Emergency nurse practitioner care and emergency department patient flow: case-control study. *Emergency Medicine Australasia.* 2006;18(4):385-390.
45. Conway A, Richardson A. The respiratory nurse specialist role at a medical assessment unit. *Nursing times.* 2004;100(24):53-54.
46. Cooper MA, Lindsay GM, Kinn S, Swann IJ. Evaluating Emergency Nurse Practitioner services: a randomized controlled trial. *Journal of advanced nursing.* 2002;40(6):721-730.
47. Copeland J, Gray A. A Daytime Fast Track Improves Throughput in a Single Physician Coverage Emergency Department. *CJEM.* 2015;17(6):648-655.
48. Covington C, Erwin T, Sellers F. Implementation of a nurse practitioner-staffed fast track. *Journal of emergency nursing: JEN : official publication of the Emergency Department Nurses Association.* 1992;18(2):124-131.
49. Crits-Christoph P, Gallop R, Noll E, et al. Impact of a medical home model on costs and utilization among comorbid HIV-positive medicaid patients. *American Journal of Managed Care.* 2018;24(8):368-375.
50. Cuellar A, Helmchen LA, Gimm G, et al. The CareFirst Patient-Centered Medical Home Program: Cost and Utilization Effects in Its First Three Years. *Journal of General Internal Medicine.* 2016;31(11):1382-1388.
51. Dahrouge S, Hogg W, Younger J, Muggah E, Russell G, Glazier RH. Primary Care Physician Panel Size and Quality of Care: A Population-Based Study in Ontario, Canada. *Annals of Family Medicine.* 2016;14(1):26-33.
52. Dale J, Lang H, Roberts JA, Green J, Glucksman E. Cost effectiveness of treating primary care patients in accident and emergency: a comparison between general practitioners, senior house officers, and registrars. *BMJ.* 1996;312(7042):1340-1344.
53. Day TE, Al-Roubaie AR, Goldlust EJ. Decreased length of stay after addition of healthcare provider in emergency department triage: a comparison between computer-simulated and real-world interventions. *Emerg Med J.* 2013;30(2):134-138.
54. Demarco F, Gerardo CJ, Boardwine A, et al. Effect of a nurse rapid intake initiative on patient length of stay and satisfaction: A project IMPACT initiative. *Academic Emergency Medicine.* 2010;17(SUPPL. 1):S93-S94.

For peer review only

55. Derlet RW, Nishio D, Cole LM, Silva J, Jr. Triage of patients out of the emergency department: three-year experience. *The American journal of emergency medicine.* 1992;10(3):195-199.
56. Dewhirst S, Vaillancourt C, Mackenzie T, Zhao Y. Evaluating a nurse initiated analgesia protocol in the emergency department. *Canadian Journal of Emergency Medicine.* 2015;17(Supplement 2):S60-S61.
57. Dias RD, Rios IC, Canhada CL, et al. Using the Manchester triage system for refusing nonurgent patients in the emergency department: A 30-day outcome study. *Journal of Emergency Management.* 2016;14(5):349-364.
58. Dinh M, Walker A, Parameswaran A, Enright N. Evaluating the quality of care delivered by an emergency department fast track unit with both nurse practitioners and doctors. *Australasian Emergency Nursing Journal.* 2012;15(4):188-194.
59. Dixon A, Clarkin C, Barrowman N, Correll R, Osmond MH, Plint AC. Reduction of radial-head subluxation in children by triage nurses in the emergency department: A cluster-randomized controlled trial. *CMAJ.* 2014;186(9):E317-E323.
60. Dolton P, Pathania V. Can increased primary care access reduce demand for emergency care? Evidence from England's 7-day GP opening. *Journal of Health Economics.* 2016;49:193-208.
61. Dorado S, Pascual S, Urrutia I, et al. Prevalence of asthma and management of exacerbations in general practice (GP) after computerised Spanish guideline on the management of asthma (GEMA) and collaborative care environment. *European Respiratory Journal Conference: European Respiratory Society Annual Congress.* 2015;46(SUPPL. 59).
62. Douma MJ, Drake CA, O'Dochartaigh D, Smith KE, O'Dochartaigh D. A Pragmatic Randomized Evaluation of a Nurse-Initiated Protocol to Improve Timeliness of Care in an Urban Emergency Department. *Annals of Emergency Medicine.* 2016;68(5):546-552.
63. Driscoll DL, Hiratsuka V, Johnston JM, et al. Process and outcomes of patient-centered medical care with Alaska Native people at Southcentral Foundation. *Annals of family medicine.* 2013;11(Supplement 1):S41-S49.
64. Ducharme J, Alder RJ, Pelletier C, Murray D, Tepper J. The impact on patient flow after the integration of nurse practitioners and physician assistants in 6 Ontario emergency departments. *CJEM Canadian Journal of Emergency Medical Care.* 2009;11(5):455-461.
65. Edwards T. How rapid assessment at triage can improve care outcomes. *Emergency Nurse.* 2011;19(6):27-30.
66. Eichler K, Hess S, Chmiel C, et al. Sustained health-economic effects after reorganisation of a Swiss hospital emergency centre: a cost comparison study. *Emergency Medicine Journal.* 2014;31(10):818-823.
67. Eichler K, Senn O, Ruthemann I, Bogli K, Sidler P, Brugger U. Reorganisation of hospital emergency services: A business case for quality improvement. *Value in Health.* 2011;14 (7):A344-A345.
68. Fan J, Woolfrey K. The effect of triage-applied Ottawa Ankle Rules on the length of stay in a Canadian urgent care department: a randomized controlled trial. *Academic emergency medicine : official journal of the Society for Academic Emergency Medicine.* 2006;13(2):153-157.
69. Fanta K, Cook B, Falcone RA, et al. Pediatric trauma nurse practitioners provide excellent care with superior patient satisfaction for injured children. *Journal of pediatric surgery.* 2006;41(1):277-281.
70. Farion KJ, Tse S, Patrice J, et al. IMPACT of a tertiary pediatric emergency department ambulatory zone on wait times of both high and low acuity patients. *Canadian Journal of Emergency Medicine.* 2010;12 (3):239.

For peer review only

71. Fleetcroft R, Martin A, Coombes E, Ford J, Steel N, Noble M. Emergency hospital admissions for asthma and access to primary care: Cross-sectional analysis. *British Journal of General Practice.* 2016;66(650):e640-e666.
72. Fontanel A, Besson C, Gallegos C, Vallot C, Droal D. Can X-rays be prescribed by a nurse in an emergency department? *European Journal of Emergency Medicine.* 2011;18(5):310-311.
73. Fry M, Fong J. A 12-month prospective review of the impact of Emergency Transitional Nurse Practitioners in one metropolitan emergency department...2009 CENA International Conference for Emergency Nursing. *Australasian Emergency Nursing Journal.* 2009;12(4):164-165.
74. Fry M, Fong J, Asha S, Arendts G. A 12-month evaluation of the impact of Transitional Emergency Nurse Practitioners in one metropolitan Emergency Department. *Australasian Emergency Nursing Journal.* 2011;14(1):4-8.
75. Gabayan GZ, Asch SM, Starks SL, Sun BC. Does having and using a usual source of care decrease emergency department use? *Annals of Emergency Medicine.* 2009;1):S64.
76. Gardner RM, Friedman N, Bradham T, Barrett TW. Impact of revised triage approach to improving emergency department throughput for treat and release patients. *Academic Emergency Medicine.* 2017;24 (Supplement 1):S157.
77. Gardner RM, Friedman NA, Carlson M, Bradham TS, Barrett TW. Impact of revised triage to improve throughput in an ED with limited traditional fast track population. *American Journal of Emergency Medicine.* 2018;36(1):124-127.
78. Gaucher N, Bailey B, Gravel J. Triage nurses' counselling influences return visits of children leaving the emergency department before being seen by a physician. *Academic Emergency Medicine.* 2010;17(SUPPL. 1):S118-S119.
79. Gedmintas A, Atkinson P, Fraser J, Howlett M. Impact of a new emergency department and process changes on quality indicators. *Canadian Journal of Emergency Medicine.* 2012;1):S11.
80. Grasso MA, Cotter B, Jerrard DA. The impact of a follow-up clinic on unscheduled return visits to an emergency department. *Annals of Emergency Medicine.* 2015;1):S19.
81. Gray C. Advanced practice in emergency care: the paediatric flow nurse. *Nursing Children & Young People.* 2016;28(4):33-38.
82. Green LA, Chang HC, Markovitz AR, Paustian ML. The Reduction in ED and Hospital Admissions in Medical Home Practices Is Specific to Primary Care-Sensitive Chronic Conditions. *Health Services Research.* 2018;53(2):1163-1179.
83. Hackman JL, Roth ED, Gaddis ML, Gratton MC. The effect of a nurse-initiated chest pain protocol on disposition time: A retrospective review. *Annals of Emergency Medicine.* 2015;66(4 SUPPL. 1):S9-S10.
84. Hansagi H. Referral of non-urgent cases from an emergency department: patient compliance, satisfaction and attitudes. *Scandinavian Journal of Social Medicine.* 1990;18(4):249-255.
85. Hansagi H, Allebeck P, Edhag O. Health care utilization after referral from a hospital emergency department. *Scandinavian Journal of Social Medicine.* 1989;17(4):291-299.
86. Harris MD, Kirsh S, Higgins PA. Shared Medical Appointments: Impact on Clinical and Quality Outcomes in Veterans With Diabetes. *Quality management in health care.* 2016;25(3):176-180.
87. Hayden C, Burlingame P, Thompson H, Sabol VK. Improving patient flow in the emergency department by placing a family nurse practitioner in triage: a quality-improvement project. *Journal of Emergency Nursing.* 2014;40(4):346-351.
88. Heaney D, Paxton F. Evaluation of a nurse-led minor injuries unit. *Nursing standard (Royal College of Nursing (Great Britain) : 1987).* 1997;12(4):35-38.

For peer review only

89. Hearld KR, Hearld LR, Landry AY, Budhwani H. Evidence that patient-centered medical homes are effective in reducing emergency department admissions for patients with depression. *Health Services Management Research.* 2018:951484818794340.
90. Hearld LR, Alexander JA. Patient-centered care and emergency department utilization: a path analysis of the mediating effects of care coordination and delays in care. *Medical Care Research & Review.* 2012;69(5):560-580.
91. Hearld LR, Hearld KR, Guerrazzi C. Patient-Centered Medical Home Capacity and Ambulatory Care Utilization. *American Journal of Medical Quality.* 2017;32(5):508-517.
92. Hebert P, Liu CF, Wong E, et al. National evaluation of the effects on healthcare utilization and costs of the va patient centered medical home initiative. *Journal of General Internal Medicine.* 2013;1):S124.
93. Ho JK-M, Chau JP-C, Chan JT-S, Yau CH-Y. Nurse-initiated radiographic-test protocol for ankle injuries: A randomized controlled trial. *International emergency nursing.* 2018;41:1-6.
94. Hunold KM, Richmond NL, Waller AE, Cutchin MP, Voss PR, Platts-Mills TF. Primary care availability and emergency department use by older adults: A population-based analysis. *Journal of the American Geriatrics Society.* 2014;62(9):1699-1706.
95. Hwang W, Liao K, Griffin L, Foley KL. Do free clinics reduce unnecessary emergency department visits? The Virginian experience. *Journal of Health Care for the Poor and Underserved.* 2012;23(3):1189-1204.
96. Ingram SJ, Maloney A, McDonnell N, et al. Impact of a nurse led ambulatory low-intermediate risk chest pain service. *European Heart Journal: Acute Cardiovascular Care.* 2013;1):127.
97. James D, Kithany H, Robbins A, Crouch R. Children and young people seen in a colocated primary care hub; an observational study. *Archives of Disease in Childhood.* 2019;104(Supplement 2):A134.
98. Jamshed N, Cawthon C, Rubin C. Impact analysis of a home based primary care program (HBPC) next generation aco (NGACO) model. *Journal of the American Geriatrics Society.* 2018;66 (Supplement 2):S122.
99. Jeanmonod R, Delcollo J, Jeanmonod D, Dombchewsky O, Reiter M. Comparison of resident and mid-level provider productivity and patient satisfaction in an emergency department fast track. *Emergency Medicine Journal.* 2013;30(1):e12.
100. Jennings N, Gardner G, O'Reilly G, Mitra B. Emergency NP Model of Care in an Australian Emergency Department. *Journal for Nurse Practitioners.* 2015;11(8):774-781.
101. Jennings N, O'Reilly G, Lee G, Cameron P, Free B, Bailey M. Evaluating outcomes of the emergency nurse practitioner role in a major urban emergency department, Melbourne, Australia. *Journal of Clinical Nursing.* 2008;17(8):1044-1050.
102. Jobé J, Vandercleyen C, Ghuysen A, D'Orio V. Prospective study of an advanced nurse triage for a target pathology at the admission in the emergency department. *Acta clinica belgica.* 2013;68(6):482-.
103. Johnson P, Linzer M, Shippee ND, Heegaard W, Webb F, Vickery KD. Development and Implementation of an Interdisciplinary Intensive Primary Care Clinic for High-Need High-Cost Patients in a Safety Net Hospital. *Population health management.* 2019.
104. Jones D. How GPs can help reduce inappropriate attendance. *Emergency Nurse.* 2011;19(4):20-23.
105. Kao YH, Tseng TS, Ng YY, Wu SC. Association between continuity of care and emergency department visits and hospitalization in senior adults with asthma-COPD overlap. *Health Policy.* 2019;123(2):222-228.

For peer review only - http://bmjopen.bmj.com/site/about/guidelines.xhtml

106. Keogh C, O'Brien KK, Hoban A, O'Carroll A, Fahey T. Health and use of health services of people who are homeless and at risk of homelessness who receive free primary health care in Dublin. *BMC health services research.* 2015;15:58.
107. Kern LM, Seirup JK, Rajan M, Jawahar R, Stuard SS. Fragmented ambulatory care and subsequent healthcare utilization among medicare beneficiaries. *American Journal of Managed Care.* 2018;24(9):e278-e284.
108. Kern LM, Seirup JK, Rajan M, Jawahar R, Stuard SS. Fragmented ambulatory care and subsequent emergency department visits and hospital admissions among Medicaid beneficiaries. *The American journal of managed care.* 2019;25(3):107-112.
109. Kim KM, Jeon H, Lee JH. Having a Physician Rather than a Place as a Usual Source of Care Would Be Better - from 2012 Korea Health Panel Data. *Journal of Korean medical science.* 2017;32(1):4-12.
110. Kim M, Asche CV, Tillis W, Ren J. Association between availability of care providers and healthcare utilizations among adults with asthma. *Current Medical Research & Opinion.* 2017;33(3):479-487.
111. Klassen TP, Ropp LJ, Sutcliffe T, et al. A randomized, controlled trial of radiograph ordering for extremity trauma in a pediatric emergency department. *Annals of emergency medicine.* 1993;22(10):1524-1529.
112. Klemmer DK, Ziebel C, Sharif N, Grubb S, Sookram S. Clinical lead nurse practitioner Strathcona Community Hospital. *Canadian Journal of Emergency Medicine.* 2018;20 (Supplement 1):S85.
113. Kobewka DM, Kunkel E, Hsu A, Talarico R, Tanuseputro P. Physician Availability in Long-term Care and Resident Hospital Transfer: A Retrospective Cohort Study. *Journal of the American Medical Directors Association.* 2019.
114. Kool RB, Homberg DJ, Kamphuis HC. Towards integration of general practitioner posts and accident and emergency departments: a case study of two integrated emergency posts in the Netherlands. *BMC Health Services Research.* 2008;8:225.
115. Krakau I. Trends in use of health care services in Swedish primary care district. A ten year perspective. *Scandinavian Journal of Primary Health Care.* 1992;10(1):66-71.
116. Kravet SJ, Shore AD, Miller R, Green GB, Kolodner K, Wright SM. Health Care Utilization and the Proportion of Primary Care Physicians. *American Journal of Medicine.* 2008;121(2):142-148.
117. Krook CE, Parks P. Emergency department follow-up for unattached patients. *Canadian Journal of Emergency Medicine.* 2013;15 (Suppl 1):S5.
118. Krupski A, West II, Scharf DM, et al. Integrating primary care into community mental health centers: Impact on utilization and costs of health care. *Psychiatric Services.* 2016;67(11):1233-1239.
119. Kuo Y-F, Adhikari D, Eke CG, Goodwin JS, Raji MA. Processes and Outcomes of Congestive Heart Failure Care by Different Types of Primary Care Models. *Journal of cardiac failure.* 2018;24(1):9-18.
120. Lagisetty PA, Hu HM, Goesling J, et al. Care coordination and adverse opioid related outcomes: The role of the usual provider. *Journal of General Internal Medicine.* 2018;33(2 Supplement 1):134.
121. Laine C, Lin YT, Hauck WW, Turner BJ. Availability of medical care services in drug treatment clinics associated with lower repeated emergency department use. *Medical Care.* 2005;43(10):985-995.

For peer review only

122. Lee C, Sung NJ, Lim HS, Lee JH. Emergency Department Visits Can Be Reduced by Having a Regular Doctor for Adults with Diabetes Mellitus: Secondary Analysis of 2013 Korea Health Panel Data. *Journal of Korean Medical Science.* 2017;32(12):1921-1930.
123. Lee GA, Jennings N. A comparative study of patients who did not wait for treatment and those treated by emergency nurse practitioners. *Australasian Emergency Nursing Journal.* 2006;9(4):179-185.
124. Lee KM, Wong TW, Chan R, Lau CC, Fu YK, Fung KH. Accuracy and efficiency of X-ray requests initiated by triage nurses in an accident and emergency department. *Accident and emergency nursing.* 1996;4(4):179-181.
125. Lee MH, Effenberger A. Effect of formation of an accountable care organization on characteristics of emergency department visits. *Academic Emergency Medicine.* 2014;1):S219.
126. Lee WW, Filiatrault L, Abu-Laban RB, Rashidi A, Yau L, Liu N. Effect of triage nurse initiated radiography using the Ottawa Ankle Rules on emergency department length of stay at a tertiary care center. *Canadian Journal of Emergency Medicine.* 2014;16(SUPPL. 1):S38.
127. Lee WW, Filiatrault L, Abu-Laban RB, Rashidi A, Yau L, Liu N. Effect of Triage Nurse Initiated Radiography Using the Ottawa Ankle Rules on Emergency Department Length of Stay at a Tertiary Centre. *CJEM.* 2016;18(2):90-97.
128. Li Y, Lu Q, Du H, Zhang J, Zhang L. The Impact of Triage Nurse-ordered Diagnostic Studies on Pediatric Emergency Department Length of Stay. *Indian journal of pediatrics.* 2018;85(10):849-854.
129. Liferidge A, McCarthy M, Blanchard J, Ding R, Li S. Triage of low acuity emergency department patients to a primary care clinic and medical home: A utilization and cost effectiveness analysis. *Annals of Emergency Medicine.* 2015;1):S87-S88.
130. Lijuan Z. Advanced Triage Protocols in the Emergency Department. *Advanced Triage Protocols in the Emergency Department.* 2017:1-1.
131. Lindley-Jones M, Finlayson BJ. Triage nurse requested x rays--are they worthwhile? *J Accid Emerg Med.* 2000;17(2):103-107.
132. Lindly OJ, Zuckerman KE, Kuhlthau KA. Healthcare access and services use among US children with autism spectrum disorder. *Autism : the international journal of research and practice.* 2018:1362361318815237.
133. Lippi Bruni M, Mammi I, Ugolini C. Does the extension of primary care practice opening hours reduce the use of emergency services? *Journal of Health Economics.* 2016;50:144-155.
134. Long SK, Zuckerman S. Urban health care in transition: Challenges facing Los Angeles County. *Health Care Financing Review.* 1998;20(1):45-58.
135. Love RA, Murphy JA, Lietz TE, Jordan KS. The effectiveness of a provider in triage in the emergency department: a quality improvement initiative to improve patient flow. *Advanced Emergency Nursing Journal.* 2012;34(1):65-74.
136. Lowe RA. Better access to primary care may reduce ED use in Medicaid managed care. *Capitation rates & data.* 2006;11(5):57-60.
137. Lowe RA, Fu R, Ong ET, et al. Community characteristics affecting emergency department use by medicaid enrollees. *Medical Care.* 2009;47(1):15-22.
138. Lukes T, Schjodt K, Struwe L. Implementation of a Nursing Based Order Set: Improved Antibiotic Administration Times for Pediatric ED Patients with Therapy-Induced Neutropenia and Fever. *Journal of Pediatric Nursing.* 2019;46:78-82.
139. Lutze M, Ratchford A, Fry M. A review of the Transitional Emergency Nurse Practitioner. *Australasian Emergency Nursing Journal.* 2011;14(4):226-231.
140. Lynch C, Wajnberg A, Jacob M, et al. Intensive primary care for high readmission risk adults. *Journal of General Internal Medicine.* 2014;1):S488-S489.

For peer review only

141. MacKenzie RS, Burmeister DB, Brown JA, et al. Implementation of a rapid assessment unit (intake team): impact on ED length of stay. *American Journal of Emergency Medicine.* 2015;33(2):291-293.
142. MacKenzie RS, Burmeister DB, Brown JA, et al. Implementation of a rapid assessment unit (intake team): Impact on emergency department length of stay. *Annals of Emergency Medicine.* 2013;1):S12-S13.
143. Mackinney T, Visotcky AM, Tarima S, Whittle J. Does providing care for uninsured patients decrease emergency room visits and hospitalizations? *Journal of Primary Care & Community Health.* 2013;4(2):135-142.
144. Manns BJ, Tonelli M, Zhang J, et al. Enrolment in primary care networks: Impact on outcomes and processes of care for patients with diabetes. *Cmaj.* 2012;184(2):E144-E152.
145. Martin G, McGowan M, Postic M, Gaunt K. Level of patient satisfaction with the NP role in fast track: preliminary findings. *Canadian Journal of Emergency Medicine.* 2013;15 (Suppl 1):S1.
146. Martin M, Girma F, Rushing J. Care plus: Five years of providing team based care to high need high cost patients. *Journal of General Internal Medicine.* 2019;34(2 Supplement):S711.
147. McAlister FA, Bakal JA, Green L, Bahler B, Lewanczuk R. The effect of provider affiliation with a primary care network on emergency department visits and hospital admissions. *CMAJ.* 2018;190(10):E276-E284.
148. McCarron M, Burgess K, Gibbs S. An evaluation of primary care streaming in a tertiary paediatric emergency department. *Archives of Disease in Childhood.* 2019;104(Supplement 2):A138-A139.
149. McClellan C, Cramp F, Powell J, Benger JR. Clinical and cost effectiveness of different emergency department healthcare professionals in the management of musculoskeletal soft tissue injuries. *Physiotherapy (United Kingdom).* 2011;1):eS776-eS777.
150. McDonnell WM, Carpenter P, Jacobsen K, Kadish HA. Relative productivity of nurse practitioner and resident physician care models in the pediatric emergency department. *Pediatric Emergency Care.* 2015;31(2):101-106.
151. Megahy A, Lloyd M. Managing minor injuries. *Emergency nurse : the journal of the RCN Accident and Emergency Nursing Association.* 2004;12(5):14-16.
152. Michael L, Brady AK, Russell G, et al. Connecting Refugees to Medical Homes Through Multi-Sector Collaboration. *Journal of immigrant and minority health.* 2019;21(1):198-203.
153. Miller D, Loftus AM, O'Boyle PJ, et al. Impact of a telephone-first consultation system in general practice. *Postgraduate medical journal.* 2019;95(1129):590-595.
154. Mitchell EA, Didsbury PB, Kruithof N, et al. A randomized controlled trial of an asthma clinical pathway for children in general practice. *Acta Paediatrica, International Journal of Paediatrics.* 2005;94(2):226-233.
155. Mittmann N, Beglaryan H, Liu N, et al. Examination of health system resources and costs associated with transitioning cancer survivors to primary care: A propensity-score-matched cohort study. *Journal of Oncology Practice.* 2018;14(11).
156. Moe J, Oland R, Moe G. Impact of a Primary Care After-Hours Clinic on Avoidable Emergency Department Visits and Costs. *Healthcare quarterly (Toronto, Ont).* 2019;22(1):42-47.
157. Morin C, Choukroun J, Callahan JC. Safety and efficiency of a redirection procedure toward an out of hours general practice before admission to an emergency department, an observational study. *BMC Emergency Medicine.* 2018;18(1):26.
158. Muller K, Chee Z, Doan Q. Using Nurse Practitioners to Optimize Patient Flow in a Pediatric Emergency Department. *Pediatric Emergency Care.* 2018;34(6):396-399.

For peer review only

159. Munday T, Forbes M, Little J, Warren G, Bowe P, Lind J. The patient flow effects of a new medical assessment unit within a large urban health service. *Internal Medicine Journal.* 2012;42 (SUPPL.2):9-10.

160. Murphy AW, Bury G, Plunkett PK, et al. Randomised controlled trial of general practitioner versus usual medical care in an urban accident and emergency department: process, outcome, and comparative cost. *BMJ (clinical research ed).* 1996;312(7039):1135-1142.

161. Musich S, Wang S, Hawkins K, Klemes A. The impact of personalized preventive care on health care quality, utilization, and expenditures. *Population Health Management.* 2016;19(6):389-397.

162. Nash K, Zachariah B, Nitschmann J, Psencik B. Evaluation of the fast track unit of a university emergency department. *JEN: Journal of Emergency Nursing.* 2007;33(1):14-94.

163. Nelson KM, Sylling PW, Taylor L, et al. Improved team function associated with lower health care use. *Journal of General Internal Medicine.* 2016;1):S269.

164. Newman RJ, Bikowski R, Nakayama K, Cunningham T, Acker P, Bradshaw D. Outcomes of Embedded Care Management in a Family Medicine Residency Patient-Centered Medical Home. *Family medicine.* 2017;49(1):46-51.

165. Nguyen ND, Moore JB, McIntosh NP, Jones ML, Zimmerman J, Summers RL. Emergency department triage of low acuity patients to a Federally Qualified Health Center. *Journal of the Mississippi State Medical Association.* 2013;54(10):280-283.

166. Nichols DE, Haber SG, Romaire MA, Wensky SG, Multi-Payer Advanced Primary Care Practice Evaluation T. Changes in Utilization and Expenditures for Medicare Beneficiaries in Patient-centered Medical Homes: Findings From the Multi-Payer Advanced Primary Care Practice Demonstration. *Medical care.* 2018;56(9):775-783.

167. Nyweide DJ, Bynum JPW. Relationship Between Continuity of Ambulatory Care and Risk of Emergency Department Episodes Among Older Adults. *Annals of Emergency Medicine.* 2017;69(4):407-415.e403.

168. O'Keeffe N. The effect of a new general practice out-of-hours co-operative on a county hospital accident and emergency department. *Irish journal of medical science.* 2008;177(4):367-370.

169. O'Kelly FD, Teljeur C, Carter I, Plunkett PK. Impact of a GP cooperative on lower acuity emergency department attendances. *Emergency Medicine Journal.* 2010;27(10):770-773.

170. Olsen JC, Ogarek J, Goldenberg E, Sulo S. Effect of a chronic pain protocol on emergency department utilization. *Academic Emergency Medicine.* 2015;1):S151.

171. Organ K, Chinnick P, Higgison I, Stanhope B, Hoskins R, Benger J. Evaluating the introduction of a paediatric emergency nurse practitioner service. *Emergency nurse : the journal of the RCN Accident and Emergency Nursing Association.* 2005;13(7):8-11.

172. O'Toole TP, Buckel L, Bourgault C, et al. Applying the chronic care model to homeless veterans: effect of a population approach to primary care on utilization and clinical outcomes. *American journal of public health.* 2010;100(12):2493-2499.

173. Owens D, Eby K, Burson S, Green M, McGoodwin W, Isaac M. Primary Palliative Care Clinic Pilot Project demonstrates benefits of a nurse practitioner-directed clinic providing primary and palliative care. *Journal of the American Academy of Nurse Practitioners.* 2012;24(1):52-58.

174. Parris W, McCarthy S, Kelly AM, Richardson S. Do triage nurse-initiated X-rays for limb injuries reduce patient transit time? *Accid Emerg Nurs.* 1997;5(1):14-15.

175. Parsons PL, McMurtry CT. NP care/discharge planning saves money. *The Nurse practitioner.* 1997;22(3):238-240.

For peer review only

176. Patel H, Celenza A, Watters T. Effect of nurse initiated X-rays of the lower limb on patient transit time through the emergency department. *Australasian Emergency Nursing Journal.* 2012;15(4):229-234.

177. Peterson GG, Geonnotti KL, Hula L, et al. Association between extending carefirst's medical home program to medicare patients and quality of care, utilization, and spending. *JAMA Internal Medicine.* 2017;177(9):1334-1342.

178. Philips H, Remmen R, Van Royen P, et al. What's the effect of the implementation of general practitioner cooperatives on caseload? Prospective intervention study on primary and secondary care. *BMC Health Services Research.* 2010;10:222.

179. Phillips RL, Bronnikov S, Petterson S, et al. Case study of a primary care-based accountable care system approach to medical home transformation. *Journal of Ambulatory Care Management.* 2011;34(1):67-77.

180. Pickin DM, O'Cathain A, Fall M, Morgan AB, Howe A, Nicholl JP. The impact of a general practice co-operative on accident and emergency services, patient satisfaction and GP satisfaction. *Family Practice.* 2004;21(2):180-182.

181. Pierce BA, Gormley D. Are Split Flow and Provider in Triage Models in the Emergency Department Effective in Reducing Discharge Length of Stay? *Journal of Emergency Nursing.* 2016;42(6):487-491.

182. Pirraglia PA, Rowland E, Wu WC, et al. Benefits of a primary care clinic co-located and integrated in a mental health setting for veterans with serious mental illness. *Preventing chronic disease.* 2012;9:E51.

183. Platter MEM, Kurvers RAJ, Janssen L, Verweij MMJ, Barten DG. The impact of an emergency care access point on pediatric attendances at the emergency department: An observational study. *The American journal of emergency medicine.* 2019.

184. Prescott C, Stackhouse N. Developing an advanced nurse practitioner approach to clinical assessments. *Emergency Nurse.* 2017;24(9):33-37.

185. Preston-Suni K, Fleischman R, Ramon J, et al. Triage improvements reduce wait times and eliminate disparities for patients with limited english proficiency. *Academic Emergency Medicine.* 2019;26(Supplement 1):S84.

186. Raffetto B, Balingit P. Safety net hospitals: Are medical homes the answer to overcrowding emergency departments? *Academic Emergency Medicine.* 2014;1):S221.

187. Raphael JL, Cooley WC, Vega A, et al. Outcomes for Children with Chronic Conditions Associated with Parent- and Provider-reported Measures of the Medical Home. *Journal of health care for the poor and underserved.* 2015;26(2):358-376.

188. Rautava V, Valpas T, Nurmikari M, Palomaki A. Establishing a new emergency department: Effects on patient flow. *Critical Care.* 2013;2):S97.

189. Ravichandran Y, Parker SJ, Mowbray FI, et al. Examining utility of advanced practice providers for asthma management in a pediatric emergency department. *Academic Emergency Medicine.* 2019;26(Supplement 1):S298.

190. Ray M, Reinoso H. A New Process to Improve Throughput in the Emergency Department. *Journal for Nurse Practitioners.* 2019;15(10):e193-e196.

191. Reich H, Nwogu N, Garcia-Jimenez MD, et al. Ambulatory care visits increase emergency room visits and admissions in an urban, resident-run clinic. *Journal of General Internal Medicine.* 2018;33 (2 Supplement 1):109.

192. Retezar R, Bessman E, Ding R, Zeger SL, McCarthy ML. The effect of triage diagnostic standing orders on emergency department treatment time. *Annals of emergency medicine.* 2011;57(2):89-99.e82.

For peer review only

193. Rhee KJ, Dermyer AL. Patient satisfaction with a nurse practitioner in a university emergency service. *Annals of Emergency Medicine.* 1995;26(2):130-132.
194. Rivara RP, Wall HP, Worley P, James KD. Pediatric nurse triage: Its efficacy, safety, and implications for care. *American Journal of Diseases of Children.* 1986;140(3):205-210.
195. Riverin BD, Li P, Naimi AI, Strumpf E. Team-based versus traditional primary care models and short-term outcomes after hospital discharge. *Cmaj.* 2017;189(16):E585-E593.
196. Rogers T, Ross N, Spooner D. Evaluation of a 'see and treat' pilot study introduced to an emergency department. *Accident and Emergency Nursing.* 2004;12(1):24-27.
197. Rosenthal MB, Alidina S, Friedberg MW, et al. A Difference-in-Difference Analysis of Changes in Quality, Utilization and Cost Following the Colorado Multi-Payer Patient-Centered Medical Home Pilot. *Journal of General Internal Medicine.* 2016;31(3):289-296.
198. Rosenthal MB, Friedberg MW, Singer SJ, Eastman D, Li Z, Schneider EC. Effect of a multipayer patient-centered medical home on health care utilization and quality: The rhode island chronic care sustainability initiative pilot program. *JAMA Internal Medicine.* 2013;173(20):1907-1913.
199. Rothkopf J, Brookler K, Wadhwa S, Sajovetz M. Medicaid patients seen at federally qualified health centers use hospital services less than those seen by private providers. *Health Affairs.* 2011;30(7):1335-1342.
200. Rothwell S, McIltrot K, Khouri-Stevens Z. Addressing Emergency Department Issues Using Advanced Practice in Saudi Arabia. *Journal for Nurse Practitioners.* 2018;14(2):e41-e44.
201. Rust G, Baltrus P, Ye J, et al. Presence of a community health center and uninsured emergency department visit rates in rural counties. *Journal of Rural Health.* 2009;25(1):8-16.
202. Rutten MH, Smits M, Peters YAS, Assendelft WJJ, Westert GP, Giesen PHJ. Effects of access to radiology in out-of-hours primary care in the Netherlands: a prospective observational study. *Family Practice.* 2018;35(3):253-258.
203. Salisbury C, Hollinghurst S, Montgomery A, et al. The impact of co-located NHS walk-in centres on emergency departments. *Emergency Medicine Journal.* 2007;24(4):265-269.
204. Sanchez M, Smally AJ, Grant RJ, Jacobs LM. Effects of a fast-track area on emergency department performance. *J Emerg Med.* 2006;31(1):117-120.
205. Savage AI, Lauby T, Burkard JF. Examining selected patient outcomes and staff satisfaction in a primary care clinic at a military treatment facility after implementation of the patient-centered medical home. *Military medicine.* 2013;178(2):128-134.
206. Schmidt EK, Hess A, Hanks C, Hand B. A Patient-Centered Medical Home for Young Adults with Autism and Subsequent Emergency Department Utilization. *Archives of Physical Medicine and Rehabilitation.* 2019;100(10):e35-e36.
207. Schwartzman K, Duquette G, Zaoude M, et al. Respiratory day hospital: A novel approach to acute respiratory care. *Cmaj.* 2001;165(8):1067-1071.
208. Seaberg DC, Hennings J, Good M, et al. Redirecting low-acuity pediatric emergency department patients to a hospital-based federally qualified health center. *Academic Emergency Medicine.* 2010;1):S96.
209. Sharma A, Inder B. Impact of co-located general practitioner (GP) clinics and patient choice on duration of wait in the emergency department. *Emergency Medicine Journal.* 2011;28(8):658-661.
210. Shea SS, Hoyt KS. "RAPID" Team triage: One hospitals approach to patient-centered team triage. *Advanced Emergency Nursing Journal.* 2012;34(2):177-189.
211. Sheikh S, Pitts J, Ryan-Wenger N, Hayes D, McCoy K. Impact of type of health-care provider on long-term asthma care in children: A model for primary care. *Chest.* 2016;150 (4 Supplement 1):886A.

For peer review only

212. Shrimpling M. Redesigning triage to reduce waiting times. *Emerg Nurse.* 2002;10(2):34-37.
213. Shumacher RJ, Meyer J, Johnson B, Grisham T, Chamberlain L, Jennings J. Clinical impact of optumTM careplus on nursing homes and residents. *Journal of the American Geriatrics Society.* 2014;1):S15.
214. Sikkenga T, Dumkow L, Draper H, et al. Implementation of a nursing triage order to improve utilization of rapid diagnostic testing for chlamydia and gonorrhea in the emergency department. *Open Forum Infectious Diseases.* 2016;3(Supplement 1).
215. Sinclair L, Hunter R, Hagen S, Nelson D, Hunt J. How effective are mental health nurses in A&E departments? *Emergency medicine journal.* 2006;23(9):687-692.
216. Sjonell G. Effect of establishing a primary health care center on the utilization of primary health care and other out-patient care in a Swedish urban area. *Family Practice.* 1986;3(3):148-154.
217. Sledge WH, Brown KE, Levine JM, et al. A randomized trial of primary intensive care to reduce hospital admissions in patients with high utilization of inpatient services. *Disease Management.* 2006;9(6):328-338.
218. Smith JJ, Johnston JM, Hiratsuka VY, Dillard DA, Tierney S, Driscoll DL. Medical home implementation and trends in diabetes quality measures for AN/AI primary care patients. *Primary Care Diabetes.* 2015;9(2):120-126.
219. Smith L, Narang Y, Ibarz Pavon AB, et al. To GP or not to GP: a natural experiment in children triaged to see a GP in a tertiary paediatric emergency department (ED). *BMJ Quality & Safety.* 2018;27(7):521-528.
220. Smith-Campbell B. Emergency department and community health center visits and costs in an uninsured population. *Journal of nursing scholarship : an official publication of Sigma Theta Tau International Honor Society of Nursing / Sigma Theta Tau.* 2005;37(1):80-86.
221. Sprandio JD. Oncology patient-centered medical home and accountable cancer care. *Community Oncology.* 2010;7(12):565-572.
222. Steiner IP, Nichols DN, Blitz S, et al. Impact of a nurse practitioner on patient care in a Canadian emergency department. *CJEM Canadian Journal of Emergency Medical Care.* 2009;11(3):207-214.
223. Sterner SE, Coco T, Monroe KW, King WD, Losek JD. A new after-hours clinic model provides cost-saving, faster care compared with a pediatric emergency department. *Pediatric Emergency Care.* 2012;28(11):1162-1165.
224. Stoddart D, Ireland AJ, Crawford R, Kelly B. Impact on an accident and emergency department of Glasgow's new primary care emergency service. *Health bulletin.* 1999;57(3):186-191.
225. Stone M, Nguygen ND, Moore JB, et al. Emergency department triage of low acuity patients to a federally qualified health center. *Annals of Emergency Medicine.* 2013;1):S46-S47.
226. Strange P. The nurse practitioner in A&E. *Australian nursing journal (July 1993).* 1994;1(9):20-23.
227. Subash F, Dunn F, McNicholl B, Marlow J. Team triage improves emergency department efficiency. *Emergency medicine journal.* 2004;21(5):542-544.
228. Sukpraprut-Braaten S, Henderson JC, Kinchen DL, et al. Medical screening exam utilized to manage emergent and non-emergent patients presenting to the emergency department. *Annals of Emergency Medicine.* 2016;68 (4 Supplement 1):S71.
229. Sung NJ, Choi YJ, Lee JH. Primary Care Comprehensiveness Can Reduce Emergency Department Visits and Hospitalization in People with Hypertension in South Korea. *International Journal of Environmental Research & Public Health [Electronic Resource].* 2018;15(2):05.

230. Terp S, Mehta R, Arora S, Goldman D, Romley J, Menchine M. Evaluating the association between primary care provider availability and ed visits and hospitalizations for ambulatory care sensitive conditions in California. *Academic Emergency Medicine.* 2013;1):S295.
231. Thijssen W, Kraaijvanger N, Barten DG, Boerma MLM, Giesen P, Wensing M. Impact of a well-developed primary care system on the length of stay in emergency departments in the Netherlands: a multicenter study. *BMC Health Services Research.* 2016;16:149.
232. Thijssen WA, Wijnen-van Houts M, Koetsenruijter J, Giesen P, Wensing M. The impact on emergency department utilization and patient flows after integrating with a general practitioner cooperative: an observational study. *Emergency Medicine International Print.* 2013;2013:364659.
233. Thompson K, Etheridge L. The impact of children's ambulatory unit model of care on children's emergency department flow. *Archives of Disease in Childhood.* 2019;104(Supplement 2):A129.
234. Thompson W, Meskell P. Evaluation of an Advanced Nurse Practitioner (Emergency Care)—An Irish Perspective. *Journal for Nurse Practitioners.* 2012;8(3):200-205.
235. Thrasher C, Purc-Stephenson R. Patient satisfaction with nurse practitioner care in emergency departments in Canada. *Journal of the American Academy of Nurse Practitioners.* 2008;20(5):231-237.
236. Thurston J, Field S. Should accident and emergency nurses request radiographs? Results of a multicentre evaluation. *J Accid Emerg Med.* 1996;13(2):86-89.
237. Timbie JW, Setodji CM, Kress A, et al. Implementation of medical homes in Federally Qualified Health Centers. *New England Journal of Medicine.* 2017;377(3):246-256.
238. To T, Guan J, Zhu J, et al. Quality of asthma care under different primary care models in Canada: a population-based study. *BMC family practice.* 2015;16:19.
239. Traub SJ, Wood JP, Kelley J, et al. Emergency department rapid medical assessment: overall effect and mechanistic considerations. *Journal of Emergency Medicine.* 2015;48(5):620-627.
240. Tsai MH, Xirasagar S, Carroll S, et al. Reducing High-Users' Visits to the Emergency Department by a Primary Care Intervention for the Uninsured: A Retrospective Study. *Inquiry : a journal of medical care organization, provision and financing.* 2018;55:46958018763917.
241. Tsai V, Harley J, Sharieff G, Carlson LA, Kanegaye J. Rapid medical assessment: Improved patient flow and left without being seen rates. *Pediatric Emergency Care.* 2009;25 (10):706.
242. Tsai VW, Sharieff GQ, Kanegaye JT, Carlson LA, Harley J. Rapid medical assessment: improving pediatric emergency department time to provider, length of stay, and left without being seen rates. *Pediatric Emergency Care.* 2012;28(4):354-356.
243. Tucker A, Bernard M. Making the Case for Nurse Practitioners in the Emergency Department: A Clinical Case Study. *Advanced Emergency Nursing Journal.* 2015;37(4):308-312.
244. Tucker S, Jiang R, Beharry A, Raman A, Jubelt LE. Turn no patient away: Adopting advanced access scheduling in primary care. *Journal of General Internal Medicine.* 2018;33(2 Supplement 1):839-840.
245. Uthman OA, Walker C, Lahiri S, et al. General practitioners providing non-urgent care in emergency department: a natural experiment. *BMJ Open.* 2018;8(5):e019736.
246. van den Bersselaar DLCM, Maas M, Thijssen WAMH. Does X-ray imaging by GPC at emergency care access points in the Netherlands change patient flow and reduce ED crowding? A cohort study. *Health science reports.* 2018;1(2):e26.
247. van der Linden C, Reijnen R, de Vos R. Diagnostic accuracy of emergency nurse practitioners versus physicians related to minor illnesses and injuries. *Journal of Emergency Nursing.* 2010;36(4):311-316.

For peer review only

248. van Gils-van Rooij ESJ, Meijboom BR, Broekman SM, Yzermans CJ, de Bakker DH. Is patient flow more efficient in Urgent Care Collaborations? *European Journal of Emergency Medicine.* 2018;25(1):58-64.
249. Van Uden CJT, Ament AJHA, Voss GBWE, et al. Out-of-hours primary care. Implications of organisation on costs. *BMC Family Practice.* 2006;7(no pagination).
250. Van Uden CJT, Winkens RAG, Wesseling GJ, Crebolder HFJM, Van Schayck CP. Use of out of hours services: A comparison between two organisations. *Emergency Medicine Journal.* 2003;20(2):184-187.
251. van Veelen MJ, van den Brand CL, Reijnen R, van der Linden MC. Effects of a general practitioner cooperative co-located with an emergency department on patient throughput. *World journal of emergency medicine.* 2016;7(4):270-273.
252. Vinton D, Riordan JP, O'Conner R, Joseph JW. Impact of the management of low-acuity patients at triage on their length of stay. *Academic Emergency Medicine.* 2019;26(Supplement 1):S235-S236.
253. Voelker J, Hegarty SE, Keith SW, Alcusky M, Lombardi M, Maio V. Do medical homes affect healthcare utilization rates? a longitudinal evaluation on medical home implementation in Italy. *Value in Health.* 2018;21(Supplement 1):S133.
254. Wallis M, Marsden E, Taylor A, et al. The Geriatric Emergency Department Intervention model of care: a pragmatic trial. *BMC geriatrics.* 2018;18(1):297.
255. Wand T, D'Abrew N, Acret L, White K. Evaluating a new model of nurse-led emergency department mental health care in Australia; perspectives of key informants. *International emergency nursing.* 2016;24:16-21.
256. Wand T, D'Abrew N, Barnett C, Acret L, White K. Evaluation of a nurse practitioner-led extended hours mental health liaison nurse service based in the emergency department. *Australian Health Review.* 2015;39(1):1-8.
257. Wang M, Wild S, Hilfiker G, et al. Hospital-integrated general practice: a promising way to manage walk-in patients in emergency departments. *Journal of Evaluation in Clinical Practice.* 2014;20(1):20-26.
258. Watts B, Lawrence RH, Carter C, et al. Does intensive ambulatory management of high risk patients reduce acute care utilization? a pilot study. *Journal of General Internal Medicine.* 2016;1):S199.
259. Weinberger M, Oddone EZ, Henderson WG. Does increased access to primary care reduce hospital readmissions? *New England Journal of Medicine.* 1996;334(22):1441-1447.
260. Wilson A, Shifaza F. An evaluation of the effectiveness and acceptability of nurse practitioners in an adult emergency department. *International Journal of Nursing Practice.* 2008;14(2):149-156.
261. Winston S. Nurse practitioners in the emergency department: a case study of the Washington Hospital Center, Washington, D.C. *Hosp Top.* 1981;59(4):10-13.
262. Wright B, Potter AJ, Trivedi AN, Mueller KJ. The Relationship Between Rural Health Clinic Use and Potentially Preventable Hospitalizations and Emergency Department Visits Among Medicare Beneficiaries. *The Journal of rural health : official journal of the American Rural Health Association and the National Rural Health Care Association.* 2018;34(4):423-430.
263. Wright DB, Ricketts TC. The road to efficiency? Re-examining the impact of the primary care physician workforce on health care utilization rates. *Social Science and Medicine.* 2010;70(12):2006-2010.
264. Wright SW, Erwin TL, Blanton DM, Covington CM. Fast Track in the emergency department: a one-year experience with nurse practitioners. *Journal of Emergency Medicine.* 1992;10(3):367-373.

For peer review only

265. Wu FM, Rubenstein LV, Yoon J. Team functioning as a predictor of patient outcomes in early medical home implementation. *Health care management review.* 2018;43(3):238-248.

266. Xin H, Kilgore ML, Menachemi N, Sen B. The relationships between access to and use of a patient-centered medical home and healthcare utilization and costs: A cohort study using Medical Expenditure Panel Survey data from 2007 to 2010. *Health Services Management Research.* 2014;27(3-4):70-81.

267. Yuan X, Guh S, Hochheiser D, Mayo T, Nigam S. Incremental effect of quality blue practices on disease management in Louisiana. *Value in Health.* 2018;21(Supplement 1):S136-S137.

268. Zager K, Taylor YJ. Discharge to medical home: A new care delivery model to treat non-urgent cases in a rural emergency department. *Healthcare.* 2018;21:21.

For peer review only

Appendix Table 5: Countries in which studies were conducted

Countries	n (N=268)	Percentage (%)
**USA**	126	47
**UK**	43	16
**Canada**	30	11.2
**Australia**	20	7.5
**Netherlands**	13	4.8
**Switzerland**	5	1.9
**Sweden**	4	1.5
**France**	4	1.5
**Italy**	3	1
**South Korea**	3	1
**China**	3	1
**New Zealand**	2	0.8
**Saudi Arabia**	2	0.8
**Taiwan**	2	0.8
**Belgium**	1	0.4
**Brazil**	1	0.4
**Finland**	1	0.4
**Oman**	1	0.4
**Portugal**	1	0.4
**Spain**	1	0.4
**Singapore**	1	0.4
**Not reported**	1	0.4

For peer review only

Appendix Table 6: Types of interventions reported by the included studies

Within ED<br>Interventions or strategies	Outside ED<br>Interventions or strategies
**1. Area within ED staffed by PHCPs - to manage ED patients streamlined at triage**	**1. Improving timely access to primary care**
Minor injuries unit – NP assess, diagnose, treat, and discharge within agreed set of protocols	GP Cooperatives able to request X-rays in the evenings
Low acuity 4-bed ED triage area	GP Cooperatives - re-organised 100 GPs in the region and centralised the location for out-of-hours primary health care in one centrally located practice
NP in ED treat low acuity patients	Increasing number of GPs per 1000 patients
NP in fast track area	After-hour primary care practices (12 or more evening hours per week)
Rapid Medical Assessment – NP assess, diagnose, discharge low-acuity patients	High team functioning of primary care practices
**2. PHCPs located next to ED (sharing triage with ED) to manage ED patients streamed at triage and self-directed patients**	7-day opening of GP practices
The Emergency Care Access Point (ECAP) - a collaboration between a GP cooperative (GPC) and the ED	Transitioning low-risk cancer survivors back to their primary care provider (PCP) to decrease ED utilization
Walk-in centers co-located with ED	Advanced access to primary care (provide timely access to scheduled appointments, accommodate requests for same day access, understand facilitators and barriers to promoting uptake)
**3. PHCPs located at ED triage to manage patients**	High primary care access (95-100%)- ability for patients to schedule GP appointments
Advanced nurse practitioners-led rapid assessment triage (RAT)	Shared medical appointments (SMAs) - health care providers care for multiple patients with similar

For peer review only

	health conditions in a supportive, educational environment
Combined nurse and provider at triage	Los Angeles County Department of Health Services (LACDHS) - restructuring to improve access to and efficiency for providing ambulatory care and preventive services to Medicaid and uninsured populations
Paediatric "see-and-treat" service	Telephone-first consultation system - GPs were available for telephone consultations
Diversion of non-urgent patients to primary care	Same-day physician access in long-term care homes
Advanced practice providers (NP) in triage	**2. Integrating hospital and GP patient care**
Discharge to primary care with same day appointment	Hospital integrated general practice for emergency care services (HGP) (based on a team of GPs and emergency staff physicians) - HGPs are located within the hospital, sharing the same access point as the ED, as well as some infrastructure
Triage nurse order set	Evening clinics and walk-in centers within hospital campus located fairly close to, but outside ED
ED triage nurses applying Ottawa ankle, foot and knee rules	Hospital associated primary care unit with GPs (H-GP-unit as a fast track)
Split flow model of care combined with provider in triage	Primary care visits and clinic visits for all medical and surgical sub-specialities
Brief screening exam and tests order by triage nurse	Integrated emergency posts (IEPs) - integrating the care provided by A&E departments and GP posts
Triage nurse Renal Colic Fast Track pathway	Co-located GP clinics - low acuity patients are diverted from the ED to attend these clinics
Full triage Standing Orders	Emergency Care Access Point's (ECAP) - EDs and GPs work together. Triage decides if the patient will be seen by a GP or in the ED. To redirect non-urgent

For peer review only

	self-referrals to GP. GP functions as a gatekeeper for ED visits.
Nurse triage order to initiate the CT and NG rapid testing	Urgent Care Collaborations (UCCs) - out of hours GP sharing one combined entrance and joint triage with the ED
Nurse-initiated analgesia protocol	Integrated GP cooperatives - GP cooperative is located at the site of the hospital ED to ensure self-referred patients cannot enter ED without first having been seen by a GP
Nurse-initiated chest pain protocol	Integrating Primary Care into Community Mental Health Centers
Triage Nurse Counselling	Primary Care Clinic Co-Located and Integrated in a Mental Health Setting
Nurse-initiated suspected fracture protocol	Mobile unit - located outside the main ED staffed by a registered nurse, a NP and a technician who floats between the unit and the main ED with an ED physician available for consultation as needed.
Nurse-initiated vaginal bleeding during pregnancy protocol	Primary Care Hub - Patients presenting to ED are streamed to the hub provided they do not meet any of the set exclusion criteria.
Nurse-initiated lower abdominal pain protocol	**3. Providing financial support**
Nurse-initiated upper abdominal pain	Adult walk-in primary care clinic on-campus, free of cost for uninsured patients under 200% of poverty and to actively urge insured ED high-users to either acquire a primary care physician if they did not have one, or regularly visit their existing PCP
Triage nurse-initiated X-ray	Citicare program - The GP (prospective capitation) was required to be available or provide physician coverage 24 hours a day, seven days a week, and functioned as a gatekeeper to all medical services

For peer review only

See-and-treat minor injury illnesses and injuries (NP + physician)	Primary care provider blended fee-for-service
**4. PHCPs fully integrated with the ED to manage ED patients**	**4. Implementing new clinics/services**
NP interpret X-rays	Patient centered medical home model – Team based primary care delivery and coordination with providers across the healthcare system
Asthma management for pediatric patients by advanced practice providers in ED	Primary health care provided by Nurse Practitioner - in centers for Medicare and Medicaid Services
GP added to the ED	GPs affiliated with newly established medical homes (MHs)
NP added to the ED staff during busy hours	Milwaukee Cares - provided uninsured patients with insurance and connected patients with free primary care
Registered nurse advises on appropriate care sources for non-urgent patients	Federally Qualify Health Centers (FQHC)- low acuity patients were scheduled a same day clinic appointment with a local FQHC that functions as "safety net" providers
Extended hours mental health liaison nurse (MHLN) service	Community Health Centers in rural counties in Georgia - access to a primary care medical home for the uninsured in rural communities with no other primary care safety net to potentially reduce uninsured ED visits
Pediatric trauma NP care	Clinic-based ambulatory case management intervention (Primary Intensive Care) by NP, to reduce hospital utilization
Nurse-led Chest pain service to risk-stratify	After-hours clinic - an appointment-only clinic where patients must contact their primary care physician or the children’s hospital’s Pediatric Health Information

For peer review only

	Line (telephone triage) to be scheduled for same-day visits
NP-led extended hours mental health liaison nurse (MHLN) service embedded within the ED	GP-led walk-in centres
Public education campaign within ED to patients on proper use of Eds and directing them to appropriate care	Primary Behavioral Health Care Integration (PBHCI) program – Physical and mental health clinics integrated
Chronic pain protocol - family physician and ED physician	Family Medicine Groups (FMG) - intended to include six to ten physicians and two nurses to serve a population of 10,000 to 20,000 patients, longer hours (weekdays, weekends, and holidays) and on-call services for vulnerable patients
Pediatric flow nurse - collaborates with ED nursing and medical staff to start treating patients and to help move children from the ED to the paediatric emergency short stay unit	Computerised Spanish guideline on the management of asthma (GEMA) in 7 GP clinics and a collaborative care environment between GP and Pulmonology department
Nurse triage and GP-led emergency service	Easy Breathing is an asthma management program for primary care clinicians
Transitional emergency nurse practitioner model	ED physicians have the option of scheduling follow-up appointments in a GP clinic
Geriatric Emergency Department Intervention (GEDI) model of service – NP In consultation, with the ED physician will undertake targeted geriatric assessment and problem formulation. They will then work with the primary ED team to fast-track diagnostic process and decision-making	Free clinic - offers general medical care, pregnancy testing, gynecology/birth control, immunizations, chronic disease management, and links to medication assistance
	Large primary-care based accountable care organizations (ACO) (both a Medicare and private insurer-sponsored plan)
	Internal Medicine Associates- Preventable Admissions Care Team (IMA/PACT) primary care clinic

For peer review only

	Asthma clinical pathway intervention - Each GP was given a 2-h group education session (algorithms for management of acute and chronic asthma)
	MD-Value in Prevention (MDVIP) - personalized preventive health care via annual 60- to 90-minute appointment that includes health screenings, diagnostics, personalized coaching and online tools for nutrition and exercise for a membership fee
	Population-tailored primary care clinic - hospital-based, open-access care model with nurse case management and wraparound onsite services
	The Geriatric Speciality Clinic (GSC) - interdisciplinary primary care geriatric practice with both NPs and geriatricians as primary care providers
	Extending CareFirst's Medical Home Program - nurses work with patients' usual primary care practitioners to coordinate care for high-risk Medicare patients
	WellMed - Each patient has a primary care clinician who coordinates all care and refers to specialists as needed
	Pediatric asthma centre - Patients were seen by either physicians or ANP (Advanced Practice Nurse)
	Optum Care Plus - Program that provides onsite care to nursing homes residents along with the resident's primary care physician and nursing home staff
	Medical Home practices - GP affiliated with the newly established medical homes
	Intensive primary care intervention - The intervention involved close follow-up by a nurse or a primary care

For peer review only

	physician beginning before discharge and continuing for the next 6 months.
	Center for Autism Services and Transition (CAST) - primary care center designed for adults with autism
	Family Health Units (FHU)
	Rural Health Clinics - located in a rural health professional shortage area and provide primary care and laboratory services
	Refugee Health Collaborative - a Collaborative-developed novel algorithm guided the process by which refugees establish care in Patient centered medical homes
	Team-based primary care and care coordination for high need high cost patients from academic safety net practices
	Children's and Young People's Ambulatory Unit (CAU) - ambulatory care model with limited opening hours
	Pediatric primary care practices
	Coordinated Care Center (CCC) - An intensive primary care clinic (NPs and nurses), created to address the unique needs of high-needs and high-cost patients

For peer review only

Appendix Table 7: Study design, population and the interventions reported by the included studies.

Author, Year	Study design	Study population	Intervention
Lindly 2018	Cross-sectional study	Children with autism spectrum disorder	Improved GP accessibility
Platter 2019	Retrospective cohort	All patients under the age of 18 years who visited the ED during the two testing periods were included.	Emergency care Access Point
Bottle 2018	Retrospective cohort	All patients admitted to acute non-specialist hospitals as an emergency for HF or COPD	Higher number of GPs per 1000 patients
Anantharaman 2008	Retrospective cohort	Patients who presented to the ED	Redirection of non-urgent patients
Lowe 2006	Cohort study	Patients under the age of 65 were included in the study if they were enrolled in the HMO and assigned to a study practice at any time between August 1, 1998, and July 31, 1999	Primary care practices with 12 or more evening hours
van der Bersselaar 2018	Retrospective cohort	All patients that visited an ECAP and had an X-ray imaging requested by the GP at the GP cooperative	GP cooperatives
Nichols 2018	Retrospective cohort	Medicare FFS beneficiaries	Patient-centered medical home
Kuo 2018	Retrospective cohort	Patients with congestive heart failure	PCP care model
Gardner 2018	Before-and-after study	Adult patients who were not likely to require intensive evaluations.	Reallocating NP into triage area

For peer review only

Carter 2014	Pre-post study	Patients seeking care at ED	GP ambulatory Care Unit
Ravichadran 2019	Retrospective chart review	Patients aged 2 to 18 years, discharged with diagnostic code for asthma	NP-led asthma management
Vinton 2019	Before-and-after study	Patient population consisted of ESI level 4's and 5's who were treated and discharged from within the ED intake area between the hours of 11a-11p from 11/19/2018-12/29/2018	Treat and discharge low-acuity patients at triage
Preston-Suni 2019	Before-and-after study	Patients attending ED, with lower English proficiency	Combined nurse and provider triage intervention
Chmiel 2016	Interrupted time series	Adults visiting ED	Hospital integrated general practice
Clancy 2009	Prospective cohort	Peds patients with minor injuries or illnesses	Pediatric see-and-treat service
Colligan 2011	Prospective cohort	Adults visiting ED with trauma	Nurse practitioners who treated lower acuity injuries
Wu 2018	Retrospective cohort study	Veterans who attended 15 primary care practices in one Veterans Health Administration (VHA) administrative region that began implementation of Patient-Aligned Care Team (PACT) in 2010.	Primary care practices with high team functioning
Considine 2008	Pre-post study	Patients visiting to the ED	Fast Track system for non-urgent patients

For peer review only

Dahrouge 2016	Cross-sectional study	Patients with valid health care coverage (OHIP)	GP providing care to a larger number of patients
Jones 2011	Mixed-methods study	Any patient visiting ED	GP added to the ED - seeing and treating minor cases
Tsai 2018	Retrospective cohort study	ED High-users	Free adult walk-in primary care clinic for uninsured
Kern 2019	Retrospective cohort study	Adult (aged ≥ 18 years) Medicaid beneficiaries	Low level of fragmented health care
Dias 2016	Cohort study	Patients presenting to the ED	Provider refusing nonurgent patients
Dinh 2012	Randomized controlled trial	Patients between age 16 and 70 years presenting to the emergency department	Fast Track unit by NP
Dolton 2016	Retrospective cohort	Patients attending a GP service	7-day opening of GP practices
Ducharme 2009	Pre-post study	Any patient visiting the ED	Addition of NP
Edwards 2011	Prospective cohort	Any patient visiting the ED	NP rapid assessment team
Kao 2019	Retrospective cohort study	Patients aged ≥ 65 years who either had at least 2 outpatient visits or 1 inpatient admission for COPD as a primary diagnosis, identified during the period from January 1, 2005, to December 31, 2011.	Better continuity of care

For peer review only

Eichler 2014	Interrupted time series	Any patient visiting the ED	Hospital associated primary care unit with GP
Buerhaus 2018	Retrospective cohort study	Patients of all ages, disabled, and dual Medicaid and Medicare beneficiaries	Primary health care provided by NP
Mittmann 2018	Propensity-Score Matched Cohort Study	Breast cancer survivors	PCP care for breast cancer survivors
Crits-Cristoph 2018	Before-and-after study	HIV-positive Medicaid patients with chronic conditions	Patient-centered medical home
Chang 2018	Case-control study	Patients who had avoidable ED visit between 2010 and 2013 was selected as the case-group in this study and were matched to patients who had outpatient visits but without any avoidable ED visit in the same year.	Higher continuity of care
Kern 2018	Retrospective cohort study	Medicare beneficiaries 65 years and older could be attributed to those primary care physicians who had billing zip codes in the Hudson Valley. Patients were identified according to the number of ambulatory visits in a year.	Least fragmented care
Hansagi 1990	Randomized controlled trial	Patients seeking care at the ED	Advice and re-direction of low acuity patients by nurse
Hayden 2014	Pre-post study	All adult and pediatric triage level 3 ESI patients during the emergency department's busiest hours	NP in triage

For peer review only

Hearld 2018	Cohort study	Patients with depression	Patient-centered medical home
Hearld 2012	Cross-sectional study	Patients 18 years of age or older, with one of five chronic diseases: asthma, coronary artery disease, depression, diabetes, and hypertension	Improving care coordination
Voelker 2018	Cohort study	Patients ≥ 14 years residing in Parma ≥ 1 year prior to entry in the study	Assignment to a GP affiliated to a Medical Home
Hearld 2017	Retrospective cohort	Patients of primary care and specialty care physician practices that were members of a consortium of physician organizations participating in the Blue Cross Blue Shield of Michigan's Physician Group Incentive Program	Physician practices with high capacities
Yuan 2018	Cohort study	Patients ≥ 18 years, including those with chronic diseases	Quality Blue program - provides primary care
McAlister 2018	Retrospective cohort study	All Albertans aged 20 years or older who were seen by a primary care physician at least once in fiscal years 2008 or 2009.	Patient-centered medical home
Jeanmonod 2013	Retrospective cohort	Low acuity patients	NP care in the ED
Tucker 2018	Before-and-after study	Patients attending an advanced access model at a union-sponsored worksite clinic	Advanced access to primary care
Lagisetty 2018	Retrospective cohort study	Chronic opioid users ages 18-64 years undergoing major or minor surgery	Having consistent PCP

For peer review only

Reich 2018	Retrospective cohort study	Patients attending the resident-run primary care clinic	Primary care visits for all medical and surgical subspecialities
Kim 2017	Cross-sectional study	Adults with current asthma	Increased availability of PCP
Kool 2008	Case-control study	Patients presenting to the ED	Integrated emergency posts - ED and GP care
Kravet 2008	Cross-sectional study	Patients attending physicians practicing primary care within metropolitan statistical areas	high primary care proportion
Lee 2017	Cross-sectional study	Diabetic adults	Accessibility models to a regular doctor
Lippi Bruni 2016	Cohort study	Patients seeking care who lived in the Emilia-Romagna region	GP clinics extended hours
Lukes 2019	Before-and-after study	Pediatric patients who: were receiving chemotherapy or other immunosuppressive drugs; had received a bone marrow transplant in the previous 100 days; and had experienced known bone marrow failure syndrome, aplastic anemia, or neutropenia and arriving to the ED with report of a temperature measurement of 38.0C (regardless of thermometer type used at home.	Triage nurse given expanded role
Love 2012	Interrupted time series	Patients presenting to the ED	NP in triage program

For peer review only

Lowe 2009	Retrospective cohort	Patients enrolled in the OHP (Oregon Health Authority) at any time between July 1, 2003 and December 31, 2004	Increased primary care capacity
MacKinney 2013	Non-randomized clinical trial	Uninsured patients	Free primary care for uninsured
Morin 2018	Prospective cohort	All patients attending ED	ED patients referred to an out of hours general practice next to ED
Nguyen 2013	Prospective cohort	Low acuity adult patients visiting the ED	Low acuity patients given a same day primary care clinic appointment
O'Keeffe 2008	Pre-post study	Attenders of out-of-hours GP co-op catchment area	GP co-ops to provide out-of-hours primary care
Patel 2012	Pre-post study	Patients aged ≥ 16 years and presenting to the ED with isolated, traumatic, foot, ankle, or knee injuries	ED nurses given expanded role
Philips 2010	Pre-post study	Patients presenting to a GP	GP co-ops to provide out-of-hours primary care
Pierce 2016	Prospective cohort	Patient attending 2 EDs	Provider in triage model
Prescott 2017	Prospective cohort	Patients presenting to the ED	Advanced NPs(ANPs)-led rapid assessment triage
Rust 2009	Retrospective cohort	All patients 18-64 years old who attended EDs in Georgia's rural counties	Community Health Centers in rural counties in Georgia

For peer review only

Rutten 2018	Cohort study	Patients referred for conventional radiology for trauma by one of five GPCs	Unlimited radiology access to GP co-ops
Sharma 2011	Cross-sectional study	Patients presenting to an ED in the province of Victoria	Co-located GP clinics
Sledge 2006	Randomized controlled trial	Patients with ≥ 2 hospital admissions per year in the previous 12-18 months	Primary Intensive Care clinic-based case management intervention
Smith 2018	Retrospective cohort	Children presenting to the ED who were deemed suitable to be treated by a GP	Patients triaged to be seen by a GP between 14:00 and 22:00 hours
Steiner 2009	Prospective cohort	Adults visiting ED	Adding NP to ED
Sterner 2012	Cross-sectional study	Pediatric patients	After-Hours Clinic
Sung 2018	Cross-sectional study	Adults with hypertension	Primary care physician as a usual source of care
Thijssen 2016	Cohort study	First 1000 patients presenting to an ED per hospital	Emergency Care Access Point's
Thijssen 2013	Pre-post study	Patients presenting to the ED	Emergency Care Access Point's
Thrasher 2008	Cross-sectional study	Patients presenting to the ED	Adding NP to ED
Traub 2015	Pre-post study	Patients presenting to the ED	Rapid Medical Assessment team
Tsai 2012	Pre-post study	Children presenting to the ED	Rapid Medical Assessment team
Tucker 2015	Pre-post study	Patients presenting to the ED	NP in fast-track area

For peer review only

Uthman 2018	Cohort study	Non-urgent attendances to the A&E department	GP care in A&E department
van Gils-van Rooij 2018	Cross-sectional study	patients with asthma/ chronic obstructive pulmonary disease (COPD) or a sprained ankle presenting to the ED and/or GP	Urgent Care Collaborations
Van Uden 2006	Cross-sectional study	All patients attending the integrated out of-hours care facility without referral are first seen by a GP, who refers, if necessary, the patient to the ED.	Integrated GP cooperatives
Van Veelen 2016	Pre-post study	Patients presenting to the ED	Integrated GP cooperatives
Wand 2016	Mixed-methods study	Interviews with MHLN team members, patients and ED and psychiatry staff.	NP-led extended hours mental health liaison nurse service
Wang 2014	Pre-post study	Patients with an ESI score of ≥4 (no immediate life-saving intervention and no or only one resource needed, e.g. laboratory test)	Hospital-integrated general practice
Wilson 2008	Cohort study	Adult patients with minor injuries who were triaged at levels 3, 4 and 5	Nurse practitioner in the ED
Wright 1992	Cohort study	patients with quickly diagnosed and treatable illnesses and injuries.	Fast Track system
Zager 2018	Cohort study	Patients presenting to ED	Same-day appointment at the adjoining primary care clinic

For peer review only

Fanta 2006	Prospective cohort	Child between the ages of 2 months and 17 years admitted to the trauma service	care provided by a pediatric trauma nurse practitioner
Lee 2016	Randomized controlled trial	Patients presenting to the ED	Triage nurses order radiographs
Murphy 1996	Randomized controlled trial	Non-emergency patients	care by GP in A&E department
Sinclair 2006	Pre-post study	Mental health patients	Nurse in ED with expanded role
Subash 2004	Non-randomized clinical trial	Patients presenting to the ED	Nurse in ED with expanded role
Ankeny 2014	Cross-sectional study	Patients who had at least one outpatient clinic appointment were included	Patient with PCP appointment
Arain 2013	Pre-post study	Patients attending a walk-in service	GP-led walk-in centres
Badgett 1986	Interrupted time series	Aid to Families with Dependent Children (AFDC) recipients in a metropolitan county	PCP as gate keeper for ED utilization
Barr 2000	Cross-sectional study	Low acuity patients	NP - interpret x ray, performance
Baughman 2016	Retrospective cohort	Patients with depression	Patient-centered medical home
Beales 1997	Pre-post study	Unclear (assume it is ED patients)	Primary Care / Minor Injuries Unit
Beales 1995	Pre-post study	Unclear (assume it is patients attending a minor injuries unit)	Minor Injuries Unit

For peer review only

Begaz 2015	Randomized controlled trial	Non-pregnant adults with a chief complaint of abdominal pain	Triage nurse order x-ray and diagnostics
Bitton 2009	Pre-post study	Adult patients attending a clinic	Patient-centered medical home
Bleijenberg 2016	Randomized controlled trial	Frail individuals aged 60 and older	NP care
Bonham 1987	Pre-post study	AFDC-related Medicaid recipients	PCP as gate keeper for ED utilization
Breslaua 2018	Prospective cohort	Age 18 through 64, who were continuously enrolled in Medicaid, and received treatment in a study clinic (either PBHCI or control), during both the baseline and intervention periods.	Integrated GP care
Bynum 2011	Cohort study	Elderly residents of four continuing care retirement communities.	After-hours GP or NP are
Byrne 2000	Cohort study	The sample selected mainly presented with simple lacerations, soft tissue injuries, fractures, and dislocations and was, therefore, typical of adult patients attending the units with a minor injury and consistent with national patterns	Minor Injuries Unit
Campbell 2012	Cross-sectional study	Patients who presented to the ED	NP care in ED

For peer review only

Carter 2016	Prospective cohort	Diabetic patient population	Family Medicine Groups
Casalino 2017	Cohort study	Patients visiting ED	High primary care availability
Cecil 2016	Cross-sectional study	Children aged <15 years	Increased primary care access
Cloutier 2005	Pre-post study	Children with asthma who presented for medical care regardless of payer or chief complaint at any of the 6 primary care clinics in Hartford between 1998 to 2002	PCP asthma management program
Conway 2004	Cohort study	Patients who were admitted to the medical assessment unit (MAU) with a provisional diagnosis of respiratory disease	NP care in ED
Cooper 2002	Randomized controlled trial	Patients aged 16 and over presenting to the ED with minor injuries	NP care in ED
Covington 1992	Pre-post study	Low acuity patients	NP fast track
Cuellar 2016	Prospective cohort	All adults aged 18 to 64 years who were covered by CareFirst for at least 3 consecutive months between 2010 and 2013	Patient-centered medical home
Dorado 2015	Non-randomized clinical trial	Asthmatic patients	GP asthma care
Driscoll 2013	Interrupted time series	Alaska Native and American Indian people who live in the Anchorage area and receive<br>primary care services from SCF and those	Patient-centered medical home

For peer review only

		who live<br>in other areas of the state who may be transported to<br>ANMC for tertiary care	
Eichler 2011	Pre-post study	All consecutive ambulatory emergency patients	GP-led emergency service
Farion 2010	Pre-post study	Low acuity patients	Ambulatory zone for low acuity patients
Fleetcroft 2016	Cross-sectional study	patients with asthma	Increased primary care access
Gabayan 2009	Cross-sectional study	Patients living in California	having a usual source of care
Gardner 2017	Non-randomized clinical trial	Adult patients who did not require consultation or admission	NP triage
Gedmintas 2012	Pre-post study	Patients presenting to the ED	NP rapid assessment zones
Grasso 2015	Pre-post study	All patients who presented for emergency medical care	Follow-up appointments in a GP clinic
Green 2018	Prospective cohort	Adults	Patient-centered medical home
Hansagi 1989	Cohort study	Adult patients who attended the ED with non-urgent ailments	Low acuity ED patients referred to primary care
Harris 2016	Retrospective cohort	All patient records of veterans 51 years or older with<br>an International Classification of Diseases,	Shared medical appointments

For peer review only

		Ninth Revision<br>(ICD-9) code of 250.00 for type 2 diabetes<br>were<br>included.	
Hebert 2013	Interrupted time series	Study sample included 8.5 million patients assigned to a primary care provider at any time from January 2003 through October 2012 at 972 clinics.	Patient-centered medical home
Hunold 2014	Cross-sectional study	Individuals aged 65 and older across the state of North Carolina	High GP presence
Hwang 2012	Retrospective cohort	Uninsured patients	Free GP clinic
Ingram 2013	Pre-post study	Ambulatory patients who present with chest pain	Nurse with expanded role
Jamshed 2018	Prospective cohort	Age 65 and over, minimum 2 chronic conditions and homebound status	Home Based Primary Care Program
Keogh 2015	Cross-sectional study	Homeless people in Dublin (Safety net)	Free to access primary care service
Kim 2017	Cross-sectional study	Adults aged 18 years or older	Increased access to GP
Klemmer 2018	Cohort study		NP led Clinic
Krakau 1992	Prospective cohort	Patients who contacted ambulatory medical care services	Increase in number of GP posts

For peer review only

Krook 2013	Pre-post study	Patients who present to the ED but have no family doctor as a routine primary care provider	Follow-up for unattached patients
Krupski 2016	Prospective cohort	Clients who were receiving mental health services and were enrolled in Primary and Behavioural Health Care Integration (PBHCI)	Integrating Primary Care into Mental Health Centers
Laine 2005	Retrospective cohort	Drug users enrolled in the NYS Medicaid program	Onsite medical care
Lee 2014	Prospective cohort	Patients attending the ED	Primary care based accountable care organizations
Liferidge 2015	Pre-post study	Patients attending the ED	On-site primary care clinic
Long 1998	Pre-post study	Patients presenting to the ED who could be treated in other settings	Coordinated and cost-effective community-based primary care
Lynch 2014	Pre-post study	Patients with >2 ED visits or hospital admissions in the past 6 months, ≥3 chronic illnesses, and psychosocial complexity	Primary care clinic
Mackenzie 2013	Pre-post study	All ambulatory discharged patients were included	Rapid Assessment Unit
Manns 2012	Cohort study	Diabetic patients	Primary care networks
Martin 2013	Cross-sectional study	All patients triaged to the fast track (FT) area of the ED	NP-led fast track

For peer review only

McClellan 2011	Randomized controlled trial	Adult patients with acute peripheral musculoskeletal soft tissue injuries presenting to EDs	NP care
McDonnell 2015	Retrospective cohort	Children presenting to a pediatrics ED	NP care
Megahy 2004	Cross-sectional study	All patients over the age of 16 who attended A&E over one month	NP care
Mitchell 2005	Randomized controlled trial	Asthmatic children	GP education intervention
Muller 2018	Retrospective cohort	Children who presented to a pediatric emergency department	Nurse practitioner in the ED
Munday 2012	Pre-post study	Patients attending Robina hospital	Medical Assessment Unit
Musich 2016	Pre-post study	Between the ages of 35 to 84 years and having continuous health plan membership	MD-Value in Prevention - personalized preventive health care
Nelson 2016	Cohort study	Veterans who received primary care	Patient-Centered Medical Home
Newman 2017	Interrupted time series	Patients who attended a PCMH family medicine residency practice	Patient-Centered Medical Home
Nyweide 2017	Prospective cohort	Continuously enrolled fee-for-service Medicare beneficiaries 66 years of age or older by the end of 2011 from a 20% random sample were identified in the Master Beneficiary	Higher continuity of care

For peer review only

		Summary<br>File in the Chronic Conditions Warehouse	
O'Kelly 2010	Interrupted time series	Patients in the lower acuity triage categories 4 and 5	Access to an out of hour GP
Olsen 2016	Pre-post study	Patient with chronic pain and one of more of the following: frequent ED visits or deceitful behavior about their prescription history.	Chronic pain protocol involving patient's treating physician
Organ 2005	Pre-post study	Children presenting with minor injuries and illnesses	NP care in the ED
O'Toole 2010	Retrospective cohort	Homeless veterans	Population-tailored primary care clinic
Owens 2012	Cohort study	Patients with a life-limiting illness	Primary Palliative Care Clinic
Parsons 1997	Pre-post study	Chronically ill elderly patients	interdisciplinary primary care geriatric practice
Peterson 2017	Pre-post study	Patients attending the CareFirst program	Extending CareFirst's Medical Home Program
Philips 2011	Interrupted time series	Patient's age 65 years or older	WellMed - Coordinated primary care
Pickin 2004	Pre-post study	Patients attending GPs	GP co-operative
Pirraglia 2012	Prospective cohort	All veterans enrolled in SMIPCC	Co-Located Primary care clinic

For peer review only

Raffetto 2014	Prospective cohort	Patients enrolled in a medical home pilot	patient-centered medical home
Raphael 2015	Cross-sectional study	Low-income children with chronic conditions	patient-centered medical home
Rautava 2013	Pre-post study	Patients visiting health services	Out-of hours primary healthcare services
Rhee 1995	Case-control study	Patients visiting ED	NP-led care in ED
Rivara 1986	Prospective cohort	Pediatric ED patients	General appointment clinic
Riverin 2017	Retrospective cohort	"Vulnerable patients" - patients ≥ 70 years of age, or with one or more specified chronic health conditions, including psychosis, COPD, moderate to severe asthma, pneumonia, cardiovascular disease, cancer associated with past, present, or future chemotherapy or radiotherapy treatments, cancer in terminal phase, diabetes, alcohol or drug withdrawal, drug addiction treated with methadone, HIV/AIDS, or a degenerative disease of the nervous system.	Family Medicine Groups
Rogers 2004	Pre-post study	Anyone who presented with a minor illness or injury and who could be assessed and treated within 15 min was thought to be suitable for inclusion into a S&T system.	See and Treat system

For peer review only

Rosenthal 2016	Pre-post study	Patients age less than 65 years old	patient-centered medical home
Rosenthal 2013	Interrupted time series	Patients attending 5 primary care practices	patient-centered medical home
Rothkopf 2011	Prospective cohort	Colorado Medicaid clients who had two or more office or clinic visits during the state's fiscal year 2008 (July 1, 2007–June 30, 2008), who were not enrolled in a managed care organization during the year, and whose usual source of care was either a community health center or a private fee-for-service provider	Community health center
Savage 2013	Pre-post study	Patients attending clinics	patient-centered medical home
Schwartzman 2001	Interrupted time series	Patients with respiratory disease requiring urgent acute treatment or urgent investigation. Patients seen in our outpatient clinic or emergency department who are expected to require 2 or more hours of care. Patients may also be transferred to our day hospital from inpatient wards, from other emergency departments and hospital clinics and from physicians' private offices.	Respiratory day care
Seaberg 2010	Prospective cohort	Low acuity pediatric patients	Redirecting low-acuity patients to GP

For peer review only

Shea 2012	Pre-post study	Patients presenting to ED	Patient-centered NP rapid team triage
Sheikh 2016	Prospective cohort	Children with asthma referred to the Pediatric Asthma Center at local Children's hospital between 2011-2014	NP-led asthma centre
Shumacher 2014	Prospective cohort	Optum Care Plus long-term care patients and Medicare 5% patients	GP on-site care to nursing homes residents
Singer 2017	Cross-sectional study	All residents > 14 years old.	GP-led medical home practices
Sjonell 1986	Pre-post study	patients seeking medical care in specific districts in Stockholm, Sweden	Primary Health Care Centre
Smith 2015	Interrupted time series	Patients with DM type 2 with at least one visit to the ANMC campus in the previous 3 years	Patient centered medical home models
Smith-Campbell 2005	Pre-post study	Uninsured clients	Community Health Center
Sprandio 2010	Prospective cohort	Cancer patients	Oncology patient centered medical home
Stoddart 1999	Pre-post study	patients attending the ED	Out-of hours primary care emergency centres
Stone 2013	Prospective cohort	Low-acuity patients	Medical screening exam and a timely referral
Strange 1994	Pre-post study	Patients in the ED with minor health care problems	NP care in ED

For peer review only

Sukpraprut-Braaten 2016	Retrospective cohort	Patients presenting to the ED. Children under 8 years old, adults older than 65 years old, diabetic patients, cancer patients, patients with fever higher than 102 F, laceration, skin abscess, and recent injury are not eligible for MSE.	Redirection of non-urgent patients
Terp 2013	Retrospective cohort	Patients ≥ 18 years old visiting non-federal emergency departments in California	GP density
Timble 2017	Mixed-methods study	Patients (beneficiaries) attending demonstration sites and comparison sites	Transformation of primary care practices in medical homes
To 2015	Cross-sectional study	Individuals living with asthma in Ontario, Canada	practice model of primary care provider (blended fee-for-service, blended capitation)
Tsai 2009	Pre-post study	Uncomplicated pediatric patients visiting ED	NP-led rapid medical assessment
van der Linden 2010	Retrospective cohort	All patients identified as "low-care" by the triage	Emergency nurse practitioner at emergency department
van Uden 2003	Cohort study	Patients attending a GP cooperative in two Dutch cities	The Maastricht GP cooperative
Watts 2016	Pre-post study	Veterans in the top 10% of overall 3-months risk for morbidity or mortality (according to the Care Assessment Needs score) and at least one acute care visit in the last 6 months.	Patient-centered medical home

For peer review only

Weinberger 1996	Randomized controlled trial	Patients hospitalized in the General Medicine Service were potentially eligible if they had a diagnosis of diabetes mellitus, chronic obstructive pulmonary disease, or congestive heart failure that was documented in the medical record at or before the time of the index admission (and that was not necessarily the reason for that admission)	Intensive primary care intervention
Wright 2010	Cross-sectional study	Patients under care of family physicians in each area	Increased GP in area
Xin 2014	Retrospective cohort	Insured individuals aged 18 and older. Two cohorts were selected, 2007-2008 and 2009-2010, which are nonoverlapping sequential samples.	Patient-centered medical home
Celona 2018	Pre-post study	Fast track ED adult patients	NP in ED
Cheung 2002	Cohort study	ED patients - a random sample of 250 patients was selected	Triage nurse initiated diagnostic protocols
Considine 2006	Case-control study	The adult presentations eligible for ENPC management were: laceration and wounds, forearm/wrist injury, lower leg/ankle injury, hand injury, symptoms suggestive of cellulitis, foot injury, knee injury, calf pain suggestive of deep vein thrombosis, elbow injury, plaster of Paris (POP) complication, vomiting in pregnancy (hyperemesis), symptoms suggestive of urinary tract	NP in ED

For peer review only

		infection/pyelonephritis, vomiting and diarrhoea, minor burns, breast pain and inflammation suggestive of mastitis, loin pain suggestive of renal colic and viral symptoms suggestive of upper respiratory tract infection.	
Fry 2009	Prospective cohort	Ten percent of ED patients that were assigned to be managed by the Emergency Transitional Nurse Practitioner (ETNP)	NP in ED
Fry 2011	Prospective cohort	Patients presenting with minor injuries and illnesses to one metropolitan emergency department (ED).	NP in ED
Gray 2013	Pre-post study	Children attending the Caboolture Hospital ED	Pediatric flow nurse in ED
Jennings 2015	Randomized controlled trial	All patients > 16 years of age, presenting to the emergency department with a complaint of "pain" and allocated for their episode of care to the "fast-track" zone were eligible for inclusion in the trial.	NP-led fast track
Lee 2006	Prospective cohort	Patients who presented to the ED and either did not wait for service or were seen by a nurse practitioner.	NP in ED
Chu 2017	Retrospective cohort	Medicaid beneficiaries who qualify for services on the basis of having a disability	Patient-centered medical home
Lutzke 2011	Retrospective cohort	TENP patients	NP-led see and treat stream

For peer review only

MacKenzie 2015	Pre-post study	Included were all ambulatory discharged patients	Rapid Assessment Unit
Nash 2007	Retrospective cohort	All patients visiting FT area (Sept 2004-Feb 2005) and patients visiting MC area (Sep 2003-Feb 2004)	NP-led fast track
Rothwell 2018	Pre-post study	Participants included adults and children who presented to the ED with minor trauma	NP-led fast track
Salisbury 2007	Pre-post study	Patients consulting in the ED facilities	Walk-in centres co-located with ED
Thompson 2012	Retrospective cohort	Patients at least 3 years old, presented with a non-complex injury or illness that met the ANP's scope of practice	NP in ED
Wand 2015	Mixed-methods study	Patients with mental health problems who received direct clinical care from the ED-based mental health liaison nurse (MHLN). The types of mental health-related presentations varied and were not related to psychiatric diagnoses.	NP-led extended-hours nurse team
Shrimpling 2002	Before-and-after	All patients attending A&E department at Kettering General Hospital with minor injuries	NP-led minor injuries team
Dale 1996	Non-randomized controlled trial	All patients presenting with primary care problems at King's College Hospital accident and emergency triage system and being treated in the sessions selected for the study	GP consultations for low-acuity patients

For peer review only

Copeland 2015	Before-and-after study	Patients of all CTAS levels triaged at Strathroy Middlesex General Hospital emergency department	Separate low-acuity patient stream
Day 2013	Before-and-after study	Patients presenting to the ED	Redirection of non-urgent patients
Lindley-Jones 2000	Randomized controlled trial	All patients who presented to the department during the study period and who were eligible for a nurse requested x ray according to our departmental guidelines	Triage nurse requesting X-ray
Parris 1997	Quasi-randomized controlled trial	Patients aged 14 years or more with isolated limb injuries	Triage nurse requesting X-ray
Sanchez 2006	Before-and-after study	Patients attending the emergency department of the hospital	NP-led fast track
Thurston 1996	Simultaneous prospective trial	Patients older than 5 years presenting with recent peripheral limb injuries.	Nurse given increased authority
Winston 1981	Before-and-after study	Patients with less urgent problems. A random sample of patients was used to find the proportion of total patient time that is spent waiting. The waits and entire visits of patients were to be timed and the hypothesis was that the ratio of waiting time to total care time would be the same before and after the program had begun.	NP program for low-acuity patients
Begum 2016	Prospective cohort study	150 patients over 5 weeks (June 9 to July 10)	GP in triage treat and re-direct low-acuity patients

For peer review only

Jennings 2008	Retrospective cohort	Any patient visiting an adult level one trauma center with an ATS categories 3–5	NP in ED
Bodenmann 2015	Randomized controlled trial	Adult frequent emergency department users (5 or more visits during the previous 12 months)	Interdisciplinary case-management
Chu 2016	Pre-post study	Non seniors and people with disabilities (SPD) population younger than 65 years old	Patient-centered medical home
Miller 2019	Interrupted time series	Socioeconomically deprived population attending a general practice	GP telephone-first consultation
Wallis 2018	Before-and-after study	Adults aged 70 years and older	NP geriatric service
Lee 1996	Cohort study	Patients aged 3 years and older, with blunt injury to one region only of a limb were included. Patients who were critical, younger than 3 years, with multiple limb injuries, or with hip or pelvis injuries were excluded.	Triage nurse-initiated X-ray
Klassen 1993	Randomized controlled trial	Children less than 18 years of age who had a history of extremity trauma in the preceding seven days	Triage nurse-initiated X-ray
Al Kadhi 2017	Retrospective chart review	Patients fulfilling criteria for fast track (no evidence of hypovolemia or shock, no positive pregnancy test, no solitary kidney, or kidney transplant and so on (Figure 1)	Triage nurses using the Renal Colic Fast Track protocol

For peer review only

Ashurst 2014	Prospective study	Patients aged 16 years or older, spoke English or Spanish, and sustained an isolated traumatic ankle or foot injury with no other complaints or injuries present.	Triage nurse-initiated radiographs
Retezar 2011	Retrospective nested cohort study	All adult patients who presented with a chief complaint for which triage standing orders had been developed and waited longer than 15 minutes after triage assessment for room placement were eligible for the study	Triage nurse-initiated radiographs
Boeke 2010	Before-and-after study	Patients 16 years of age or older, attending the A&E department on weekdays from 10 to 17 hours	GP in the ED
EDM 2008	Before-and-after study	Patients attending ED at Jefferson Memorial Hospital in Ranson, WV	NP-led mobile unit outside ED
Fan 2006	Randomized controlled trial	Patients 18 years or older with ankle or foot twisting injuries, occurred within seven days.	Triage nurse-initiated radiographs
Heaney 1997	Before-and-after study	Patients presenting minor injuries	NP-led minor injuries clinic
Derlet 1992	Retrospective cohort study	Patients aged 15 years or older, without the following conditions: severe pain, chest or abdominal pain, arrival by ambulance, inability to walk and vital signs outside established ranges	Triage nurse given expanded role

For peer review only

Moe 2019	Interrupted case series	Population of West View Health Centre (Stony Plain, Alberta) catchment area	Primary care after-hours clinic
Schmidt 2019	Retrospective cohort study	Patients enrolled in the Center for Autism Services and Transition (CAST), with ≥ 1 ED visit	Primary care center for adults with autism
Martin 2019	Before-and-after study	High need high-cost patients	Team-based primary care and care coordination
McCarron 2019	Retrospective chart review	Pediatric patients	Co-located GP
James 2019	Retrospective case note analysis	Children and young people	Primary Care Hub
Thompson 2019	Before-and-after study	Children and young people	Children's and Young People's Ambulatory Unit
Michael 2019	Retrospective chart review	All refugees who established medical care in the two primary health systems in our county (n=285) were included.	Refugee Health Collaborative - Patient-centered medical home
Sikkenga 2016	Retrospective quasi-experimental study	Patients ≥ 15 years of age who received screening for CT (Chlamydia trachomatis)/NG (Neisseria ghonorrhoeae) in the ED	Triage nurse initiated rapid testing
Dewhirst 2015	Retrospective chart review	Patients attending ED	Nurse-initiated analgesia protocol

For peer review only

Hackman 2015	Retrospective chart review	Chest pain patients over 29 years old with a troponin ordered.	Nurse-Initiated Chest Pain Protocol
Dixon 2014	Cluster randomized controlled trial	Children aged 6 years and younger who presented to the emergency department with a presentation consistent with radial-head subluxation and who had sustained a known injury in the previous 12 hours.	Nurse-initiated treatment
Lee 2014	Randomized controlled trial	Adult patients presenting within 10 days of isolated blunt ankle trauma	Triage nurse-initiated radiographs
Adam 2014	Randomized controlled trial	Patients aged 6 months to 12 years, with acute isolated extremity injuries	Triage nurse-initiated radiographs
Fontanel 2011	Experimental trial	Patients with any isolated limb trauma injury less than 24 h old, without signs of severe illness.	Triage nurse-initiated radiographs
Gaucher 2010	Retrospective cohort study (chart review)	All triaged children who LWBS	Triage Nurse Counselling
Demarco 2010	Prospective cohort study with historical and concurrent controls	Adult patients presenting to the ED	Triage nurse initiated rapid testing
Jobé 2013	Randomized controlled trial	Patients with suspected acute coronary syndrome	Triage nurse with expanded role
Douma 2016	Randomized controlled pragmatic trial	Patients who met protocol inclusion criteria, which was specific to each protocol.	Triage nurse-initiated protocols

For peer review only

Lijuan 2017	Before-and-after study	Patients presenting to the ED scoring ESI levels 3,4 or 5	Triage nurse-initiated order sets
Almeida 2019	Retrospective cohort study	All adult patients who presented to the ED	Family Health Units
Al Abri 2020	Quasi-experimental design	Age 18 years and above; seeking health care in the ED within 12 h from the time of injury	Triage nurse-initiated radiographs
Ho 2018	Randomized controlled trial	Aged one year old and above, suffered from blunt ankle injuries for not more than 10 days	Triage nurse-initiated radiographs
Li 2018	Retrospective cohort study	Pediatric patients who had no life-threatening condition and did not arrive to the ED by ambulance	Triage nurse-initiated tests
Wright 2018	Retrospective cohort study	Medicare beneficiaries in underserved rural areas	Rural Health Clinics
Kobewka 2019	Retrospective cohort study	Patients older than 65 years living in the survey-respondent LTC homes between Jan 1 and May 30, 2017	Same-day physician access
Chakravorty 2019	Retrospective, Before-and-after study	Children with asthma, enrolled in Florida's Medicaid or Children's Health Insurance Program (CHIP)	Pediatric primary care practices
Ray 2019	Before-and-after study	All presentations made to the ED	Fast-track

For peer review only

Johnson 2019	Before-and-after study	Patients with at least 3 inpatient admissions in the prior 12 months were eligible	Coordinated Care Center (primary care clinic)

GP: General practitioner**; NP:** Nurse practitioner; **ED**: Emergency department

For peer review only

Appendix Table 8: Emergency department (ED) outcomes reported by the included studies.

Author, Year	A	B	C	D	E	F	G	H	I	J	K	L	M	N	O	P	Q	R	S	T	U	V	W	X	Y	Z	AA	AB
Anantharaman 2008	0										0																	
Lowe 2006	+																											
Carter 2014			+																									
Chmiel 2016											+																	
Clancy 2009											+																	
Colligan 2011	0		+	+			+		0																			
Considine 2008		+	+																									
Dahrouge 2016	+																											
Jones 2011		+	+																									
Dias 2016					0																							
Dinh 2012		0			+							0																
Dolton 2016	+				-																							
Ducharme 2009		+	+	+																								
Edwards 2011		+	+																									
Eichler 2014												+																
Gardner 2018		+	+	+																								
Hansagi 1990	+				0						+																	
Hayden 2014			0	0	0		+																					
Hearld 2018	+																											
Hearld 2012	+																											
Hearld 2017	+																											
Jeanmonod 2013					0																	+				+		
Kim 2017	0																											
Kool 2008	+	+			+																							

For peer review only

Kravet 2008	+																											
Lee 2017	+																											
Lippi Bruni 2016	+													+														
Love 2012				+			+																					
Lowe 2009	+																											
MacKinney 2013	+										0																	
Morin 2018					+					+	+																	
Nguyen 2013	+				+																							
O'Keeffe 2008	0																											
Patel 2012			0				_																					
Philips 2010	0																											
Pierce 2016			+																									
Prescott 2017		+																										
Rust 2009	+																											
Rutten 2018	+																											
Sharma 2011		+																										
Sledge 2006	0																0											
Smith 2018			+	+																								
Steiner 2009		0	0	0																								
Sterner 2012			+									+																
Sung 2018	+																											
Thijssen 2016			0																									
Thijssen 2013	+																											
Thrasher 2008					+																							
Traub 2015			+	0																								
Tsai 2012	-		+	+			+																					
Tucker 2015	0		+	+	0		+								+	_												
Uthman 2018	0		+	+																								

For peer review only

van Gils-van Rooij 2018	-	-																										
Van Uden 2006																	0											
Van Veelen 2016	+		-								+																	
Wand 2016			+	+	+																							
Wang 2014			+				0		+									+	+									
Wilson 2008		+								+																		
Wright 1992				+	+																							
Zager 2018	+						+	+																				
Fanta 2006			+		0							0																
Lee 2016			+		0																							
Murphy 1996	+				0			+						+														
Sinclair 2006	0	0			0																							
Subash 2004		+	+				+																					
Ankeny 2014	+																											
Arain 2013	0				0																							
Badgett 1986	+																											
Barr 2000		+			+																							
Baughman 2016	0																											
Beales 1997		+																										
Beales 1995		+																										
Begaz 2015			0						+												0							
Bitton 2009	+											+					0											
Bleijenberg 2016	0				0																							
Bonham 1987	+				+					+																		
Breslaua 2018	+																											
Bynum 2011	+																											

Byrne 2000		+	+		+																		_	_				
Campbell 2012		+		0																								
Carter 2016	+																											
Casalino 2017	+																											
Cecil 2016	+																											
Cloutier 2005	+																											
Conway 2004		+			+																							
Cooper 2002	0	+			+																							
Covington 1992				+	+																							
Cuellar 2016	+													+														
Dorado 2015	+																											
Driscoll 2013	+																											
Eichler 2011		+			0			_					0															
Farion 2010			+	+	+		+																					
Fleetcroft 2016	+																											
Gabayan 2009	0																											
Gardner 2017			+	+	+		+																					
Gedmintas 2012		0		+																								
Grasso 2015	+																											
Green 2018	+											+																
Hansagi 1989	+																											
Harris 2016	0																											
Hebert 2013	0																											
Hunold 2014	0																											
Hwang 2012	0																											
Ingram 2013			+	+							+																	
Jamshed 2018	+																											

For peer review only

Keogh 2015	0																											
Kim 2017	+																+											
Klemmer 2018		+		+	+																							
Krakau 1992	+																											
Krook 2013	+				+									+														
Krupski 2016	0																											
Laine 2005	+																											
Lee 2014	+		+																									
Liferidge 2015	-																											
Long 1998	0																											
Lynch 2014	+																											
Mackenzie 2013		+	+				+																		+	–		
Manns 2012	+																											
Martin 2013			+		+		+		+																			
McClellan 2011																	0											
McDonnell 2015																						0						
Megahy 2004					+																							
Mitchell 2005	0																											
Muller 2018			+	+																								
Munday 2012			+																									
Musich 2016	+																											
Nelson 2016	+																											
Newman 2017	+																											
Nyweide 2017	+																											
O'Kelly 2010	+							+																				
Olsen 2016	+																											

For peer review only

Organ 2005				+	+																							
O'Toole 2010	-																			0								
Owens 2012	+																											
Parsons 1997	+																											
Peterson 2017	0																											
Philips 2011	0																											
Pickin 2004	0				+																							
Pirraglia 2012	0																											
Raffetto 2014	0																											
Raphael 2015	+																											
Rautava 2013								+																				
Reich 2018	-																											
Rhee 1995					0																							
Rivara 1986											+																	
Riverin 2017	+																											
Rogers 2004			+				+																					
Rosenthal 2016	+													+														
Rosenthal 2013	+																											
Rothkopf 2011	+																											
Savage 2013	+				+																							
Schwartzman 2001	+				0																							
Seaberg 2010					+						+																	
Shea 2012			+	+	0																							
Sheikh 2016	+																											
Shumacher 2014	+																											
Sjonell 1986	+																											

For peer review only

Smith 2015	+																											
Smith-Campbell 2005	+													+														
Sprandio 2010	+																											
Stoddart 1999	+							0																				
Stone 2013	+				+						+																	
Strange 1994		+			+																							
Sukpraprut-Braaten 2016		+																										
Terp 2013	+																											
Timble 2017	-																											
To 2015	+																											
Tsai 2009			+	+			+																					
van der Linden 2010		0	+																									
van Uden 2003	0																											
Watts 2016	+																											
Weinberger 1996	0				+																							
Wright 2010	+																											
Xin 2014	0																0											
Celona 2018	-			+			+		+																			
Cheung 2002			+						+																			
Considine 2006		0	0						0																			
Fry 2009			+				+																					
Fry 2011	+	+	+	+																								
Gray 2013			+		+																							
Jennings 2015		0	0	0																								
Lee 2006		+																										

For peer review only

Chu 2017	+																											
Lutzke 2011										+																		
MacKenzie 2015			+	+			+		–																			
Nash 2007	+			+	+																							
Rothwell 2018				+	+		+																					
Salisbury 2007	0		0									0																
Thompson 2012		+																										
Wand 2015					+		+				+																	
Shrimpling 2002						+																						
Dale 1996					0																							
Copeland 2015		+	+	0		+																						
Day 2013	0		+																									
Lindley-Jones 2000		0																										
Parris 1997			0																									
Sanchez 2006	-	+	+	+																								
Thurston 1996		0	+																									
Winston 1981	-		0													+												
Begum 2016					+																							
Bottle 2018	0										0																	
Buerhaus 2018	+																											
Chang 2018	+																											
Crits-Cristoph 2018	0																											
Kao 2019	+																											

For peer review only

Kern 2018	+																											
Kern 2019	+																											
Kuo 2018	+																											
Lagisetty 2018	+																											
Lindly 2018	0										0																	
Lukes 2019																											+	
McAlister 2018	+																											
Mittmann 2018	0																											
Nichols 2018	0																											
Platter 2019	0										0																	
Preston-Suni 2019		+		+			+																					
Ravichadran 2019	+		+																									
Tsai 2018	+																											
Tucker 2018	+																											
van der Bersselaar 2018	+																											
Vinton 2019			+	0																								
Voelker 2018	+																											
Wu 2018	+																											
Yuan 2018	+																											
Almeida 2019	+																											
Al Abri 2020			+				+																					
Miller 2019	0																											
Wallis 2018	0		+											+														
Ho 2018			+				0																			+		
Li 2018			+	-																								

For peer review only

Lee 1996			+				_																					
Klassen 1993			+																									
Al Kadhi 2017			+																									
Ashurst 2014			+		+																							
Boeke 2010			+		+																							
EDM 2008				+																								
Fan 2006			+	+	0																					+		
Heaney 1997	+																											
Derlet 1992											+										+							
Moe 2019	+													+														
Wright 2018	-																											+
Schmidt 2019	+																											
Kobewka 2019	+																											
Johnson 2019	+																											
Martin 2019	+																											
McCarron 2019	+		+																									
James 2019			+																									
Thompson 2019			+																									
Michael 2019	+																											
Chakravorty 2019	+																											
Sikkenga 2016			0																									
Dewhirst 2015			0																									
Hackman 2015			0																									
Dixon 2014			+																									

For peer review only

Lee 2014			0		+																							
Adam 2014			+																								+	
Fontanel 2011			0																									
Demarco 2010			+		+																							
Jobé 2013			0				+																					
Ray 2019							+																					
Douma 2016			+																									
Lijuan 2017			+	0	+																							
Jennings 2008		+	+																									
Retezar 2011																										+		
Gaucher 2010	+																											
Singer 2017	+																											
Bodenmann 2015	+																											
Chu 2016	+																											

A: Emergency department (ED) visits; **B**: ED wait time; **C**: ED Length of stay; **D**: Leave without being seen; **E**: Patient Satisfaction; **F**: Percentage of patients with reduced wait times in ED; **G**: Time to initial physician assessment; **H**: Volume of patients visiting ED; **I**: ED workup time; **J**: Patient safety; **K**: Number of patients diverted to Primary Care; **L**: Treatment costs per patient; **M**: Annual local budget for treatment of ED patients; **N**: Cost saving; **O**: Bed-to-provider time; **P**: Leave against medical advice (AMA); **Q**: Total Cost; **R**: Time from admission to first nursing activity; **S:** Time from admission to communication of the diagnosis to the patients; **T**: Decrease in non-urgent/ low-acuity ED visits; **U**: left before completion of service; **V**: Relative value units (RVUs); **W**: Length of first consultation; **X**: Wait time to receive other services; **Y**: Triage to ED Entry; **Z**: Being seen in the ED to discharge; **AA**: Time to first order; **AB**: Potentially preventable ED visits; **+**: Positive impact; **-**: Negative impact; **0**: No impact.

For peer review only

Preferred Reporting Items for Systematic reviews and Meta-Analyses extension for Scoping Reviews (PRISMA-ScR) Checklist

SECTION	ITEM	PRISMA-ScR CHECKLIST ITEM	REPORTED ON PAGE #
**TITLE**			
Title	1	Identify the report as a scoping review.	
**ABSTRACT**			
Structured summary	2	Provide a structured summary that includes (as applicable): background, objectives, eligibility criteria, sources of evidence, charting methods, results, and conclusions that relate to the review questions and objectives.	
**INTRODUCTION**			
Rationale	3	Describe the rationale for the review in the context of what is already known. Explain why the review questions/objectives lend themselves to a scoping review approach.	
Objectives	4	Provide an explicit statement of the questions and objectives being addressed with reference to their key elements (e.g., population or participants, concepts, and context) or other relevant key elements used to conceptualize the review questions and/or objectives.	
**METHODS**			
Protocol and registration	5	Indicate whether a review protocol exists; state if and where it can be accessed (e.g., a Web address); and if available, provide registration information, including the registration number.	
Eligibility criteria	6	Specify characteristics of the sources of evidence used as eligibility criteria (e.g., years considered, language, and publication status), and provide a rationale.	
Information sources*	7	Describe all information sources in the search (e.g., databases with dates of coverage and contact with authors to identify additional sources), as well as the date the most recent search was executed.	
Search	8	Present the full electronic search strategy for at least 1 database, including any limits used, such that it could be repeated.	
Selection of sources of evidence†	9	State the process for selecting sources of evidence (i.e., screening and eligibility) included in the scoping review.	
Data charting process‡	10	Describe the methods of charting data from the included sources of evidence (e.g., calibrated forms or forms that have been tested by the team before their use, and whether data charting was done independently or in duplicate) and any processes for obtaining and confirming data from investigators.	
Data items	11	List and define all variables for which data were sought and any assumptions and simplifications made.	
Critical appraisal of individual sources of evidence§	12	If done, provide a rationale for conducting a critical appraisal of included sources of evidence; describe the methods used and how this information was used in any data synthesis (if appropriate).	
Synthesis of results	13	Describe the methods of handling and summarizing the data that were charted.	

For peer review only - http://bmjopen.bmj.com/site/about/guidelines.xhtml

SECTION	ITEM	PRISMA-ScR CHECKLIST ITEM	REPORTED ON PAGE #
**RESULTS**			
Selection of sources of evidence	14	Give numbers of sources of evidence screened, assessed for eligibility, and included in the review, with reasons for exclusions at each stage, ideally using a flow diagram.	
Characteristics of sources of evidence	15	For each source of evidence, present characteristics for which data were charted and provide the citations.	
Critical appraisal within sources of evidence	16	If done, present data on critical appraisal of included sources of evidence (see item 12).	
Results of individual sources of evidence	17	For each included source of evidence, present the relevant data that were charted that relate to the review questions and objectives.	
Synthesis of results	18	Summarize and/or present the charting results as they relate to the review questions and objectives.	
**DISCUSSION**			
Summary of evidence	19	Summarize the main results (including an overview of concepts, themes, and types of evidence available), link to the review questions and objectives, and consider the relevance to key groups.	
Limitations	20	Discuss the limitations of the scoping review process.	
Conclusions	21	Provide a general interpretation of the results with respect to the review questions and objectives, as well as potential implications and/or next steps.	
**FUNDING**			
Funding	22	Describe sources of funding for the included sources of evidence, as well as sources of funding for the scoping review. Describe the role of the funders of the scoping review.	

JBI = Joanna Briggs Institute; PRISMA-ScR = Preferred Reporting Items for Systematic reviews and Meta-Analyses extension for Scoping Reviews.

* Where *sources of evidence* (see second footnote) are compiled from, such as bibliographic databases, social media platforms, and Web sites.

† A more inclusive/heterogeneous term used to account for the different types of evidence or data sources (e.g., quantitative and/or qualitative research, expert opinion, and policy documents) that may be eligible in a scoping review as opposed to only studies. This is not to be confused with *information sources* (see first footnote).

‡ The frameworks by Arksey and O'Malley (6) and Levac and colleagues (7) and the JBI guidance (4, 5) refer to the process of data extraction in a scoping review as data charting.

§ The process of systematically examining research evidence to assess its validity, results, and relevance before using it to inform a decision. This term is used for items 12 and 19 instead of "risk of bias" (which is more applicable to systematic reviews of interventions) to include and acknowledge the various sources of evidence that may be used in a scoping review (e.g., quantitative and/or qualitative research, expert opinion, and policy document).

From: Tricco AC, Lillie E, Zarin W, O'Brien KK, Colquhoun H, Levac D, et al. PRISMA Extension for Scoping Reviews (PRISMAScR): Checklist and Explanation. Ann Intern Med. 2018;169:467–473. doi: 10.7326/M18-0850.

For peer review only - http://bmjopen.bmj.com/site/about/guidelines.xhtml
